# Novel Mono-Substituted 4*H*-1,2,6-Thiadiazines with Antioxidant and Anti-Lipoxygenase Activities

**DOI:** 10.3390/ijms262411817

**Published:** 2025-12-07

**Authors:** Eleftherios Charissopoulos, Panayiotis A. Koutentis, Andreas S. Kalogirou, Eleni Pontiki

**Affiliations:** 1 Laboratory of Pharmaceutical Chemistry, Faculty of Health Sciences, School of Pharmacy, Aristotle University of Thessaloniki, 54124 Thessaloniki, Greece; echariss@pharm.auth.gr; 2 Department of Chemistry, University of Cyprus, P.O. Box 20537, Nicosia 1678, Cyprus; koutenti@ucy.ac.cy; 3 Department of Life Sciences, School of Sciences, European University Cyprus, 6 Diogenis Str., Engomi, P.O. Box 22006, Nicosia 1516, Cyprus

**Keywords:** heterocycle, 1,2,6-thiadiazine, antioxidants, lipoxygenase, anti-inflammatory

## Abstract

Τhe synthesis of a novel series of mono-substituted 4*H*-1,2,6-thiadiazine derivatives was reported, aiming at enhancing antioxidant and lipoxygenase inhibitory activities via pharmacophore combination. The compounds were prepared from 3,5-dichloro-4*H*-1,2,6-thiadiazin-4-one and 2-(3,5-dichloro-4*H*-1,2,6-thiadiazin-4-ylidene)malononitrile. All the derivatives were evaluated for radical scavenging activity towards 2,2-diphenyl-1-picrylhydrazyl (DPPH), 2,2′-azobis(2-methylpropionamidine) dihydrochloride (AAPH)-induced lipid peroxidation inhibition, and soybean lipoxygenase (sLOX) inhibition. The compounds exhibited moderate to good antioxidant activity and variable sLOX inhibition. Notably, 2-[3-(benzo[*d*]oxazol-2-ylthio)-5-chloro-4*H*-1,2,6-thiadiazin-4-ylidene]malononitrile showed the strongest antioxidant effect (92% DPPH scavenging at 60 min and 70% inhibition of AAPH-induced lipid peroxidation) but low sLOX inhibition, whereas 3-chloro-5-(4-phenylpiperazin-1-yl)-4*H*-1,2,6-thiadiazin-4-one displayed the most potent sLOX inhibition (IC_50_: 7.5 μM), with a docking score of −8.3 kcal/mol developing hydrophobic interactions with Phe134 and Val520.

## 1. Introduction

### 1.1. Interplay Between Inflammation and Oxidative Stress

**Figure 1:**
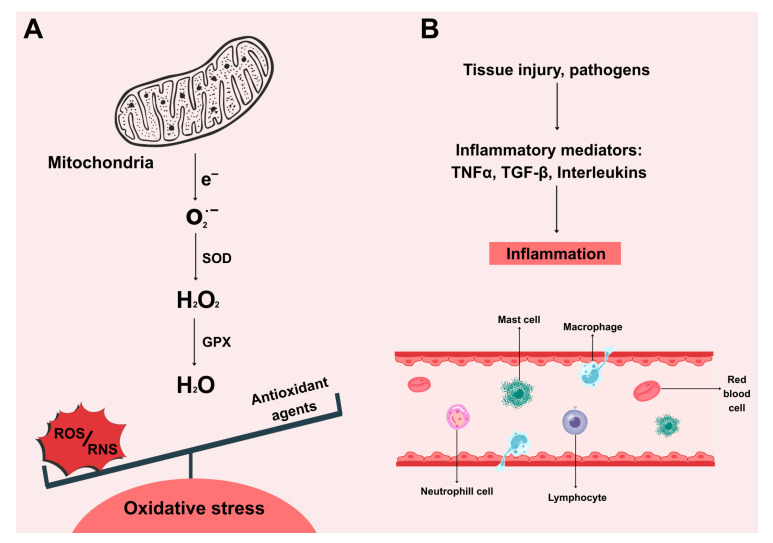
(**A**) Schematic representation of mitochondrial involvement in oxidative stress. Molecular oxygen (O_2_) undergoes partial reduction, generating superoxide anion (O_2_^.−^). O_2_^.−^ is then converted into hydrogen peroxide (H_2_O_2_) by superoxide dismutase (SOD), which is subsequently reduced to water (H_2_O) by glutathione peroxidase (GPX). This pathway shows how mitochondria balance ROS production with antioxidant defenses. (**B**) Schematic overview of the inflammatory response. The figure illustrates the recruitment and activation of mast cells, macrophages, neutrophils, and lymphocytes, and the presence of red blood cells within the vasculature.

Oxidative stress (OS) and inflammation are interconnected processes that not only serve protective roles but are also essential in maintaining physiological homeostasis [1]. Inflammation is a fundamental response of the immune system, typically triggered by invading pathogens or physical injury (Figure 1) [2,3,4]. The main microcirculatory events of the inflammatory phase include increased vascular permeability, leukocyte recruitment and accumulation, and the release of inflammatory mediators [5]. Oxidative stress occurs when the balance between oxidants and antioxidants is disrupted (Figure 1), resulting in tissue damage [6]. During OS, inflammatory mediators are generated, which in turn enhance the production of reactive oxygen species (ROS) [7,8]. Targeting the excessive generation of reactive oxygen species (ROS) has emerged as a clinically significant strategy for managing diseases driven by chronic inflammation [9].

### 1.2. 1,2,6-Thiadiazines: Structure and Biological Functions

**Scheme 1:**
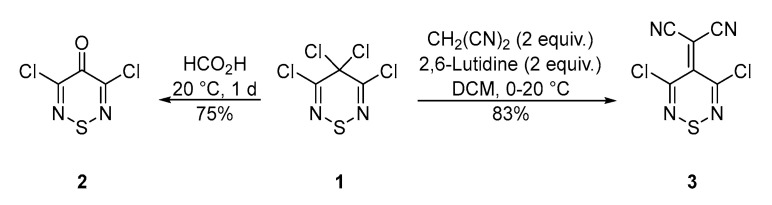
Formation of thiadiazines **2** and **3**.

1,2,6-Thiadiazines are sulfur–nitrogen heterocycles with diverse applications [10]. Non-S-oxidized 4*H*-1,2,6-thiadiazines are rare, but find uses in the biological [11,12,13,14,15,16] as well as the materials sciences [17,18,19,20,21,22]. Their chemistry and applications have recently been reviewed [23]. Current research on thiadiazines primarily centers on three key starting materials, 3,4,4,5-tetrachloro-4*H*-1,2,6-thiadiazine (**1**), ketone **2** (see Figure S1 in the Supporting Information for its synthesis, SI), and ylidenemalononitrile **3** (Scheme 1).

**Scheme 2:**
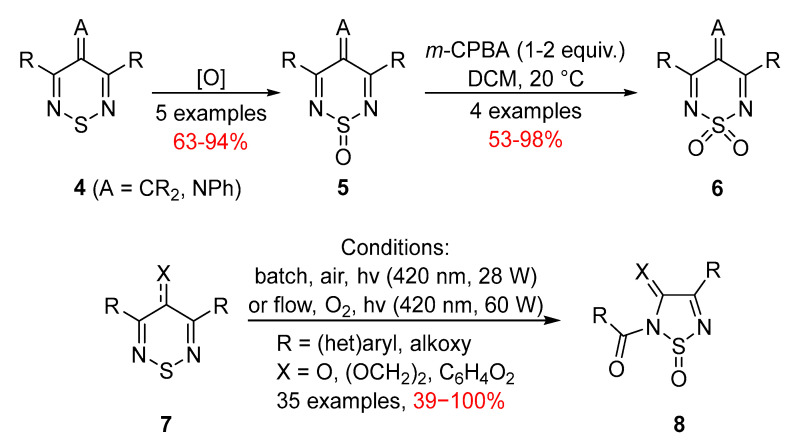
Oxidations of selected 4*H*-1,2,6-thiadiazinones **4** and **7** [22,23].

This research work is focused on mono-substituted 1,2,6-thiadiazines as antioxidants. A small library of monosubstituted 3-amino and 3-thiothiadiazines (Table 1 and Table 2) was investigated. Interestingly, 1,2,6-thiadiazines **4** can be oxidized by a range of oxidants (N_2_O_4_, *m*-CPBA, Oxone, PIFA, and PIDA) to give oxidation of the endocyclic sulfur to either sulfoxides **5** or sulfones **6** [24] (Scheme 2). Moreover, the photochemical treatment of 4*H*-1,2,6-thiadiazines **7** with visible light and air (or ^3^O_2_) afforded ring-contracted 1,2,5-thiadiazole 1-oxides **8** in good yields under both batch and flow conditions [23,25] (Scheme 2, see Figure S2, SI for the reaction mechanism).

**Scheme 3:**
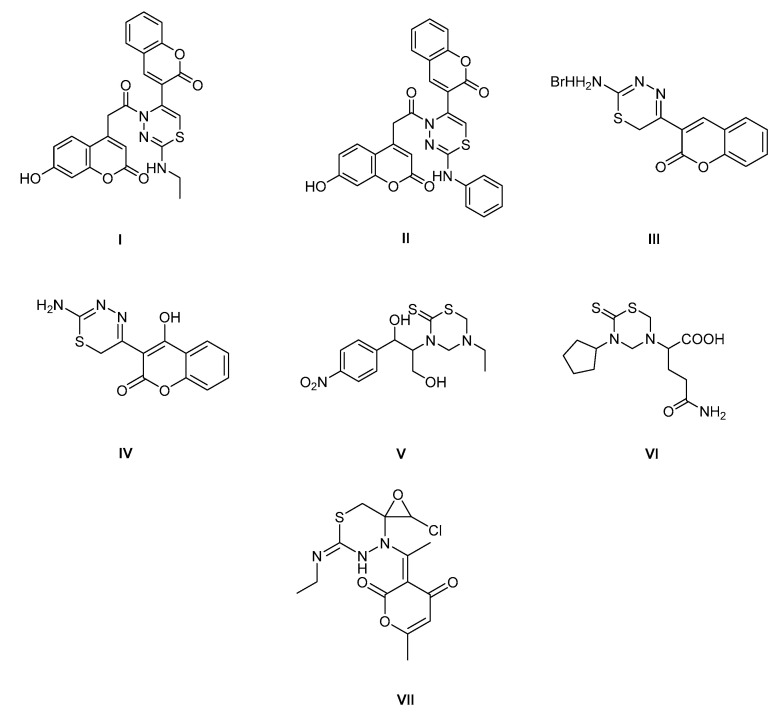
Structures of thiadiazine derivatives exhibiting potential antiparasitic, antioxidant, anticancer, antibacterial, and antifungal activities (**I*–*VII**) [26,27,28].

Additionally, other thiadiazine isomers have previously been explored for a broad spectrum of biological activities. For example, a series of 1,3,4-thiadiazine–coumarin hybrids has been investigated for their antioxidative and antifungal properties. Other thiadiazine derivatives have been reported as potential antiparasitic, antioxidant, anticancer, antibacterial, and antifungal agents (Scheme 3) [26,27,28]. Compounds **I**–**IV** were evaluated for DPPH scavenging activity and antifungal effects against *A. flavus*, showing promising results [27]. Compound **V** exhibited notable antibacterial and antifungal properties [26], while compounds **VI** [26] and **VII** [28] emerged as promising candidates for future anticancer and antiparasitic studies, respectively. Recent studies have also highlighted that thiadiazines, due to their resemblance to cephalosporins, can play a pivotal role in bacterial resistance by inhibiting the NorA pump [28].

In light of these findings, a series of 1,2,6-thiadiazines was assessed for their capacity to (i) neutralize the stable DPPH free radical; (ii) inhibit linoleic acid peroxidation, and (iii) inhibit soybean lipoxygenase as a marker of anti-inflammatory activity.

## 2. Results and Discussion

### 2.1. Synthesis

Ιnitially the selective mono-displacement of dichlorothiadiazines **2** and **3** with selected amine nucleophiles was investigated. N-Substituted piperazines, such as 1-phenylpiperazine and 1-benzhydrylpiperazine, were selected. Piperazines are privileged scaffolds [29,30] in drug discovery, as they often improve the solubility and pharmacokinetic properties of bioactive molecules. The reactions proceeded smoothly, affording four new 3-aminothiadiazines **9a**,**b** and **10a**,**b** in high yields (Table 1).

**Table 1:** Synthesis of 3-aminothiadiazine targets.

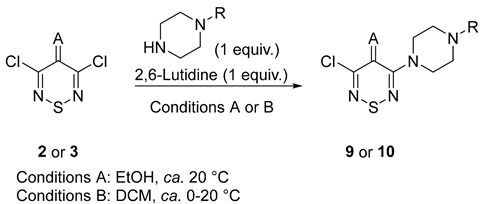				
Compound	A	R	Conditions (Time, h)	Yield (%)
**9a**	O	Ph	A (1)	97
**9b**	O	CHPh_2_	A (1)	99
**10a**	C(CN)_2_	Ph	B (1)	88
**10b**	C(CN)_2_	CHPh_2_	B (1)	100

Subsequently, the mono-displacement of dichlorothiadiazines **2** and **3** with a number of aromatic and heteroaromatic thiols to prepare 3-thiothiadiazines was explored. Interestingly, dichlorothiazinone **2** gave good yields of sulfides **11** (43–81%) with the exception of tetrazole thiols (28 and 39%, Table 2, **11b** & **11e**) and 6-ethoxybenzothiazol thiol (43%, Table 2, **11i**) that gave low yields. The three examples of thiol displacement of dicyanoylidene **3** all gave medium yields of sulfides **12** (49–71%, Table 2, **12a**–**c**). Compound **11h** has been previously reported in the literature [31]. For all the compounds, the identification was carried out by melting point, IR, MS, and ^1^H and ^13^C NMR (see SI for NMR spectra).

**Table 2:** Synthesis of 3-thiothiadiazine targets.

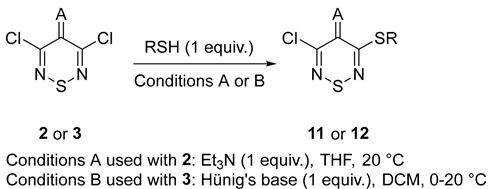				
Compound	A	R	Time (h)	Yield (%)
**11a**	O	4-Me-pyrimid-2-yl	24	81
**11b**	O	1-Methyl-1*H*-tetrazol-5-yl	5	28
**11c**	O	Benzo[*d*]oxazol-2-yl	20	74
**11d**	O	4-Phenylacetamide	1	81
**11e**	O	1-Ph-1*H*-tetrazol-5-yl	5	39
**11f**	O	4-Me-4*H*-1,2,4-triazol-3-yl	3	81
**11g**	O	Thiazol-2-yl	1	73
**11h**	O	Benzo[*d*]thiazol-2-yl	24	71 ^a^
**11i**	O	6-EtO-benzo[*d*]thiazol-2-yl	2	43
**12a**	C(CN)_2_	4-Me-pyrimid-2-yl	0.5	71
**12b**	C(CN)_2_	1-Me-1*H*-tetrazol-5-yl	3.5	56
**12c**	C(CN)_2_	Benzo[*d*]oxazol-2-yl	3	49

^a^ Prepared according to the literature [31].

### 2.2. Physicochemical Studies

#### 2.2.1. Determination of Lipophilicity

Lipophilicity is an important property of a drug candidate, as it affects solubility and how easily the molecule crosses cell membranes, which in turn influences its ADMET profile (absorption, distribution, metabolism, elimination, and toxicity) [32,33]. Lipophilicity (expressed as clogP values) was theoretically calculated using the Bio-Loom program from BioByte Corp (http://www.biobyte.com/, accessed on 10 June 2025).

#### 2.2.2. Theoretical Calculation of Physicochemical Properties

Drug-likeness refers to the likelihood that a molecule can become an orally bioavailable drug. In practice, it is evaluated by comparing the physicochemical and structural properties of a new compound to those of established drugs. ADMET profiles can often be estimated directly from a molecule’s structure, ideally even before its synthesis and biological testing. To support this, numerous in silico tools have been established for predicting ADMET characteristics. Among these is the online platform Molinspiration (www.molinspiration.com, Molinspiration Cheminformatics 2025, 9 September 2025). On this platform, drug-likeness is evaluated using Lipinski’s Rule of Five. The parameters calculated include miLogP (lipophilicity); TPSA (topological polar surface area), an important indicator of drug absorption and bioavailability; Natoms (total atom count); MW (molecular weight), which influences absorption, distribution, and overall drug-likeness; nON (hydrogen bond acceptors); nOHNH (hydrogen bond donors), which affect hydrogen-bonding potential and target affinity; Nviolations (number of Lipinski’s rule of five violations); Nrotb (rotatable bonds), reflecting molecular flexibility and oral bioavailability; and Volume (three-dimensional molecular size), impacting membrane permeability and steric compatibility. All the synthesized compounds do not present any violations. The results are presented in Table 3.

### 2.3. Biological Evaluation

The synthesized compounds were evaluated in vitro for their ability to interact with the stable free radical DPPH, inhibit the AAPH-induced linoleic acid peroxidation, and suppress soybean lipoxygenase activity.

The synthesized derivatives were tested for their interaction with the stable free radical 2,2-diphenyl-1-picrylhydrazyl (DPPH) at a concentration of 100 μM after 20 and 60 min (Table 4). DPPH is widely used as a standard method for evaluating antioxidant activity [34]. Interestingly, the interaction between lipophilic compounds and DPPH is not dictated by lipophilicity alone. The antioxidant’s molecular structure, the solvent environment, and several experimental conditions can significantly influence this behavior. However, lipophilic compounds typically dissolve efficiently in organic solvents when they react with DPPH. The relationship between antioxidant activity and lipophilicity was reported in previous studies [35]. Nordihydroguaiaretic acid (NDGA) was used as a reference standard [36].

Among the **9a**,**b**, **10a**,**b**, and **11a**–**i** series, none of the compounds exhibited significant DPPH radical scavenging activity at 20 or 60 min. Moreover, the interaction with DPPH was not time-dependent. Among these series, compound **11c** showed the highest activity, with 21% at 20min, which seems to be stable over time (22% interaction at 60 min). From the **11** series, compound **11c**, which contains the benzo[*d*]oxazol-2-yl substitution, presented the highest interaction with DPPH, but still moderate compared to the reference compound.

Compounds **12a**–**c** exhibited the most potent activity, with compounds **12a** and **12c** showing excellent interactions of 90% and 92%, respectively, at 60 min, compared to NDGA (87%). The interaction of compounds **12a**–**c** with the DPPH radical appears to increase over time. Compound **12c**, which contains a benzo[*d*]oxazol-2-yl moiety, again emerged as the most active compound, similar to the **11**-series. The results indicate that the presence of a 4-dicyanoylidine group on the thiadiazine instead of a ketone enhances DPPH interaction, as evidenced by the greater increase in % interaction values observed for compounds **12a**–**c** compared to the corresponding compounds **11a**–**c**.

In the lipid peroxidation assay, sodium linoleate was used as the substrate, while 2,2′-azobis(2-methylpropionamidine) dihydrochloride (AAPH) was the peroxyl radical initiator. The formation of 13-hydroperoxy-linoleic acid from linoleic acid was observed by UV-Vis absorption measurements at 234 nm. Trolox was used as a reference compound.

Most of the synthesized compounds exhibited moderate to low inhibitory activity, except for compound **12c**, with 70% anti-lipid peroxidation.

Among the **9a**,**b** and **10a**,**b** series, all of them showed low inhibitory activity, with compound **10a** having the best one with 27%. Among the **11a**–**i** series, the compounds exhibited moderate to low activity, with **11b** and **11d** showing the highest values of 45% and 43%, respectively. Among the **12a**–**c** series, compound **12c**, which contains the benzo[*d*]oxazol-2-yl moiety, showed excellent AAPH inhibitory effect (70%). Comparison between the **11a**–**c** and **12a**–**c** series revealed that the presence of a 4-dicyanoylidine group in the thiadiazine instead of a ketone favors AAPH inhibition, with the exception of the comparison between compounds **11b** and **12b**.

Lipoxygenase (LOX) plays a key role in the formation of leukotrienes, which are important inflammatory mediators [37]. Leukotrienes are considered key therapeutic targets in inflammatory diseases due to their role. Soybean lipoxygenase (LOX), a plant-based enzyme, has long been used as a model system to study the structural and functional characteristics of the lipoxygenase family [38] due to its significant homology with human 5-LOX [39]. NDGA was used as a reference drug.

Based on the IC_50_ values, the compounds generally exhibited moderate to low inhibitory effects, with the exception of compound **9a**, which presented an IC_50_ value of 7.5 µM and A: O and R: Ph substitution. Comparison of the pairs **9a**,**b** and **10a**,**b** suggests that in this case, the presence of a 4-ketone group in the thiadiazine instead of a dicyanoylidine is more favorable for sLOX inhibition. Among the **11a**–**i** series, compounds **11f**, **11i**, **11c**, and **11d** exhibited moderate inhibitory effects, with IC_50_ values of 52.5, 67.5, 77.5, and 85 µM, respectively. Compound **11f**, bearing a 4-Me-4*H*-1,2,4-triazol-3-yl substitution, presented slightly better results within the **11**-series with an IC_50_ of 52.5 µM. Comparison of the **11a**–**c** and **12a**–**c** series suggests that within these series, the presence of a 4-dicyanoylidine group in the thiadiazine instead of a ketone enhances the anti-LOX inhibition, with the exception of compounds **11c** and **12c**.

### 2.4. Docking Simulation Soybean Lipoxygenase Studies

Soybean lipoxygenase-1(PDB ID: 3PZW) was selected for the docking studies. The crystal structure of soybean lipoxygenase-1 (PDB ID: 3PZW) lacks a co-crystallized ligand. Thus, potential allosteric binding pockets outside the established iron-binding and substrate-binding sites have been identified, as highlighted in recent studies. Detsi, A. et al. [40] have detected three potential binding sites using SiteMap’s [41]. Researchers have identified Site 1 between the amino-terminal β-barrel (PLAT domain) and the α-helical domain that contains the catalytic iron. This is recognized as a potential binding site in blind docking studies [40]. Sites 2 and 3 are positioned within the α-helical segment as well.

**Figure 2:**
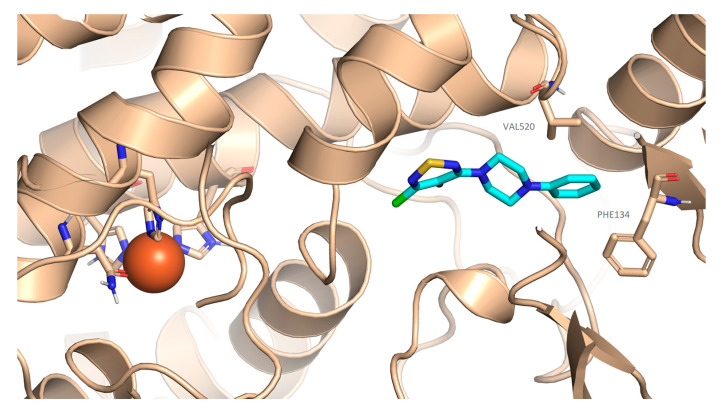
The 3D preferred docking pose of compound **9a** (depicted in cyan) bound to soybean lipoxygenase. Iron appears as an orange sphere.

Consistent with previous studies, blind docking was performed to explore all potential binding sites. The results indicate that the compounds preferentially bind to Site 1, which aligns with the findings discussed earlier. Compound **9a** was the most potent, presenting a binding score of −8.3 kcal/mol. Compound **9a** develops hydrophobic interactions with the amino acids Phe134 and Val520 (Figure 2).

## 3. Materials and Methods

### 3.1. Materials and Instruments

The reaction mixture was monitored by TLC using commercial glass-backed thin-layer chromatography (TLC) plates (Merck Kieselgel 60 F_254_, Merck, Darmstadt, Germany). The plates were observed under UV light at 254 and 365 nm. Tetrahydrofuran (THF) was distilled over CaH_2_ before use. Melting points were determined using a PolyTherm-A, Wagner & Munz, Kofler Hotstage Microscope apparatus (Wagner & Munz, Munich, Germany). The solvent used for recrystallization is indicated after the melting point. The UV-vis spectrum was obtained using a Perkin-Elmer Lambda-25 UV/vis spectrophotometer (Perkin-Elmer, Waltham, MA, USA), and inflections are identified by the abbreviation “inf”. The IR spectrum was recorded on a Shimadzu FTIR-NIR Prestige-21 spectrometer (Shimadzu, Kyoto, Japan) with Pike Miracle Ge ATR accessory (Pike Miracle, Madison, WI, USA), and strong, medium, and weak peaks are represented by s, m, and w, respectively. ^1^H and ^13^C NMR spectra were recorded on a Bruker Avance 300 (at 300 and 75 MHz, respectively), or a 500 machine (at 500 and 125 MHz, respectively) (Bruker, Billerica, MA, USA). Deuterated solvents were used for homonuclear lock, and the signals are referenced to the deuterated solvent peaks. Attached proton test (APT) NMR studies were used for the assignment of the ^13^C peaks as *C*H_3_, *C*H_2_, *C*H, and *C*q (quaternary). The Matrix-Assisted Laser Desorption/Ionization-Time of Flight (MALDI-TOF) mass spectrum (+ve mode) was recorded on a Bruker Autoflex III Smartbeam instrument (Bruker), while ESI-APCI^+^ mass spectra were recorded on a Model 6110 Quadrupole MSD, Agilent Technologies (Agilent, Santa Clara, CA, USA), and ES-API spectra on a Model 1260 Infinity II Quadrupole MSD, Agilent Technologies. The elemental analysis was run by the London Metropolitan University Elemental Analysis Service. 3,5-Dichloro-4*H*-1,2,6-thiadiazin-4-one (**2**) [42], 2-(3,5-dichloro-4*H*-1,2,6-thiadiazin-4-ylidene)malononitrile (**3**) [43], and 3-(benzo[*d*]thiazol-2-ylthio)-5-chloro-4*H*-1,2,6-thiadiazin-4-one (**11h**) [26] were prepared according to the literature procedures.

### 3.2. Chemistry General Procedure

#### 3.2.1. Synthesis of 3-Aminothiadiazines (**9a**,**b**, **10a**,**b**, **11a**–**i**, **12a**–**c**)

##### 3-Chloro-5-(4-phenylpiperazin-1-yl)-4*H*-1,2,6-thiadiazin-4-one (**9a**)

To a stirred mixture of 3,5-dichloro-4*H*-1,2,6-thiadiazin-4-one (**2**) (92.0 mg, 0.50 mmol) in EtOH (1 mL) at *ca*. 20 °C, *N*-phenylpiperazine was added (76 *μ*L, 0.50 mmol), followed by 2,6-lutidine (58 *μ*L, 0.50 mmol), and the mixture was stirred at this temperature until complete consumption of the starting material (TLC, 1 h). The precipitate formed was then filtered, washed with EtOH (1 mL), then *t*-BuOMe (1 mL), and dried to give the title compound **9a** (82.6 mg, 53%). The filtrate was then adsorbed onto silica and chromatographed (*n*-hexane/DCM 50:50) to give a further quantity of the title compound **9a** (68 mg, total yield 97%) as yellow needles, mp 166–167 °C (from *c*-hexane); R*_f_* 0.48 (*n*-hexane/DCM 50:50); (found: C, 50.47; H, 4.07; N, 18.07. C_13_H_13_ClN_4_OS requires C, 50.57; H, 4.24; N, 18.14%); *λ*_max_ (DCM)/nm 255 (log *ε* 4.21), 312 (4.21), 320 inf (4.18), 404 (3.74); *v*_max_/cm^−1^ 2901w and 2833w (C-H), 1624s (C=O), 1595m, 1497s, 1344s, 1389m, 1366m, 1277m, 1233s, 1157m, 1094w, 1055w, 1040m, 989m, 955m, 922m, 872s, 854s, 752s; *δ*_H_ (300 MHz; CDCl_3_) 7.33–7.27 (2H, m, Ar C*H*), 6.93–6.89 (3H, m, Ar C*H*), 4.08 (4H, t, J 4.8, Ar C*H*_2_), 3.29 (4H, t, *J* 5.3, Ar C*H*_2_); *δ*_C_ (75 MHz; CDCl_3_) 158.7 (*C*q), 152.8 (*C*q), 150.7 (*C*q), 145.4 (*C*q), 129.3 (*C*H), 120.6 (*C*H), 116.4 (*C*H), 49.3 (*C*H_2_), 46.6 (*C*H_2_); *m*/*z* (APCI^+^) 311 (MH^+^+2, 35%), 309 (MH^+^, 100), 190 (8), 163 (8), 108 (10), 104 (11).

##### 3-(4-Benzhydrylpiperazin-1-yl)-5-chloro-4*H*-1,2,6-thiadiazin-4-one (**9b**)

To a stirred mixture of 3,5-dichloro-4*H*-1,2,6-thiadiazin-4-one (**2**) (92.0 mg, 0.50 mmol) in EtOH (1 mL) at ca. 20 °C, 1-benzhydrylpiperazine was added (126 mg, 0.50 mmol), followed by 2,6-lutidine (58 *μ*L, 0.50 mmol), and the mixture was stirred at this temperature until complete consumption of the starting material (TLC, 1 h). The mixture was then adsorbed onto silica and chromatographed (*n*-hexane/DCM 70:30) to give the title compound **9b** (197 mg, 99%) as yellow needles, mp 56–57 °C (from EtOH/H_2_O); R*_f_* 0.24 (*n*-hexane/DCM 70:30); (found: C, 60.45; H, 4.66; N, 14.01. C_20_H_19_ClN_4_OS requires C, 60.22; H, 4.80; N, 14.05%); *λ*_max_ (DCM)/nm 268 (log *ε* 3.98), 312 (4.21), 322 (4.18), 408 (3.76); *v*_max_/cm^−1^ 3021w (C-H arom), 2810w (C-H alip), 1626s (C=O), 1503s, 1441m, 1300m, 1283m, 1260s, 1190s, 1123m, 1142m, 1103m, 1076m, 1051m, 1030m, 995s, 953s, 922m, 876s, 854s, 838m, 764s, 746s, 725m, 704s; *δ*_H_ (500 MHz; CDCl_3_) 7.42 (4H, d, *J* 7.2, Ar C*H*), 7.29 (4H, dd, *J* 7.4, 7.4, Ar C*H*), 7.20 (2H, dd, *J* 7.4, 7.4, Ar C*H*), 4.25 (1H, s, NC*H*), 3.90 (4H, s, C*H*_2_), 2.49 (4H, t, *J* 5.0, C*H*_2_); *δ*_C_ (125 MHz; CDCl_3_) 158.7 (*C*q), 152.8 (*C*q), 145.0 (*C*q), 142.0 (*C*q), 128.6 (*C*H), 127.9 (*C*H), 127.2 (*C*H), 76.0 (*C*H), 51.8 (*C*H_2_), 46.9 (*C*H_2_); *m*/*z* (ESI^+^) 423 (M+Na^+^+2, 5), 421 (M+Na^+^, 14), 401 (MH^+^+2, 3%), 399 (MH^+^, 7), 381 (27), 325 (36), 167 (Ph_2_CH^+^, 100).

##### 2-[3-Chloro-5-(4-phenylpiperazin-1-yl)-4*H*-1,2,6-thiadiazin-4-ylidene]malononitrile (**10a**)

To a stirred mixture of 2-(3,5-dichloro-4*H*-1,2,6-thiadiazin-4-ylidene)malononitrile (**3**) (116 mg, 0.50 mmol) in DCM (2 mL) at *ca*. 0 °C, *N*-phenylpiperazine was added (76 *μ*L, 0.50 mmol), followed by 2,6-lutidine (58 *μ*L, 0.50 mmol), and the mixture was left to warm to *ca*. 20 °C and stirred until complete consumption of the starting material (TLC, 1 h). The mixture was then adsorbed onto silica and chromatographed (*n*-hexane/DCM 50:50) to give the title compound **10a** (157 mg, 88%) as red plates, mp 114–115 °C (from EtOH/H_2_O); R*_f_* 0.40 (*n*-hexane/DCM 50:50); (found: C, 53.92; H, 3.45; N, 23.39. C_16_H_13_ClN_6_O requires C, 53.86; H, 3.67; N, 23.55%); *λ*_max_ (DCM)/nm 251 (log *ε* 4.30), 330 (3.98), 491 (3.97); *v*_max_/cm^−1^ 2955w, 2918w, 2889w and 2849w (C-H), 2210 (C≡N), 1597m, 1493s, 1479s, 1435s, 1391m, 1385m, 1364m, 1337m, 1294m, 1267m, 1227s, 1188w, 1150m, 1069m, 1034w, 999m, 935s, 916m, 851m, 826s, 775m, 760s, 733s; *δ*_H_ (500 MHz; CDCl_3_) 7.30 (2H, dd, *J* 8.6, 7.3, Ar C*H*), 6.96–6.92 (3H, m, Ar C*H*), 3.75 (2H, br. s, C*H*_2_), 3.84 (6H, br. s, C*H*_2_); *δ*_C_ (125 MHz; CDCl_3_) 150.5 (*C*q), 147.6 (*C*q), 134.4 (*C*q), 134.0 (*C*q), 129.3 (*C*H), 121.0 (*C*H), 116.9 (*C*H), 113.3 (*C*q), 112.3 (*C*q), 75.6 (*C*q), 48.3 (*C*H_2_), 48.0 (*C*H_2_); *m*/*z* (APCI^+^) 359 (MH^+^+2, 33%), 357 (MH^+^, 100), 238 (10), 146 (16).

##### 2-[3-(4-Benzhydrylpiperazin-1-yl)-5-chloro-4*H*-1,2,6-thiadiazin-4-ylidene]malononitrile (**10b**)

To a stirred mixture of 2-(3,5-dichloro-4*H*-1,2,6-thiadiazin-4-ylidene)malononitrile (**3**) (116 mg, 0.50 mmol) in DCM (2 mL) at *ca*. 0 °C, 1-benzhydrylpiperazine was added (126 mg, 0.50 mmol), followed by 2,6-lutidine (58 *μ*L, 0.50 mmol), and the mixture was left to warm to *ca*. 20 °C and stirred until complete consumption of the starting material (TLC, 1 h). The mixture was then adsorbed onto silica and chromatographed (*n*-hexane/DCM 50:50) to give the title compound **10b** (223 mg, 100%) as orange needles, mp 204–205 °C (from EtOH); R*_f_* 0.39 (*n*-hexane/DCM 50:50); (found: C, 61.97; H, 4.13; N, 18.56. C_23_H_19_ClN_6_S requires C, 61.81; H, 4.28; N, 18.80%); *λ*_max_ (DCM)/nm 249 inf (log *ε* 3.97), 330 (4.06), 381 inf (3.66), 499 (4.04); *v*_max_/cm^−1^ 3071w, 3040w and 3024w (C-H arom), 2997w, 1984w, 2957w, 2912w and 2886 (C-H alip), 2214m (C≡N), 1516s, 1498s, 1446m, 1437s, 1400m, 1342w, 1308m, 1300m, 1290m, 1273m, 1256w, 1234m, 1207w, 1188m, 1150m, 1140m, 1113m, 1103m, 1074m, 1067m, 1055w, 1030w, 991m, 968w, 932m, 864m, 854m, 827m, 814s, 777m, 756m, 745m, 729s, 704s; *δ*_H_ (500 MHz; CDCl_3_) 7.41 (4H, d, *J* 7.5, Ar C*H*), 7.28 (4H, dd, *J* 7.4, 7.4, Ar C*H*), 7.20 (2H, dd, *J* 7.4, 7.4, Ar C*H*), 4.28 (1H, s, NC*H*), 3.60 (2H, br. s, C*H*_2_), 3.33 (2H, br. s, C*H*_2_), 2.59 (4H, br. s, C*H*_2_); *δ*_C_ (125 MHz; CDCl_3_) 147.4 (*C*q), 142.0 (*C*q), 134.1 (*C*q), 134.0 (*C*q), 128.7 (*C*H), 127.8 (*C*H), 127.3 (*C*H), 113.3 (*C*q), 112.4 (*C*q), 75.8 (*C*H), 75.2 (*C*q), 50.5 (*C*H_2_), 48.3 (*C*H_2_); *m*/*z* (ESI^+^) 449 (MH^+^+2, 3%), 447 (MH^+^, 5), 381 (5), 325 (15), 167 (Ph_2_CH^+^, 100).

#### 3.2.2. Synthesis of 3-thiothiadiazines

##### 3-Chloro-5-[(4-methylpyrimidin-2-yl)thio]-4*H*-1,2,6-thiadiazin-4-one (**11a**)

To a stirred mixture of 3,5-dichloro-4*H*-1,2,6-thiadiazin-4-one (**2**) (92.0 mg, 0.50 mmol) in THF (2 mL) at *ca*. 20 °C, 4-methylpyrimidine-2-thiol was added (63.0 mg, 0.50 mmol), followed by Et_3_N (69 *μ*L, 0.50 mmol), and the mixture was stirred at this temperature until complete consumption of the starting material (TLC, 24 h). The mixture was then adsorbed onto silica and chromatographed (DCM) to give the title compound **11a** (111 mg, 81%) as yellow needles, mp 119–120 °C (from DCE/*c*-hexane); R*_f_* 0.43 (DCM); (found: C, 35.36; H, 1.72; N, 20.39. C_8_H_5_ClN_4_OS_2_ requires C, 35.23; H, 1.85; N, 20.54%); *λ*_max_ (DCM)/nm 241 inf (log *ε* 4.10), 285 inf (4.13), 315 (4.32), 357 (4.22); *v*_max_/cm^−1^ 1643s (C=O), 1572s, 1534w, 1491m, 1431w, 1416m, 1371w, 1339m, 1281m, 1273m, 1240w, 1200m, 1186m, 1099w, 1059m, 1045w, 899w, 878w, 856m, 849m, 762m, 736s, 721s; *δ*_H_ (300 MHz; DMSO-*d*_6_) 8.68 (1H, d, *J* 5.4, Ar C*H*), 7.41 (1H, d, *J* 5.0, Ar C*H*), 2.47 (3H, s, C*H*_3_); *δ*_C_ (75 MHz; DMSO-*d*_6_) 169.1 (*C*q), 163.7 (*C*q), 160.3 (*C*q), 158.8 (*C*q), 158.4 (*C*H), 145.4 (*C*q), 120.2 (*C*H), 23.4 (*C*H_3_); *m*/*z* (APCI^+^) 275 (MH^+^+2, 39%), 273 (MH^+^, 100), 237 (10).

##### 3-Chloro-5-[(1-methyl-1*H*-tetrazol-5-yl)thio]-4*H*-1,2,6-thiadiazin-4-one (**11b**)

To a stirred mixture of 3,5-dichloro-4*H*-1,2,6-thiadiazin-4-one (**2**) (92.0 mg, 0.50 mmol) in THF (2 mL) at *ca*. 20 °C, 1-methyl-1*H*-tetrazole-5-thiol was added (58.0 mg, 0.50 mmol), followed by Et_3_N (69 *μ*L, 0.50 mmol), and the mixture was stirred at this temperature until complete consumption of the starting material (TLC, 5 h). The mixture was then adsorbed onto silica and chromatographed (DCM) to give the title compound **11b** (36 mg, 28%) as yellow needles, mp 215–216 °C (from EtOH); R*_f_* 0.42 (DCM); (found: C, 22.73; H, 1.38; N, 31.75. C_5_H_3_ClN_6_OS_2_ requires C, 22.86; H, 1.15; N, 31.99%); *λ*_max_ (DCM)/nm 305 (log *ε* 4.17), 347 (4.23); *v*_max_/cm^−1^ 1634s (C=O), 1493m, 1470w, 1396w, 1285m, 1263w, 1244w, 1204w, 1180m, 1065s, 984w, 932w, 905w, 862m, 748s, 721m; *δ*_H_ (500 MHz; DMSO-*d*_6_) 4.04 (3H, s, C*H*_3_); *δ*_C_ (125 MHz; DMSO-*d*_6_) 159.2 (*C*q), 158.1 (*C*q), 146.3 (*C*q), 144.4 (*C*q), 34.5 (*C*H_3_); *m*/*z* (APCI^+^) 264 (M^+^+2, 3%), 262 (M^+^, 5), 120 (100).

##### 3-(Benzo[*d*]oxazol-2-ylthio)-5-chloro-4*H*-1,2,6-thiadiazin-4-one (**11c**)

To a stirred mixture of 3,5-dichloro-4*H*-1,2,6-thiadiazin-4-one (**2**) (92.0 mg, 0.50 mmol) in THF (2 mL) at *ca*. 20 °C, benzo[*d*]oxazole-2-thiol was added (76.0 mg, 0.50 mmol), followed by Et_3_N (69 *μ*L, 0.50 mmol), and the mixture was stirred at this temperature until complete consumption of the starting material (TLC, 20 h). The solvent was then evaporated under vacuum, EtOH (5 mL) added and the precipitate filtered, washed with EtOH (1 mL) and dried to give the title compound **11c** (110 mg, 74%) as brown needles, mp 277–279 °C (from PhMe/DMA); R*_f_* 0.71 (DCM); (found: C, 40.26; H, 1.47; N, 13.96. C_10_H_4_ClN_3_O_2_S_2_ requires C, 40.34; H, 1.35; N, 14.11%); *λ*_max_ (DCM)/nm 293 inf (log *ε* 4.20), 313 (4.32), 346 inf (4.17); *v*_max_/cm^−1^ 1645s (C=O), 1504m, 1497m, 1472w, 1449m, 1337w, 1300m, 1287w, 1248m, 1213m, 1123m, 1094s, 1067w, 1003w, 934m, 891w, 804m, 760s, 750s, 729s; *δ*_H_ (500 MHz; DMSO-*d*_6_) 7.89 (1H, d, *J* 7.8, Ar C*H*), 7.84 (1H, d, *J* 8.2, Ar C*H*), 7.54 (1H, ddd, *J* 7.7, 7.7, 1.1, Ar C*H*), 7.48 (1H, ddd, *J* 7.7, 7.7, 0.8, Ar C*H*); *δ*_C_ (125 MHz; DMSO-*d*_6_) 159.4 (*C*q), 158.9 (*C*q), 154.2 (*C*q), 152.3 (*C*q), 144.6 (*C*q), 141.2 (*C*q), 126.9 (*C*H), 125.2 (*C*H), 120.3 (*C*H), 111.2 (*C*H); *m*/*z* (MALDI-TOF) 300 (MH^+^+2, 4%), 298 (MH^+^, 9), 193 (13), 177 (35), 137 (53), 102 (100).

##### *N*-{4-[(5-Chloro-4-oxo-4*H*-1,2,6-thiadiazin-3-yl)thio]phenyl}acetamide (**11d**)

To a stirred mixture of 3,5-dichloro-4*H*-1,2,6-thiadiazin-4-one (**2**) (92.0 mg, 0.50 mmol) in THF (2 mL) at ca. 20 °C, *N*-(4-mercaptophenyl)acetamide was added (84.0 mg, 0.50 mmol), followed by Et_3_N (69 *μ*L, 0.50 mmol), and the mixture was stirred at this temperature until complete consumption of the starting material (TLC, 1 h). The mixture was then adsorbed onto silica and chromatographed (DCM/*t*-BuOMe 50:50) to give the title compound **11d** (127 mg, 81%) as yellow needles, mp 191–192 °C (from EtOH); R*_f_* 0.62 (DCM/*t*-BuOMe 50:50); (found: C, 42.14; H, 2.49; N, 13.32. C_11_H_8_ClN_3_O_2_S_2_ requires C, 42.11; H, 2.57; N, 13.39%); *λ*_max_ (DCM)/nm 278 (log *ε* 4.22), 294 (4.25), 358 (4.08), 416 (3.76); *v*_max_/cm^−1^ 3319w (N-H), 1670m and 1649s (C=O), 1589m, 1518m, 1485m, 1458w, 1398m, 1368m, 1354m, 1294m, 1271m, 1261m, 1244m, 1180m, 1061m, 829m, 746s, 721m, 716m; *δ*_H_ (300 MHz; DMSO-*d*_6_) 10.19 (1H, s, N*H*), 7.70 (2H, d, *J* 8.7, Ar C*H*), 7.43 (2H, d, *J* 8.7, Ar C*H*), 2.07 (3H, s, C*H*_3_); *δ*_C_ (75 MHz; DMSO-*d*_6_) 168.7 (*C*q), 163.5 (*C*q), 159.2 (*C*q), 143.5 (*C*q), 141.0 (*C*q), 135.9 (*C*H), 119.8 (*C*H), 119.2 (*C*q), 24.0 (*C*H_3_); *m*/*z* (APCI^+^) 316 (MH^+^+2, 42%), 314 (MH^+^, 100).

##### 3-Chloro-5-[(1-phenyl-1*H*-tetrazol-5-yl)thio]-4*H*-1,2,6-thiadiazin-4-one (**11e**)

To a stirred mixture of 3,5-dichloro-4*H*-1,2,6-thiadiazin-4-one (**2**) (92.0 mg, 0.50 mmol) in THF (2 mL) at *ca*. 20 °C, 1-phenyl-1*H*-tetrazole-5-thiol was added (63.0 mg, 0.50 mmol), followed by Et_3_N (69 *μ*L, 0.50 mmol), and the mixture was stirred at this temperature until complete consumption of the starting material (TLC, 5 h). The mixture was then adsorbed onto silica and chromatographed (*n*-hexane/DCM 20:80) to give the title compound **11e** (64 mg, 39%) as colorless plates, mp 182–183 °C (from EtOH); R*_f_* 0.16 (*n*-hexane/DCM 20:80); (found: C, 37.06; H, 1.40; N, 25.54. C_10_H_5_ClN_6_OS_2_ requires C, 36.98; H, 1.55; N, 25.88%); *λ*_max_ (MeCN)/nm 235 inf (log *ε* 4.19), 305 (4.26), 347 (4.30); *v*_max_/cm^−1^ 3100w and 3063w (C-H arom), 1655s (C=O), 1497m, 1485m, 1418w, 1406m, 1279m, 1244w, 1219m, 1123w, 1067m, 1015w, 760s, 745s, 727m; *δ*_H_ (300 MHz; CDCl_3_) 7.60–7.51 (5H, m, Ar C*H*); *δ*_C_ (125 MHz; CDCl_3_) 159.0 (*C*q), 158.1 (*C*q), 146.2 (*C*q), 145.3 (*C*q), 133.2 (*C*q), 131.1 (*C*H), 129.9 (*C*H), 124.6 (*C*H); *m*/*z* (MALDI-TOF) 327 (MH^+^+2, 31%), 325 (MH^+^, 82), 279 (54), 220 (46), 128 (100), 100 (49).

##### 3-Chloro-5-[(4-methyl-4*H*-1,2,4-triazol-3-yl)thio]-4*H*-1,2,6-thiadiazin-4-one (**11f**)

To a stirred mixture of 3,5-dichloro-4*H*-1,2,6-thiadiazin-4-one (**2**) (92.0 mg, 0.50 mmol) in THF (2 mL) at *ca*. 20 °C, 4-methyl-4*H*-1,2,4-triazole-3-thiol was added (58.0 mg, 0.50 mmol), followed by Et_3_N (69 *μ*L, 0.50 mmol), and the mixture was stirred at this temperature until complete consumption of the starting material (TLC, 3 h). The precipitate was then filtered, washed with EtOH (5 mL) and dried to give the title compound **11f** (106 mg, 81%) as yellow needles, mp 152–153 °C (from THF/EtOH); R*_f_* 0.18 (DCM/*t*-BuOMe 50:50); (found: C, 27.69; H, 1.46; N, 26.74. C_6_H_4_ClN_5_OS_2_ requires C, 27.54; H, 1.54; N, 26.76%); *λ*_max_ (DCM)/nm 263 (log *ε* 4.60), 291 (4.60), 346 (4.11); *v*_max_/cm^−1^ 2959w, 2920w and 2853w (C-H), 1624s (C=O), 1506m, 1470w, 1418w, 1290m, 1254w, 1192m, 1163w, 1065m, 1016w, 957w, 856m, 748s, 733m; *δ*_H_ (500 MHz; DMSO-*d*_6_) 8.85 (1H, s, Ar C*H*), 3.60 (3H, s, C*H*_3_); *δ*_C_ (125 MHz; DMSO-*d*_6_) 159.7 (*C*q), 159.3 (*C*q), 148.2 (*C*H), 144.3 (*C*q), 141.8 (*C*q), 31.3 (*C*H_3_); *m*/*z* (MALDI-TOF) 264 (MH^+^+2, 25%), 262 (MH^+^, 65), 184 (23), 100 (100).

##### 3-Chloro-5-(thiazol-2-ylthio)-4*H*-1,2,6-thiadiazin-4-one (**11g**)

To a stirred mixture of 3,5-dichloro-4*H*-1,2,6-thiadiazin-4-one (**2**) (92.0 mg, 0.50 mmol) in THF (2 mL) at *ca*. 20 °C, thiazole-2-thiol was added (59.0 mg, 0.50 mmol), followed by Et_3_N (69 *μ*L, 0.50 mmol), and the mixture was stirred at this temperature until complete consumption of the starting material (TLC, 1 h). The mixture was then adsorbed onto silica and chromatographed (*n*-hexane/DCM 20:80) to give the title compound **11g** (96 mg, 73%) as yellow needles, mp 136–137 °C (from EtOH); R*_f_* 0.29 (*n*-hexane/DCM 20:80); (found: C, 27.17; H, 0.54; N, 15.79. C_6_H_2_ClN_3_OS_3_ requires C, 27.33; H, 0.76; N, 15.93%); *λ*_max_ (DCM)/nm 247 (log *ε* 3.94), 308 (4.16), 352 (4.08); *v*_max_/cm^−1^ 3053w (C-H arom), 1643s (C=O), 1605w, 1567w, 1491m, 1468m, 1350m, 1312m, 1279m, 1260w, 1242w, 1159w, 1063s, 1034s, 741s, 719s; *δ*_H_ (300 MHz; CDCl_3_) 8.04 (1H, d, *J* 3.4, Ar C*H*), 7.68 (1H, d, *J* 3.3, Ar C*H*); *δ*_C_ (125 MHz; DMSO-*d*_6_) 160.1 (*C*q), 159.0 (*C*q), 152.4 (*C*q), 144.2 (*C*q), 144.1 (*C*H), 126.8 (*C*H); *m*/*z* (MALDI-TOF) 266 (MH^+^+2, 68%), 264 (MH^+^, 100), 247 (90), 232 (42), 142 (29).

##### 3-Chloro-5-[(6-ethoxybenzo[*d*]thiazol-2-yl)thio]-4*H*-1,2,6-thiadiazin-4-one (**11i**)

To a stirred mixture of 3,5-dichloro-4*H*-1,2,6-thiadiazin-4-one (**2**) (92.0 mg, 0.50 mmol) in THF (2 mL) at *ca*. 20 °C, 6-ethoxybenzo[*d*]thiazole-2-thiol was added (106 mg, 0.50 mmol), followed by Et_3_N (69 *μ*L, 0.50 mmol), and the mixture was stirred at this temperature until complete consumption of the starting material (TLC, 2 h). The mixture was then adsorbed onto silica and chromatographed (*n*-hexane/DCM 20:80) to give the title compound **11i** (77 mg, 43%) as yellow needles, mp 180–181 °C (from DCE/EtOH); R*_f_* 0.32 (*n*-hexane/DCM 20:80); (found: C, 40.19; H, 2.55; N, 11.48. C_12_H_8_ClN_3_O_2_S_3_ requires C, 40.28; H, 2.25; N, 11.74%); *λ*_max_ (MeCN)/nm 213 (log *ε* 4.44), 222 inf (4.41), 256 (3.99), 314 (4.32), 346 inf (4.14); *v*_max_/cm^−1^ 3088w and 3061w (C-H arom), 2994w, 2943w and 2891w (C-H alip), 1634s (C=O), 1595m, 1558w, 1541w, 1481m, 1460m, 1431m, 1396m, 1321w, 1273m, 1260s, 1229s, 1130w, 1107w, 1066m, 1036m, 1016m, 939m, 851m, 827m, 746s, 725m; *δ*_H_ (300 MHz; DMSO-*d*_6_) 7.96 (1H, d, *J* 8.9, Ar C*H*), 7.74 (1H, d, *J* 2.5, Ar C*H*), 7.17 (1H, ddd, *J* 9.0, 2.5, Ar C*H*), 4.11 (2H, q, *J* 7.0, OC*H*_2_), 1.37 (3H, t, *J* 7.0, C*H*_3_); *δ*_C_ (75 MHz; DMSO-*d*_6_) 159.7 (*C*q), 158.9 (*C*q), 157.2 (*C*q), 152.3 (*C*q), 146.5 (*C*q), 144.3 (*C*q), 138.8 (*C*H), 123.6 (*C*H), 116.7 (*C*H), 105.0 (*C*H), 63.8 (*C*H_2_), 14.5 (*C*H_3_); *m*/*z* (MALDI-TOF) 360 (MH^+^+2, 24%), 358 (MH_+_, 56), 280 (100), 233 (29), 210 (40), 182 (30).

##### 2-{3-Chloro-5-[(4-methylpyrimidin-2-yl)thio]-4*H*-1,2,6-thiadiazin-4-ylidene}malononitrile (**12a**)

To a stirred mixture of 2-(3,5-dichloro-4*H*-1,2,6-thiadiazin-4-ylidene)malononitrile (**3**) (116 mg, 0.50 mmol) in DCM (5 mL) at *ca*. 0 °C, 4-methylpyrimidine-2-thiol was added (63.0 mg, 0.50 mmol), followed by Hünig’s base (87 *μ*L, 0.50 mmol), and the mixture was left to warm to *ca*. 20 °C and stirred until complete consumption of the starting material (TLC, 30 min). The mixture was then adsorbed onto silica and chromatographed (*n*-hexane/DCM 20:80) to give the title compound **12a** (114 mg, 71%) as yellow needles, mp 126–127 °C (from *c*-hexane); R*_f_* 0.86 (*n*-hexane/DCM 20:80); (found: C, 41.36; H, 1.43; N, 26.17. C_11_H_5_ClN_6_S_2_ requires C, 41.19; H, 1.57; N, 26.20%); *λ*_max_ (DCM)/nm 238 (log *ε* 4.28), 271 inf (4.09), 353 (4.06), 424 (4.28); *v*_max_/cm^−1^ 2226m (C≡N), 1574s, 1537s, 1504w, 1487m, 1454m, 1435m, 1418m, 1373w, 1344m, 1337s, 1333s, 1285s, 1204m, 1182m, 1157m, 1105m, 1096m, 1078w, 943w, 883m, 851m, 810m, 795m, 785s, 764m, 754s, 716s; *δ*_H_ (500 MHz; CDCl_3_) 8.43 (1H, d, *J* 5.0, Ar C*H*), 7.04 (1H, d, *J* 5.0, Ar C*H*), 2.50 (3H, s, C*H*_3_); *δ*_C_ (125 MHz; CDCl_3_) 163.3 (*C*q), 166.6 (*C*q), 157.6 (*C*H), 144.7 (*C*q), 141.5 (*C*q), 137.0 (*C*q), 118.7 (*C*H), 111.9 (*C*q), 111.8 (*C*q), 79.2 (*C*q), 24.2 (*C*H_3_); *m*/*z* (APCI^+^) 323 (MH^+^+2, 21%), 321 (MH^+^, 60), 313 (100), 285 (M^+^-Cl,), 267 (44), 239 (62), 197 (C_6_ClN_4_S^+^+2, 34), 195 (C_6_ClN_4_S^+^, 100), 159 (51).

##### 2-{3-Chloro-5-[(1-methyl-1*H*-tetrazol-5-yl)thio]-4*H*-1,2,6-thiadiazin-4-ylidene}malononitrile (**12b**)

To a stirred mixture of 2-(3,5-dichloro-4*H*-1,2,6-thiadiazin-4-ylidene)malononitrile (**3**) (116 mg, 0.50 mmol) in DCM (5 mL) at *ca*. 0 °C, 1-methyl-1*H*-tetrazole-5-thiol was added (58.0 mg, 0.50 mmol), followed by Hünig’s base (87 *μ*L, 0.50 mmol), and the mixture was left to warm to *ca*. 20 °C and stirred until complete consumption of the starting material (TLC, 3.5 h). The precipitate formed was then filtered, washed with *n*-hexane (5 mL), and dried to give the title compound **12b** (64 mg, 41%). The filtrate was then adsorbed onto silica and chromatographed (DCM/*t*-BuOMe 80:20) to give a further quantity of the title compound **12b** (23 mg, total yield 56%) as orange needles, mp >300 °C (from DCE/EtOH); R*_f_* 0.48 (DCM/*t*-BuOMe 80:20); (found: C, 31.06; H, 0.84; N, 36.00. C_8_H_3_ClN_8_S_2_ requires C, 30.92; H, 0.97; N, 36.06%); *λ*_max_ (DCM)/nm 240 (log *ε* 3.99), 314 (3.96), 324 inf (3.94), 359 (3.84), 438 (4.05); *v*_max_/cm^−1^ 2911w (C-H), 2201w (C≡N), 1624s, 1493m, 1470m, 1449m, 1402m, 1377w, 1310m, 1271w, 1207m, 1179m, 1148w, 1069s, 1026w, 986w, 930w, 853w, 831w, 739s, 725s; *δ*_H_ (500 MHz; CD_3_CN) 4.06 (3H, s, C*H*_3_); *δ*_C_ (125 MHz; CD_3_CN) 146.7 (*C*q), 145.1 (*C*q), 137.7 (*C*q), 114.3 (*C*q), 35.6 (*C*H_3_), three *C*q resonances missing; *m*/*z* (APCI^+^) 313 (MH^+^+2, 19%), 311 (MH^+^, 20), 273 (41), 265 (41), 263 (97), 199 (33), 185 (100).

##### 2-[3-(Benzo[*d*]oxazol-2-ylthio)-5-chloro-4*H*-1,2,6-thiadiazin-4-ylidene]malononitrile (**12c**)

To a stirred mixture of 2-(3,5-dichloro-4*H*-1,2,6-thiadiazin-4-ylidene)malononitrile (**3**) (116 mg, 0.50 mmol) in DCM (5 mL) at *ca*. 0 °C, benzo[*d*]oxazole-2-thiol was added (76.0 mg, 0.50 mmol), followed by Hünig’s base (87 *μ*L, 0.50 mmol), and the mixture was left to warm to *ca*. 20 °C and stirred until complete consumption of the starting material (TLC, 3 h). The mixture was then adsorbed onto silica and chromatographed (DCM) to give the title compound **12c** (85 mg, 49%) as orange plates, mp 192–193 °C (from *c*-hexane); R*_f_* 0.65 (DCM); (found: C, 45.38; H, 1.04; N, 20.16. C_13_H_13_ClN_4_OS requires C, 45.16; H, 1.17; N, 20.25%); *λ*_max_ (DCM)/nm 251 (log *ε* 4.33), 276 (4.22), 284 (4.22), 425 (4.35); *v*_max_/cm^−1^ 3150w (C-H arom), 2208m (C≡N), 1493m, 1470m, 1445m, 1339w, 1288m, 1265w, 1242w, 1213w, 1146m, 1125m, 1098m, 1076m, 1003w, 961w, 937w, 845w, 818m, 806m, 744s; *δ*_H_ (500 MHz; CDCl_3_) 7.78 (1H, dd, *J* 7.3, 1.6, Ar C*H*), 7.59 (1H, dd, *J* 7.3, 1.1, Ar C*H*), 7.47–7.41 (2H, m, Ar C*H*); *δ*_C_ (125 MHz; CDCl_3_) 153.7 (*C*q), 152.7 (*C*q), 146.1 (*C*q), 141.4 (*C*q), 137.3 (*C*q), 136.3 (*C*q), 126.8 (*C*H), 125.4 (*C*H), 120.6 (*C*H), 112.3 (*C*q), 111.7 (*C*q), 111.0 (*C*H), 79.6 (*C*q); *m*/*z* (MALDI-TOF) 348 (MH^+^+2, 40%), 346 (MH^+^, 100), 287 (21), 151 (26).

### 3.3. Biological In Vitro Assays

For the in vitro studies, the synthesized compounds were prepared as a 10 mM stock solution in DMSO. To determine the IC_50_ values, serial dilutions were performed. Each assay was performed six times, and the standard deviations were less than 10% of the average.

#### 3.3.1. Determination of the Reducing Activity of the Stable Radical DPPH

In vitro assays were carried out to evaluate the reducing activity of the novel derivatives against the DPPH stable radical. Free radical scavenging activity was determined by measuring absorbance at 517 nm after 20 and 60 min at room temperature. NDGA served as the reference compound. (Table 4) [44].

#### 3.3.2. Inhibition of AAPH-Induced Linoleic Acid Peroxidation

AAPH was used as an alkyl peroxyl radical inducer. In this experiment, the compounds’ ability to prevent linoleic acid oxidation by alkyl peroxyl radicals through absorbance changes at 234nm was evaluated, corresponding to the production of 13-hydroperoxy-linoleic acid. Trolox was used as the reference compound. The results are presented in Table 4 [44].

#### 3.3.3. Inhibition of Soybean Lipoxygenase

Sodium linoleate (0.1 mM), 0.2 mL of soybean lipoxygenase solution (1/9 × 10^−4^ *w*/*v* in saline), and 10 μL of the test compound stock solution (10 mM in DMSO) were incubated at room temperature. The formation of 13-hydroperoxy-linoleic acid was monitored by measuring absorbance at 234 nm. NDGA was used as the reference compound. A couple of concentrations of the test compounds were used to calculate the IC_50_ values. The results are presented in Table 4 [44].

### 3.4. Computational Methods

#### 3.4.1. Molecular Docking Studies on Soybean Lipoxygenase

The protein structure (PDB ID: 3PZW) was visualized and preprocessed using UCSF Chimera (version 1.18) [45]. Chimera was used to remove water molecules and non-essential crystallographic compounds. Missing residues (Met1–Phe2–Ser3–Ala4–Gly5; Glu21–Val22–Asn23–Pro24–Asp25–Gly26–Ser27–Ala28–Val28–Asp29; Ile117–Ser118–Asn119–Gln120) were added using Modeller (v. 10.3) [46]. Hydrogen atoms and partial charges were incorporated using AmberTools 23 [47,48]. A +2.0 charge was assigned to the iron center using the 12-6 Lennard-Jones (LJ) non-bonded model. [49]. Histidine residues (His499, His504, His690), which coordinate the iron, were modeled as neutral with δ-nitrogen protonation. For solvation, the simulated box kept a minimum of 12 Å between the solute and the box boundary using the TIP3P water model. Ligand 3D structures were generated and minimized using Open Babel (v. 3.1.1) [50] with the MMFF94 force field [51]. Ligand topologies and parameters were generated with ACPYPE [52], employing AnteChamber (AmberTools v. 22.10) [53]. Energy minimization of the protein was performed using GROMACS (v. 4.6.5). [54]. Ligand docking was carried out with AutoDock Vina (v. 1.2.3) [55], using a grid box centered at x = 1.35 Å, y = 14.3 Å, z = −34.60 Å, and with dimensions x = 100 Å, y = 70 Å, z = 70 Å. The exhaustiveness was set to 10, with up to 20 docking modes generated. Docking outcomes were examined using UCSF Chimera.

#### 3.4.2. In Silico Determination of Drug-likeness and Lipophilicity

The compounds’ drugability was performed using the online software Molinspiration (https://www.molinspiration.com/, accessed on 9 September).

Lipophilicity was theoretically calculated as clog *p* values by the Bio-Loom program of BioByte Corp (http://biobyte.com/bb/prod/bioloom.html, accessed on 9 June 2025) [56].

## 4. Conclusions

Fifteen novel 1,2,6-thiadiazine derivatives were synthesized via mono-displacement of dichlorothiadiazinone **2** and dicyanoylidene **3** with amine and thiol nucleophiles. All the compounds were evaluated for antioxidant activity (DPPH radical scavenging and AAPH-induced lipid peroxidation) and anti-inflammatory potential (soybean lipoxygenase inhibition). Structural characterization was confirmed by melting point and spectroscopic analysis (IR, ^1^H/^13^C NMR, MS).

Compounds **9a**,**b**, **10a**,**b**, and **11a**–**i** showed low, time-independent DPPH activity, whereas compounds **12a**–**c** exhibited markedly enhanced scavenging, with **12c** and **12a** reaching 92% and 90% interaction at 60 min, respectively. The superior activity of **12a**–**c** compared to **11a**–**c** indicates that a 4-dicyanoylidine group in the thiadiazine, instead of a ketone, is more favorable for DPPH interaction. Similarly, AAPH inhibition was low in the **9a**,**b** and **10a**,**b** series and moderate to low in **11a**–**i**, while **12c** demonstrated the strongest effect (70%, at 100 µM), further supporting the beneficial role of the 4-dicyanoylidine group.

sLOX inhibition was generally moderate to low, except for compound **9a** (IC_50_ = 7.5 µM), which bears ketone and phenyl substitutions. Overall, compound **12c** emerged as the most potent antioxidant across both DPPH and AAPH assays, while **9a** showed the most significant lipoxygenase inhibitory activity. These results highlight the influence of specific substitutions on the biological activity of 1,2,6-thiadiazine derivatives and provide guidance for further optimization.

## Figures and Tables

**Table 3:** Drug-likeness studies of synthesized compounds.

Compd	Milog P ^a^	TPSA ^b^	No of Atoms	No of O and N ^c^	No of OH and NH ^d^	No of Violations	No of Rotational Bonds ^e^	Volume ^f^	MW ^g^	Clog *P* ^h^
**9a**	2.7	49.33	20	5	0	0	2	252.30	308.79	2.12
**9b**	4.18	49.33	27	5	0	0	4	340.54	398.92	4.21
**10a**	3.05	79.84	24	6	0	0	2	294.46	356.84	1.69
**10b**	4.53	79.84	31	6	0	0	4	382.69	446.97	3.75
**11a**	1.92	68.64	16	5	0	0	2	196.75	272.74	1.32
**11b**	1.01	86.47	15	7	0	0	2	177.96	262.71	0.66
**11c**	3.22	68.89	18	5	0	0	2	209.90	297.75	2.54
**11d**	2.36	71.95	19	5	1	0	3	236.45	313.79	1.87
**11e**	2.28	86.47	20	7	0	0	3	232.81	324.78	1.81
**11f**	1.15	73.57	15	6	0	0	2	182.11	261.72	0.34
**11g**	2.36	55.75	14	4	0	0	2	175.05	263.76	1.61
**11h**	3.86	55.75	18	4	0	0	2	219.05	313.82	3.21
**11i**	4.27	64.98	21	5	0	0	4	261.39	357.87	4.03
**12a**	2.27	99.15	20	6	0	0	2	238.90	320.79	0.63
**12b**	1.35	116.98	19	8	0	0	2	220.11	310.75	0.88
**12c**	3.57	99.4	22	6	0	0	2	252.05	345.80	0.6

^a^ Logarithm of partition coefficient between *n*-octanol and water (milog P); ^b^ Topological polar surface area (TPSA); ^c^ number of hydrogen bond acceptors; ^d^ number of hydrogen bond donors; ^e^ number of rotatable bonds; ^f^ molecular volume; ^g^ molecular weight; ^h^ theoretically calculated lipophilicity.

**Table 4:** Interaction with DPPH at 20 and 60 min, inhibition of AAPH-induced linoleic acid peroxidation (AAPH%), and in vitro inhibitory activity against soybean lipoxygenase (expressed as % inhibition and IC_50_) were evaluated for the synthesized compounds.

Compound	RA%, DPPH, 20 min (100 µM)	RA%, DPPH, 60 min (100 µM)	LOX % Inhibition 100 μM or IC_50_ (μM)	%AAPH (100 µM)
**9a**	7	6	7.5 μM	15
**9b**	2	2	55 μM	20
**10a**	16	15	46%	27
**10b**	no	no	67.5 μM	No
**11a**	2	no	31%	No
**11b**	3	3	44%	45
**11c**	21	22	77.5 μM	5
**11d**	1	2	85 μM	43
**11e**	15	17	50%	No
**11f**	10	12	52.5 μM	7
**11g**	10	7	40%	No
**11h**	8	1	33%	No
**11i**	3	no	67.5 μM	No
**12a**	82	90	41%	18
**12b**	27	36	75 μM	No
**12c**	89	92	27%	70
**ΝDGA**	87	93	93% 0.45 μM (93%)	-
**Trolox**	-	-	-	93

no: no action under the experimental conditions. Standard deviation (±SD) of the mean is less than <10%. For the in vitro assays, the mean value is derived from at least six independent experiments.

## Data Availability

The original contributions presented in this study are included in the article/Supplementary Materials. Further inquiries can be directed to the corresponding authors.

## Supplementary Materials

The following supporting information can be downloaded at https://www.mdpi.com/article/10.3390/ijms262411817/s1.

## Abbreviations

The following abbreviations are used in this manuscript:

AAPH	2,2′-Azobis(2-amidinopropane) dihydrochloride
ADMET	Absorption, Distribution, Metabolism, Excretion, Toxicity
DCM	Dichloromethane
DPPH	2,2-diphenyl-1-picrylhydrazyl
EtOH	Ethanol
GPX	Glutathione peroxidase
MW	Molecular weight
NDGA	Nordihydroguaiaretic acid
OS	Oxidative stress
PIDA	(Diacetoxyiodo)benzene
ROS	Reactive oxygen species
SOD	Superoxide dismutase
sLOX	Soybean lipoxygenase
THF	Tetrahydrofuran
TLC	Thin-layer chromatography
TPSA	Topological polar surface area
Uv/vis	Ultraviolet/visible light

## References

[1] Sibony R.W., Segev O., Dor S., Raz I. (2024). Overview of oxidative stress and inflammation in diabetes. J. Diabetes.

[2] Arshad N., Jawaid S., Hashim J., Ullah I., Gul S., Aziz A., Wadood A., Khan A. (2023). Highly potent anti-inflammatory, analgesic and antioxidant activities of 3,5-disubstituted tetrahydro-2*H*-1,3,5-thiadiazine thiones. Bioorg. Med. Chem. Lett..

[3] Soták M., Clark M., Suur B.E., Börgeson E. (2025). Inflammation and resolution in obesity. Nat. Rev. Endocrinol..

[4] Chen A., Huang H., Fang S., Hang Q. (2024). ROS: A “booster” for chronic inflammation and tumor metastasis. BBA Rev. Cancer.

[5] Chopra D., Shukla S., Rana P., Kamar M.D., Gaur P., Bala M., Pathaniya D., Tripathi A., Dwivedi A., Gupta S., Poojan S. (2024). Overview of Inflammation. Inflammation Resolution and Chronic Diseases.

[6] Jin S., Kang P.M. (2024). A Systematic Review on Advances in Management of Oxidative Stress-Associated Cardiovascular Diseases. Antioxidants.

[7] Oguntibeju O.O. (2019). Type 2 diabetes mellitus, oxidative stress and inflammation: Examining the links. Int. J. Physiol. Pathophysiol. Pharmacol..

[8] Yu Y., Liu S., Yang L., Song P., Liu Z., Liu X., Yan X., Dong Q. (2024). Roles of reactive oxygen species in inflammation and cancer. MedComm.

[9] Perillo B., Di Donato M., Pezone A., Di Zazzo E., Giovannelli P., Galasso G., Castoria G., Migliaccio A. (2020). ROS in cancer therapy: The bright side of the moon. Exp. Mol. Med..

[10] Weintraup P.M., Katritzky A.R., Ramsden C.A., Scriven E.F.V., Taylor R.J.K. (2008). 1,2,6-Oxadiazines and 1,2,6-Thiadiazines. Comprehensive Heterocyclic Chemistry III.

[11] Asquith C.R.M., Godoi P.H., Couñago R.M., Laitinen T., Scott J.W., Langendorf C.G., Oakhill J.S., Drewry D.H., Zuercher W.J., Koutentis P.A. (2018). 1,2,6-Thiadiazinones as novel narrow spectrum calcium/calmodulin-dependent protein kinase kinase 2 (CaMKK2) inhibitors. Molecules.

[12] Peake C.J., Harnish W.N., Davidson B.L. (1978). Mono-5-Substituted-3-Chloro-4H-1,2,6-Thiadiazin-4-One Antifungal Agents. U.S. Patent.

[13] Peake C.J., Harnish W.N., Davidson B.L. (1978). Mono-5-Substituted-Thio-3-Chloro-4H-1,2,6-Thiadiazin-4-One Antifungal Agents. U.S. Patent.

[14] Peake C.J., Harnish W.N., Davidson B.L. (1979). 3-Chloro-5-(Optionally Substituted Heterocycloxy)-4H-1,2,6-Thiadiazin-4-one Antifungal Agents. U.S. Patent.

[15] Peake C.J., Harnish W.N., Davidson B.L. (1980). Mono-5-Substituted-3-Chloro-4H-1,2,6-Thiadiazin-4-One Antifungal Agents. U.S. Patent.

[16] Portnoy R.C. (1985). Thiadiazinone Plant Disease Control Agents. U.S. Patent.

[17] Gómez T., Macho S., Miguel D., Neo A.G., Rodríguez T., Torroba T. (2005). Cyclopentathiadiazines, cyclohepta- and cyclopen-tadithiazoles: New materials and a rich heterocyclic chemistry of cyclic enaminonitriles. Eur. J. Org. Chem..

[18] Macho S., Miguel D., Neo A.G., Rodríguez T., Torroba T. (2005). Cyclopentathiadiazines, new heterocyclic materials from cyclic enaminonitriles. Chem. Commun..

[19] Hermerschmidt F., Kalogirou A.S., Min J., Zissimou G.A., Tuladhar S.M., Ameri T., Faber H., Itskos G., Choulis S.A., Anthopoulos T.D. (2015). 4*H*-1,2,6-Thiadiazin-4-one-containing small molecule donors and additive effects on their performance in solu-tion-processed organic solar cells. J. Mater. Chem. C.

[20] Haddon R.C., Kaplan M.L., Marshall J.H. (1978). Naphtho[1,8-*cd*:4,5-*c’d*’]bis[1,2,6]thiadiazine. A compound of ambiguous aromatic character. J. Am. Chem. Soc..

[21] Cava M., Lakshmikantham M.V., Hoffmann R., Williams R.M.R.B. (2011). Woodward’s unfinished symphony: Designing organic superconductors (1975–79). Tetrahedron.

[22] Lonchakov A.V., Rakitin O.A., Gritsan N.P., Zibarev A.V. (2013). Breathing Some New Life into an Old Topic: Chalcogen-Nitrogen π-Heterocycles as Electron Acceptors. Molecules.

[23] Kalogirou A.S., Black D.S., Cossy J., Stevens C.V. (2022). 1,2,6-Oxadiazines and 1,2,6-Thiadiazines. Comprehensive Heterocyclic Chemistry IV.

[24] Kalogirou A.S., Kourtellaris A., Koutentis P.A. (2022). Oxidations of 4*H*-1,2,6-thiadiazines. ChemistrySelect.

[25] Broumidis E., Thomson C.G., Gallagher B., Sotorríos L., McKendrick K.G., Macgregor S.A., Paterson M.J., Lovett J.E., Lloyd G.O., Rosair G.M. (2023). The photochemical mediated ring contraction of 4*H*-1,2,6-thiadiazines to afford 1,2,5-thiadiazol-3(2*H*)-one 1-oxides. Org. Lett..

[26] Rodríguez H., Suárez M., Albericio F. (2012). Thiadiazines, N,N-Heterocycles of Biological Relevance. Molecules.

[27] Čačić M., Pavić V., Molnar M., Šarkanj B., Has-Schön E. (2014). Design and Synthesis of Some New 1,3,4-Thiadiazines with Coumarin Moieties and Their Antioxidative and Antifungal Activity. Molecules.

[28] de Araújo A.C.J., Freitas P.R., Araújo I.M., de Oliveira Borges J.A., Gonçalves S.A., Paulo C.L.R., Almeida R.S., de Moraes Oliveira-Tintino C.D., de Araújo-Neto J.B., dos Santos Nascimento I.J. (2024). Assessment In vitro and In silico of the Activity of Thiadiazines as NorA Efflux Pump Inhibitors. Curr. Microbiol..

[29] Vitaku E., Smith D.T., Njardarson J.T. (2014). Analysis of the structural diversity, substitution patterns, and frequency of nitrogen heterocycles among U.S. FDA approved pharmaceuticals. J. Med. Chem..

[30] Romanelli M.N., Manetti D., Braconi L., Dei S., Gabellini A., Teodori E. (2022). The piperazine scaffold for novel drug discovery efforts: The evidence to date. Expert. Opin. Drug Discov..

[31] Kalogirou A.S., Koutentis P.A. (2022). Reactions of 4*H*-1,2,6-thiadiazine sulfides. Molbank.

[32] Bate-Smith E.C., Westall R.G. (1950). Chromatographic behaviour and chemical structure I. Some naturally occuring phenolic substances. Biochim. Biophys. Acta.

[33] Waring M.J. (2010). Lipophilicity in drug discovery. Expert. Opin. Drug Discov..

[34] Heim K.E., Tagliaferro A.R., Bobilya D.J. (2002). Flavonoid antioxidants: Chemistry, metabolism and structure-activity relationships. J. Nutr. Biochem..

[35] Bariamis S.E., Magoulas G.E., Grafanaki K., Pontiki E., Tsegenidis T., Athanassopoulos C.M., Maroulis G., Papaioannou D., Hadjipavlou-Litina D. (2015). Synthesis and biological evaluation of new C-10 substituted dithranol pleiotropic hybrids. Bioorg. Med. Chem..

[36] Pontiki E., Hadjipavlou-Litina D. (2018). Multi-Target Cinnamic Acids for Oxidative Stress and Inflammation: Design, Synthesis, Biological Evaluation and Modeling Studies. Molecules.

[37] Rådmark O., Werz O., Steinhilber D., Samuelsson B. (2015). 5-Lipoxygenase, a key enzyme for leukotriene biosynthesis in health and disease. BBA Mol. Cell Biol. Lipids.

[38] Maccarrone M., Melino G., Finazzi-Agro A. (2001). Lipoxygenases and their involvement in programmed cell death. Cell Death Differ..

[39] Dixon R.A., Jones R.E., Diehl R.E., Bennett C.D., Kargman S., Rouzer C.A. (1988). Cloning of the cDNA for human 5-lipoxygenase. PNAS.

[40] Kostopoulou I., Tzani A., Polyzos N.-I., Karadendrou M.-A., Kritsi E., Pontiki E., Liargkova T., Hadjipavlou-Litina D., Zoumpoulakis P., Detsi A. (2021). Exploring the 2′-Hydroxy-Chalcone Framework for the Development of Dual Antioxidant and Soybean Lipoxygenase Inhibitory Agents. Molecules.

[41] El Khatabi K., El-Mernissi R., Aanouz I., Ajana M.A., Lakhlifi T., Khan A., Wei D.-Q., Bouachrine M. (2021). Identification of novel acetylcholinesterase inhibitors through 3D-QSAR, molecular docking, and molecular dynamics simulation targeting Alzheimer’s disease. J. Mol. Model..

[42] Geevers J., Trompen W.P. (1974). Synthesis and reactions of 3,5-dichloro-4*H*-1,2,6-thiadiazin-4-one. Recl. Trav. Chim. Pays-Bas.

[43] Kalogirou A.S., Koutentis P.A. (2015). A qualitative comparison of the reactivities of 3,4,4,5-tetrachloro-4*H*-1,2,6-thiadiazine and 4,5-dichloro-1,2,3-dithiazolium chloride. Molecules.

[44] Kouzi O., Pontiki E., Hadjipavlou-Litina D. (2019). 2-Arylidene-1-indandiones as Pleiotropic Agents with Antioxidant and Inhibitory Enzymes Activities. Molecules.

[45] Pettersen E.F., Goddard T.D., Huang C.C., Couch G.S., Greenblatt D.M., Meng E.C., Ferrin T.E. (2004). UCSF Chimera—A visualization system for exploratory research and analysis. J. Comput. Chem..

[46] Fiser A., Sali A. (2003). Modeller: Generation and refinement of homology-based protein structure models. Methods Enzymol..

[47] Lindorff-Larsen K., Piana S., Palmo K., Maragakis P., Klepeis J.L., Dror R.O., Shaw D.E. (2010). Improved side-chain torsion potentials for the Amber ff99SB protein force field. Proteins.

[48] Case D.A., Aktulga H.M., Belfon K., Cerutti D.S., Cisneros G.A., Cruzeiro V.W.D., Forouzesh N., Giese T.J., Götz A.W., Gohlke H. (2023). AmberTools. J. Chem. Inf. Model..

[49] Li P., Roberts B.P., Chakravorty D.K., Merz K.M. (2013). Rational Design of Particle Mesh Ewald Compatible Lennard-Jones Parameters for +2 Metal Cations in Explicit Solvent. J. Chem. Theory Comput..

[50] O’Boyle N.M., Banck M., James C.A., Morley C., Vandermeersch T., Hutchison G.R. (2011). Open Babel: An open chemical toolbox. J. Cheminform..

[51] Halgren T.A. (1996). Merck molecular force field. I. Basis, form, scope, parameterization, and performance of MMFF94. J. Comput. Chem..

[52] Sousa da Silva A.W., Vranken W.F. (2012). ACPYPE—AnteChamber PYthon Parser interfacE. BMC Res. Notes.

[53] Wang J., Wang W., Kollman P.A., Case D.A. (2006). Automatic atom type and bond type perception in molecular mechanical calculations. J. Mol. Graph. Model..

[54] Hess B., Kutzner C., van der Spoel D., Lindahl E. (2008). GROMACS 4: Algorithms for Highly Efficient, Load-Balanced, and Scalable Molecular Simulation. J. Chem. Theory. Comput..

[55] Trott O., Olson A.J. (2010). AutoDock Vina: Improving the speed and accuracy of docking with a new scoring function, efficient optimization, and multithreading. J. Comput. Chem..

[56] (2002). Chem-Bio Informatics and Comparative QSAR. http://biobyte.com/bb/prod/bioloom.html.

