# Supplementary material for: Novel Mono-Substituted 4H-1,2,6-Thiadiazines with Antioxidant and Anti-Lipoxygenase Activities
Source: Int J Mol Sci. 2025 Dec 7;26(24):11817. doi: 10.3390/ijms262411817 (PMC12733323; doi:10.3390/ijms262411817)
Supplement: Supplementary file 1 [file ijms-26-11817-s001.zip › ijms-3949258-supplementary.pdf]

Supporting Information

Novel mono-substituted 4*H*-1,2,6-thiadiazines with antioxidant and anti-lipoxygenase activities

Eleftherios Charissopoulos, Panayiotis A. Koutentis, Andreas S. Kalogirou,* and Eleni Pontiki*

Contents **Page**

S1. Proposed mechanisms for the transformations of 3,4,4,5-tetrachloro-4*H*-1,2,6-thiadiazine (**1**) to 3,5-dichloro-4*H*-1,2,6-thiadiazin-4-one (**2**) and the photochemical oxidation of 4*H*-1,2,6-thiadiazines **7** to 1,2,5-thiadiazole 1-oxides **8**. S3

S2. References S4

S3. ^{1}H and ^{13}C NMR spectra of novel 1,2,6-thiadiazines S5-S35

S1. Proposed mechanisms for the transformations of 3,4,4,5-tetrachloro-4*H*-1,2,6-thiadiazine (1) to 3,5-dichloro-4*H*-1,2,6-thiadiazin-4-one (2) and the photochemical oxidation of 4*H*-1,2,6-thiadiazines 7 to 1,2,5-thiadiazole 1-oxides 8.

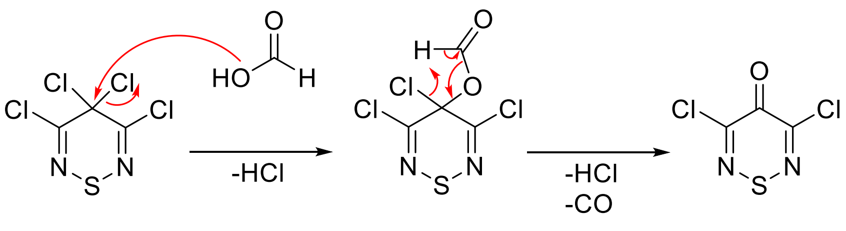

1 **2**

Figure S1. Proposed mechanism for the transformation of 3,4,4,5-tetrachloro-4*H*-1,2,6-thiadiazine (**1**) to 3,5-dichloro-4*H*-1,2,6-thiadiazin-4-one (**2**) [1].

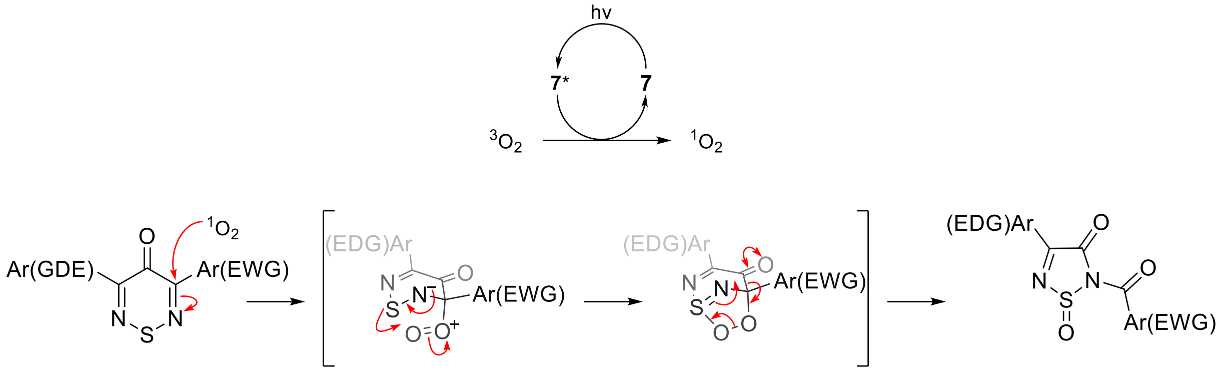

7 **8**

Figure S2. Proposed mechanism for the photochemical oxidation of 4*H*-1,2,6-thiadiazines **7** to 1,2,5-thiadiazole 1-oxides **8** [2].

S2. References

1. Geevers, J.; Trompen, W.P. Synthesis and reactions of 3,5-dichloro-4*H*-1,2,6-thiadiazin-4-one. *Recl. Trav. Chim. Pays-Bas* **1974**, *93*, 270-272. Doi: 10.1002/recl.19740930911

2. Broumidis, E.; Thomson, C.G.; Gallagher, B.; Sotorríos, L.; McKendrick, K.G.; Macgregor, S.A.; Paterson, M.J.; Lovett, J.E.; Lloyd, G.O.; Rosair, G.M.; Kalogirou, A.S.; Koutentis, P.A.; Vilela, F. The photochemical mediated ring contraction of 4*H*-1,2,6-thiadiazines to afford 1,2,5-thiadiazol-3(2*H*)-one 1-oxides. *Org. Lett.* **2023**, *25*, 6907-6912. Doi: 10.1021/acs.orglett.3c02673

S3. ^{1}H and ^{13}C NMR spectra of novel 1,2,6-thiadiazines

^{1}H NMR of 3-chloro-5-(4-phenylpiperazin-1-yl)-4*H*-1,2,6-thiadiazin-4-one (**9a**)

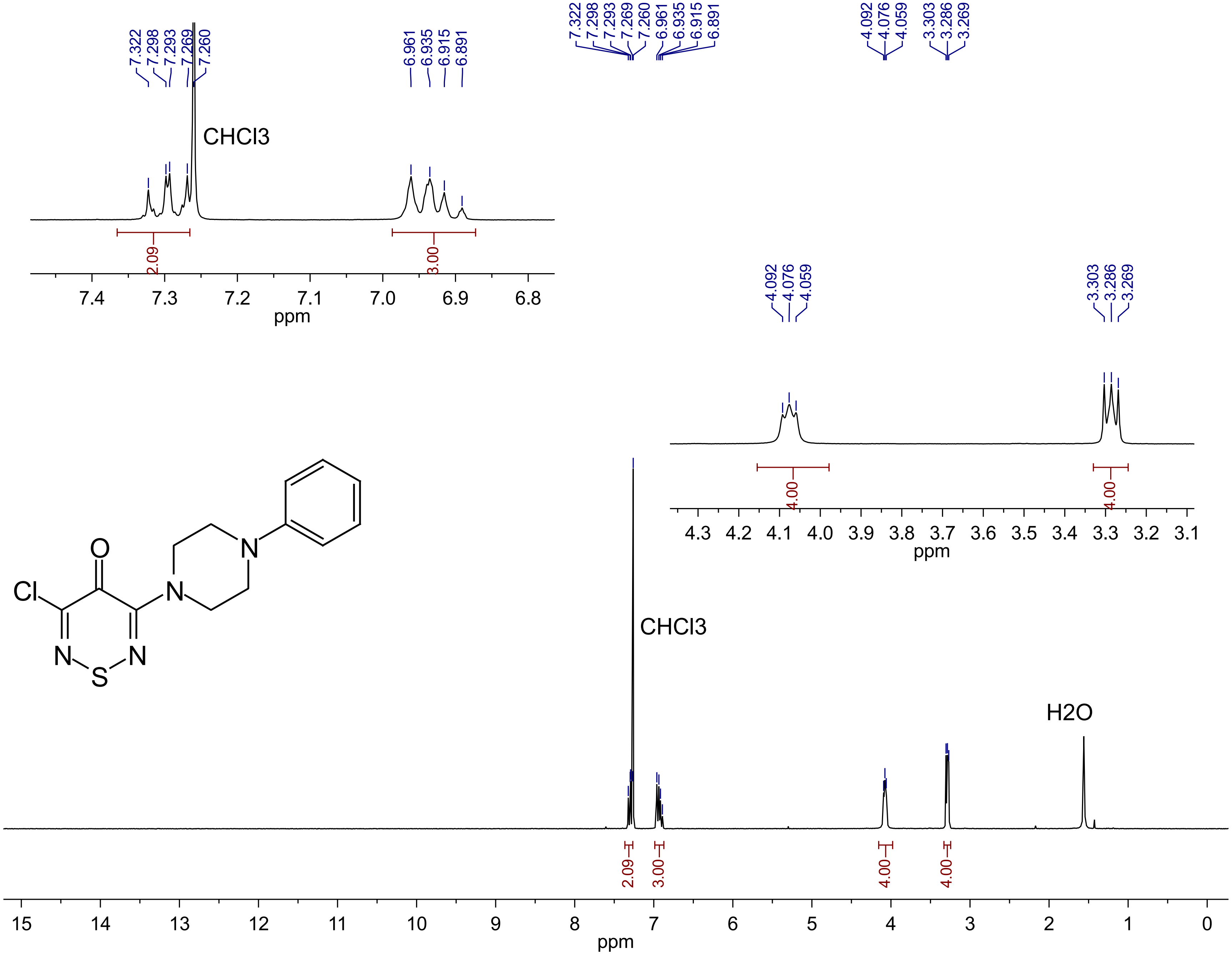

Current Data Parameters
NAME Andreas
EXPNO 175
PROCNO 1
F2 - Acquisition Parameters
Date_ 20190731
Time 19.18 h
INSTRUM spect
PROBHD Z104275_0375 (
PULPROG zg30
TD 65536
SOLVENT CDCl3
NS 16
DS 2
SWH 6009.615 Hz
FIDRES 0.183399 Hz
AQ 5.4525952 sec
RG 201.81
DW 83.200 usec
DE 6.50 usec
TE 297.6 K
D1 1.00000000 sec
TD0 1
SFO1 300.1318533 MHz
NUC1 1H
P1 14.00 usec
PLW1 6.69999981 W

F2 - Processing parameters
SI 65536
SF 300.1300072 MHz
WDW EM
SSB 0
LB 0.30 Hz
GB 0
PC 1.00

^{13}C NMR of 3-chloro-5-(4-phenylpiperazin-1-yl)-4*H*-1,2,6-thiadiazin-4-one (**9a**)

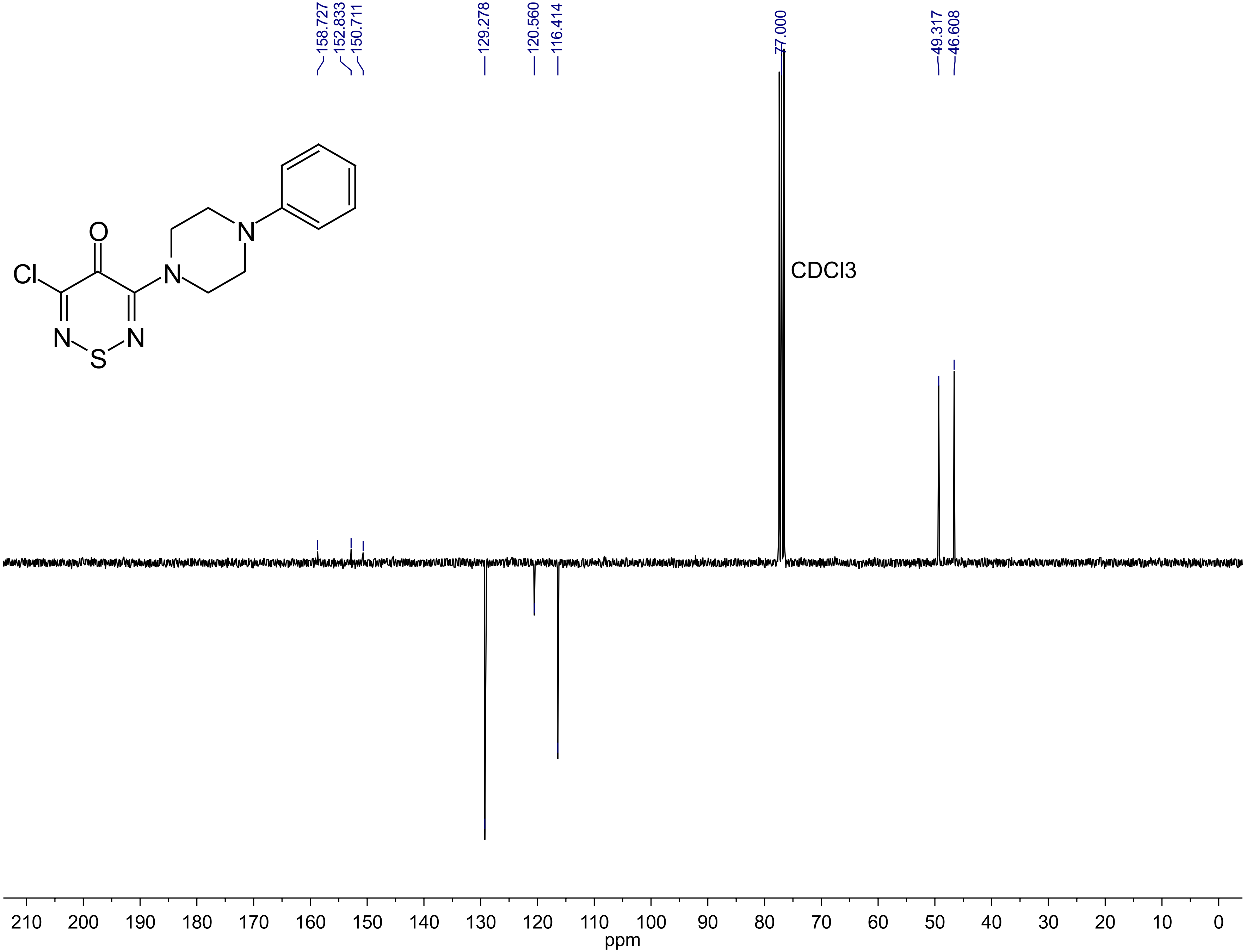

Current Data Parameters
NAME Andreas
EXPNO 176
PROCNO 1
F2 - Acquisition Parameters
Date_ 20190801
Time 7.00 h
INSTRUM spect
PROBHD Z104275_0375 (
PULPROG jmod
TD 65536
SOLVENT CDCl3
NS 10824
DS 4
SWH 18115.941 Hz
FIDRES 0.552855 Hz
AQ 1.8087935 sec
RG 201.81
DW 27.600 usec
DE 6.50 usec
TE 297.9 K
CNST2 145.0000000
CNST11 1.0000000
D1 2.00000000 sec
D20 0.00689655 sec
TD0 1
SFO1 75.4752953 MHz
NUC1 13C
P1 10.00 usec
P2 20.00 usec
PLW1 41.00000000 W
SFO2 300.1312005 MHz
NUC2 1H
CPDPRG[2 waltz16
PCPD2 90.00 usec
PLW2 6.69999981 W
PLW12 0.16212000 W
F2 - Processing parameters
SI 32768
SF 75.4677496 MHz
WDW EM
SSB 0
LB 1.00 Hz
GB 0
PC 1.40

^{1}H NMR of 3-(4-Benzhydrylpiperazin-1-yl)-5-chloro-4*H*-1,2,6-thiadiazin-4-one (**9b**)

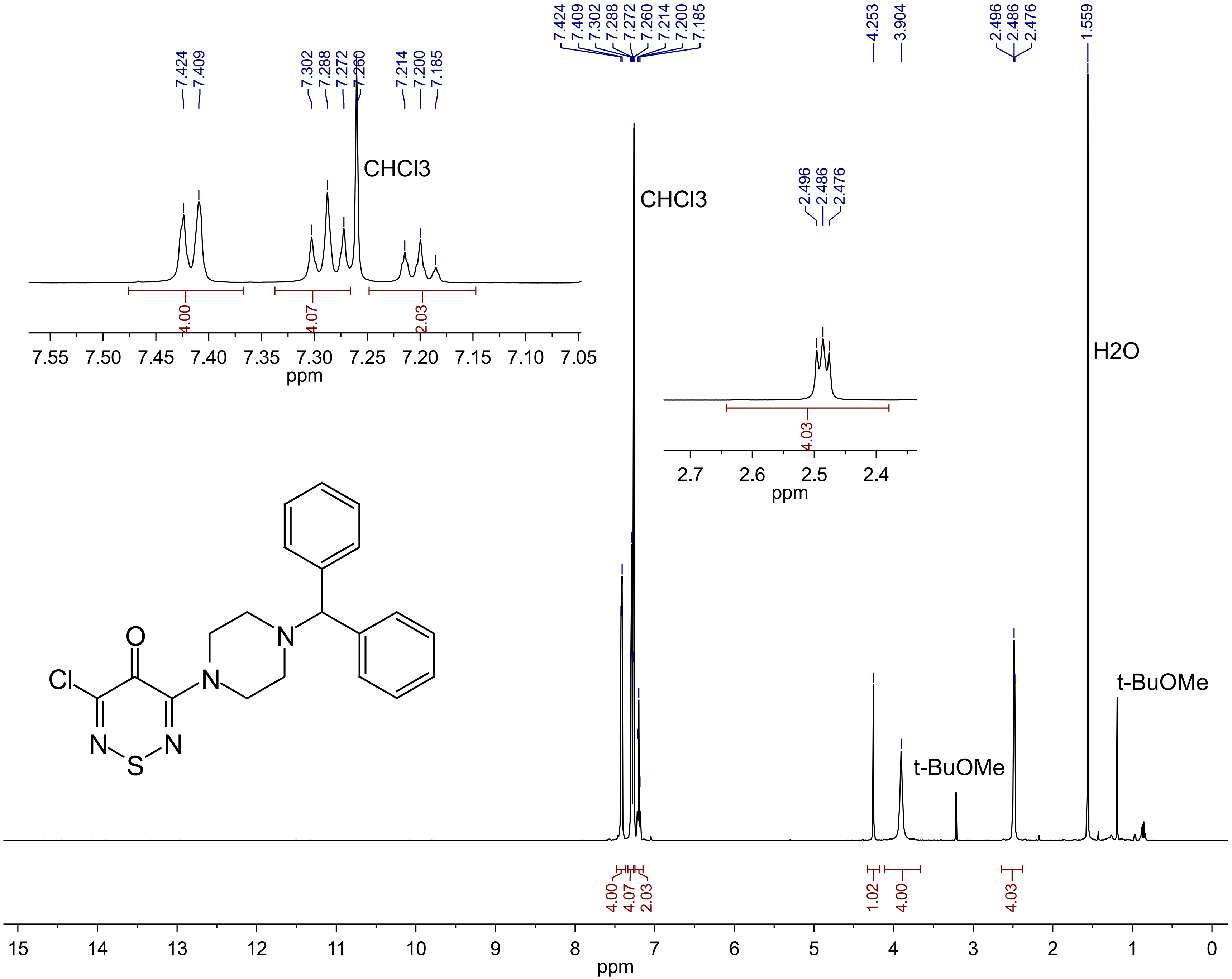

Current Data Parameters
NAME Kalogirou
EXPNO 642
PROCNO 1

F2 - Acquisition Parameters
Date_ 20190816
Time 11.00
INSTRUM spect
PROBHD 5 mm PABBO BB-
PULPROG zg30
TD 65536
SOLVENT CDCl3
NS 16
DS 2
SWH 10000.000 Hz
FIDRES 0.152588 Hz
AQ 3.2767999 sec
RG 161
DW 50.000 usec
DE 6.50 usec
TE 296.9 K
D1 1.00000000 sec
TD0 1

======== CHANNEL f1 ========
SFO1 500.0361158 MHz
NUC1 1H
P1 12.00 usec
PLW1 14.50000000 W

F2 - Processing parameters
SI 65536
SF 500.0330401 MHz
WDW EM
SSB 0
LB 0.30 Hz
GB 0
PC 1.00

^{13}C NMR of 3-(4-Benzhydrylpiperazin-1-yl)-5-chloro-4*H*-1,2,6-thiadiazin-4-one (**9b**)

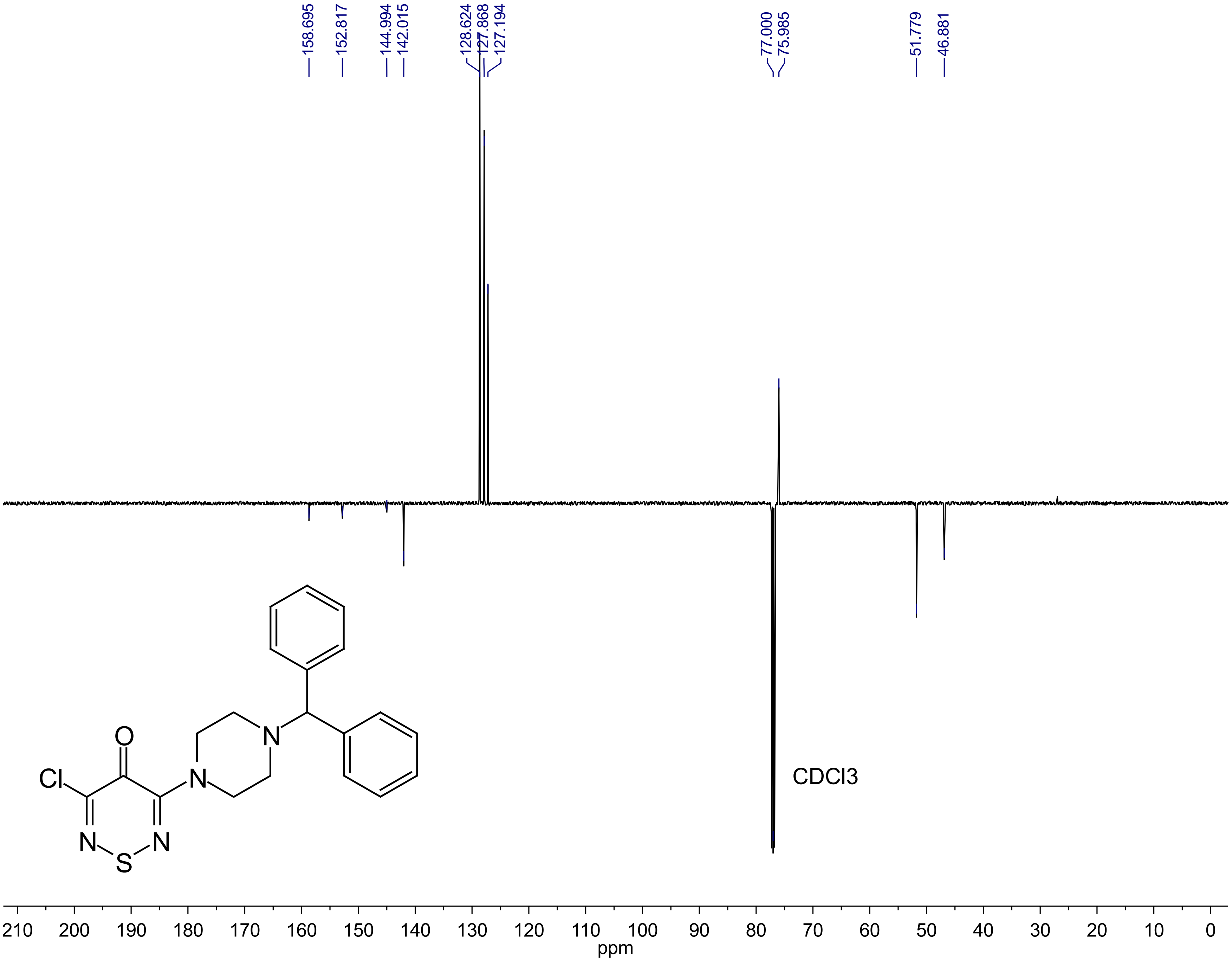

Current Data Parameters
NAME Kalogirou
EXPNO 643
PROCNO 1
F2 - Acquisition Parameters
Date_ 20190816
Time 14.02
INSTRUM spect
PROBHD 5 mm PABBO BB-
PULPROG jmod
TD 65536
SOLVENT CDCl3
NS 10000
DS 4
SWH 29761.904 Hz
FIDRES 0.454131 Hz
AQ 1.1010048 sec
RG 2050
DW 16.800 usec
DE 6.50 usec
TE 297.5 K
CNST2 145.0000000
CNST11 1.0000000
D1 2.00000000 sec
D20 0.00689655 sec
TD0 1
======== CHANNEL f1 ========
SFO1 125.7459782 MHz
NUC1 13C
P1 9.20 usec
P2 18.40 usec
PLW1 140.00000000 W
======== CHANNEL f2 ========
SFO2 500.0350280 MHz
NUC2 1H
CPDPRG[2 waltz16
PCPD2 80.00 usec
PLW2 14.50000000 W
PLW12 0.32624999 W
F2 - Processing parameters
SI 32768
SF 125.7334081 MHz
WDW EM
SSB 0
LB 1.00 Hz
GB 0
PC 1.40

^{1}H NMR of 2-[3-Chloro-5-(4-phenylpiperazin-1-yl)-4*H*-1,2,6-thiadiazin-4-ylidene]malononitrile (**10a**)

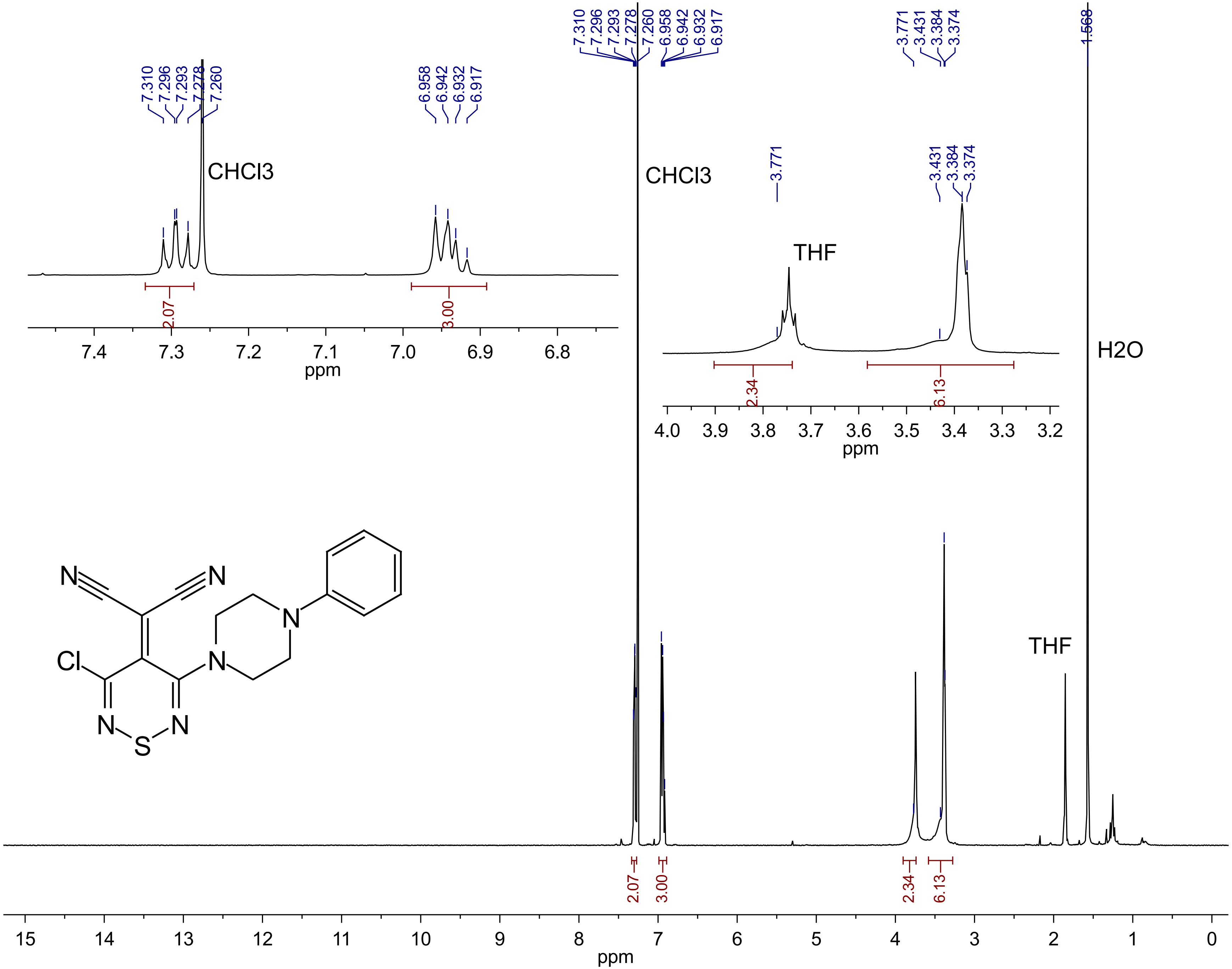

Current Data Parameters
NAME Kalogirou
EXPNO 640
PROCNO 1

F2 - Acquisition Parameters
Date_ 20190814
Time 13.43
INSTRUM spect
PROBHD 5 mm PABBO BB-
PULPROG zg30
TD 65536
SOLVENT CDCl3
NS 16
DS 2
SWH 10000.000 Hz
FIDRES 0.152588 Hz
AQ 3.2767999 sec
RG 161
DW 50.000 usec
DE 6.50 usec
TE 296.3 K
D1 1.00000000 sec
TD0 1

CHANNEL f1
SFO1 500.0361158 MHz
NUC1 1H
P1 12.00 usec
PLW1 14.50000000 W

F2 - Processing parameters
SI 65536
SF 500.0330401 MHz
WDW EM
SSB 0
LB 0.30 Hz
GB 0
PC 1.00

^{13}C NMR of 2-[3-Chloro-5-(4-phenylpiperazin-1-yl)-4*H*-1,2,6-thiadiazin-4-ylidene]malononitrile (**10a**)

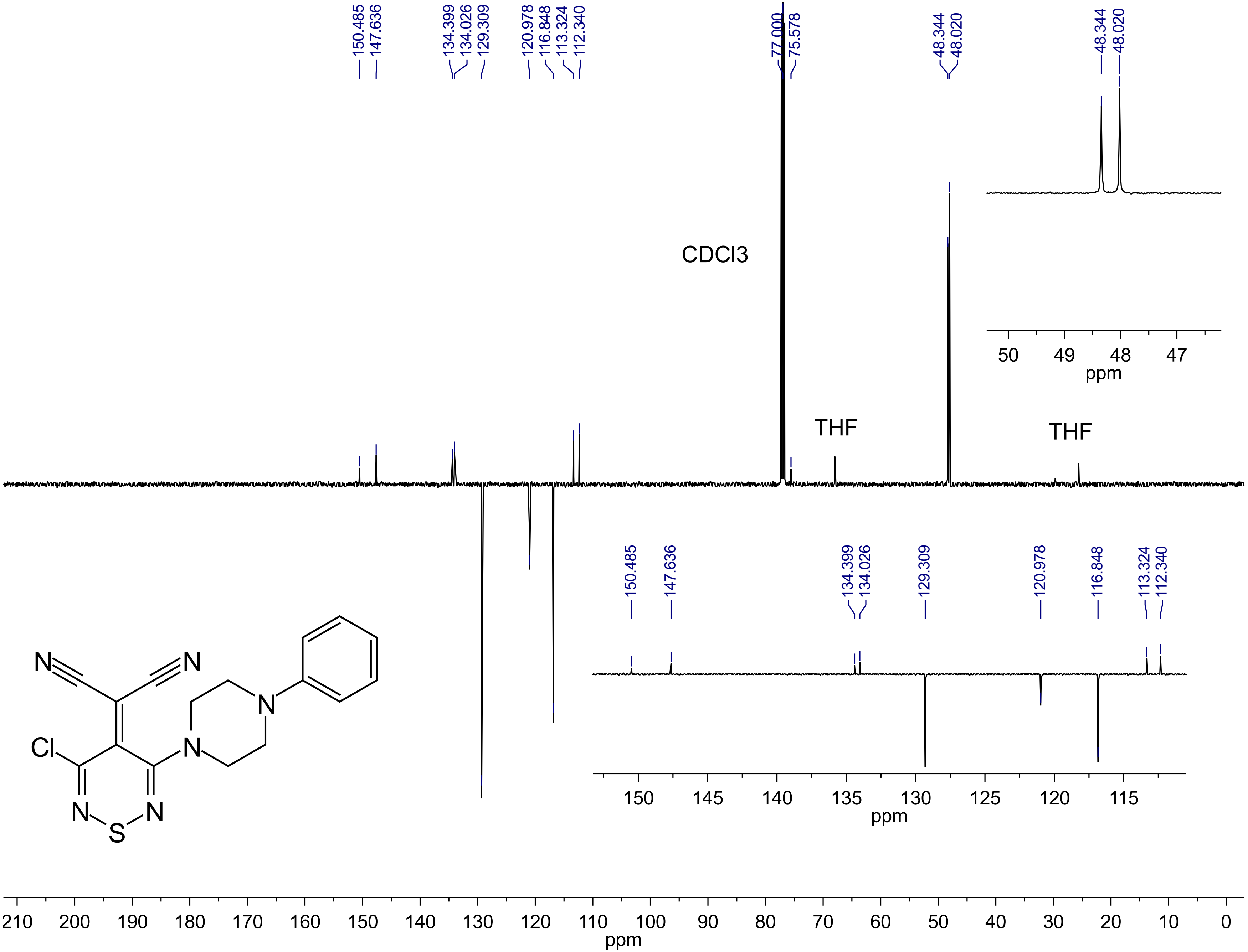

Current Data Parameters
NAME Kalogirou
EXPNO 641
PROCNO 1
F2 - Acquisition Parameters
Date_ 20190814
Time 13.51
INSTRUM spect
PROBHD 5 mm PABBO BB-
PULPROG jmod
TD 65536
SOLVENT CDCl3
NS 10000
DS 4
SWH 29761.904 Hz
FIDRES 0.454131 Hz
AQ 1.1010048 sec
RG 2050
DW 16.800 usec
DE 6.50 usec
TE 297.2 K
CNST2 145.0000000
CNST11 1.0000000
D1 2.00000000 sec
D20 0.00689655 sec
TD0 1
CHANNEL f1
SFO1 125.7459782 MHz
NUC1 13C
P1 9.20 usec
P2 18.40 usec
PLW1 140.00000000 W
CHANNEL f2
SFO2 500.0350280 MHz
NUC2 1H
CPDPRG[2 waltz16
PCPD2 80.00 usec
PLW2 14.50000000 W
PLW12 0.32624999 W
F2 - Processing parameters
SI 32768
SF 125.7334082 MHz
WDW EM
SSB 0
LB 1.00 Hz
GB 0
PC 1.40

^{1}H NMR of 2-[3-(4-Benzhydrylpiperazin-1-yl)-5-chloro-4*H*-1,2,6-thiadiazin-4-ylidene]malononitrile (**10b**)

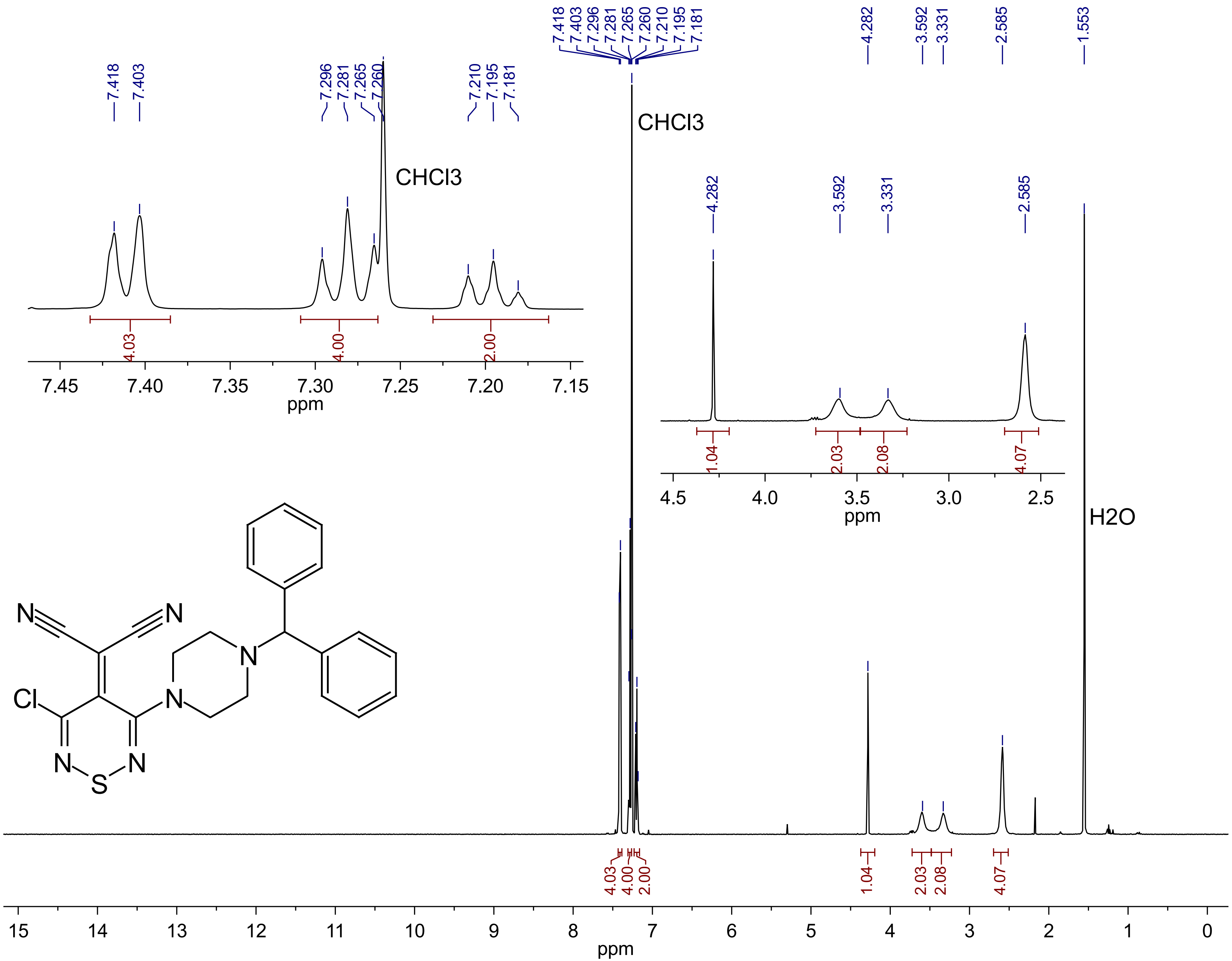

Current Data Parameters
NAME Kalogirou
EXPNO 659
PROCNO 1

F2 - Acquisition Parameters
Date_ 20190824
Time 9.13
INSTRUM spect
PROBHD 5 mm PABBO BB-
PULPROG zg30
TD 65536
SOLVENT CDCl3
NS 16
DS 2
SWH 10000.000 Hz
FIDRES 0.152588 Hz
AQ 3.2767999 sec
RG 181
DW 50.000 usec
DE 6.50 usec
TE 298.5 K
D1 1.00000000 sec
TD0 1

======== CHANNEL f1 ========
SFO1 500.0361158 MHz
NUC1 1H
P1 12.00 usec
PLW1 14.50000000 W

F2 - Processing parameters
SI 65536
SF 500.0330402 MHz
WDW EM
SSB 0
LB 0.30 Hz
GB 0
PC 1.00

^{13}C NMR of 2-[3-(4-Benzhydrylpiperazin-1-yl)-5-chloro-4*H*-1,2,6-thiadiazin-4-ylidene]malononitrile (**10b**)

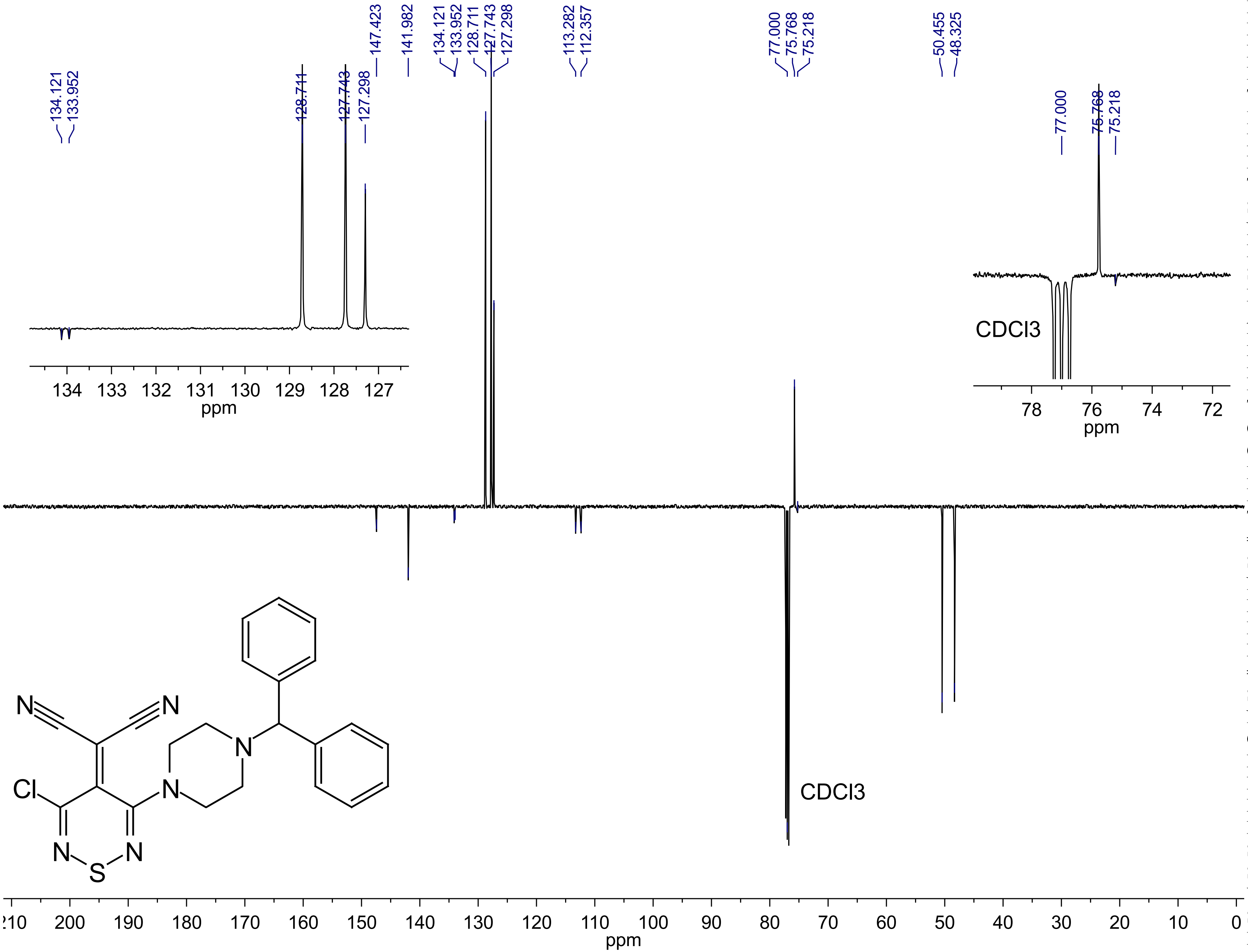

Current Data Parameters
NAME Kalogirou
EXPNO 660
PROCNO 1
F2 - Acquisition Parameters
Date_ 20190824
Time 9.10
INSTRUM spect
PROBHD 5 mm PABBO BB-
PULPROG jmod
TD 65536
SOLVENT CDCl3
NS 15000
DS 4
SWH 29761.904 Hz
FIDRES 0.454131 Hz
AQ 1.1010048 sec
RG 2050
DW 16.800 usec
DE 6.50 usec
TE 299.4 K
CNST2 145.0000000
CNST11 1.0000000
D1 2.00000000 sec
D20 0.00689655 sec
TD0 1
======== CHANNEL f1 ========
SFO1 125.7459782 MHz
NUC1 13C
P1 9.20 usec
P2 18.40 usec
PLW1 140.00000000 W
======== CHANNEL f2 ========
SFO2 500.0350280 MHz
NUC2 1H
CPDPRG[2 waltz16
PCPD2 80.00 usec
PLW2 14.50000000 W
PLW12 0.32624999 W
F2 - Processing parameters
SI 32768
SF 125.7334070 MHz
WDW EM
SSB 0
LB 1.00 Hz
GB 0
PC 1.40

^{1}H NMR of 3-chloro-5-[(4-methylpyrimidin-2-yl)thio]-4*H*-1,2,6-thiadiazin-4-one (**11a**)

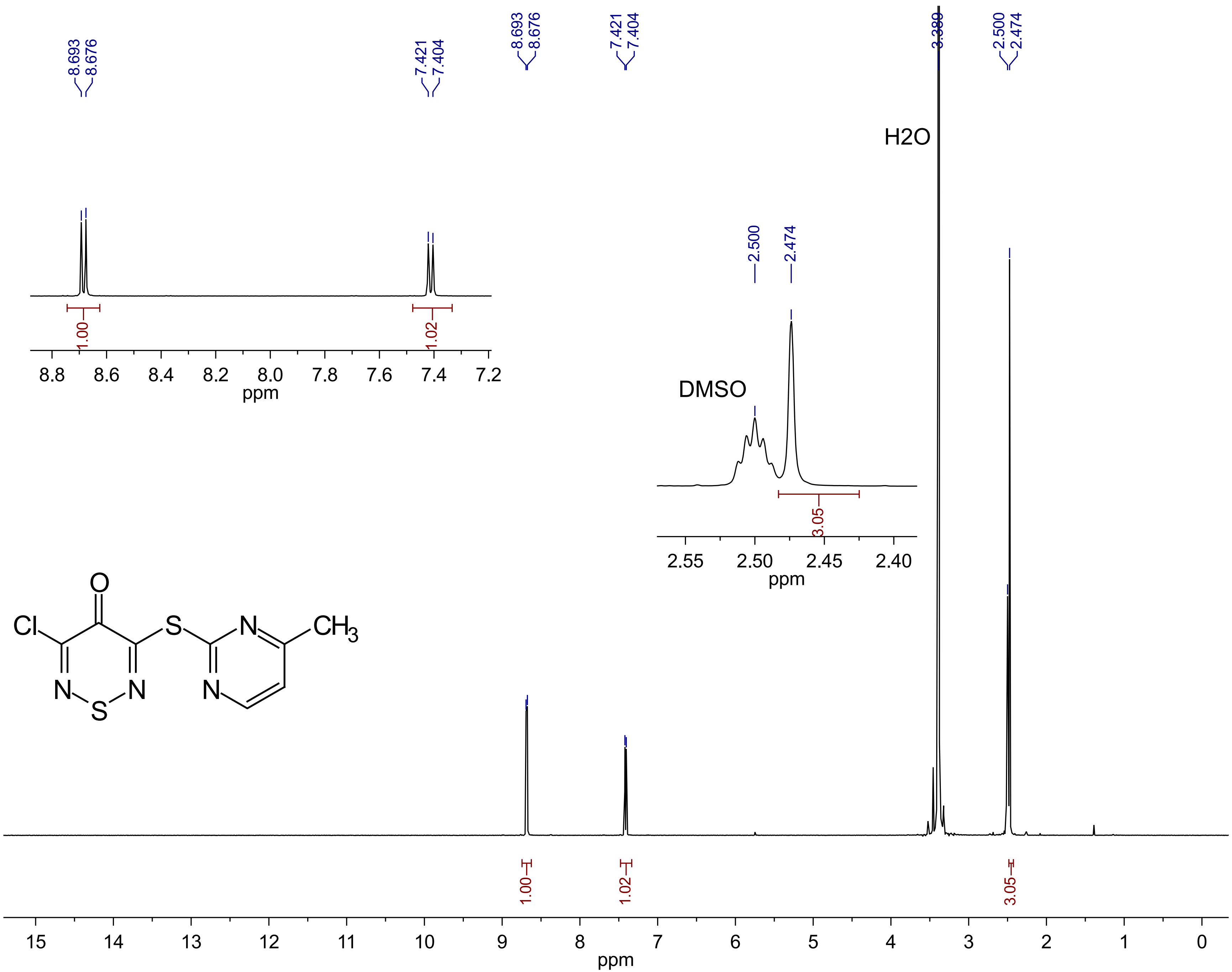

Current Data Parameters
NAME Andreas
EXPNO 205
PROCNO 1

F2 - Acquisition Parameters
Date_ 20191023
Time 18.03 h
INSTRUM spect
PROBHD Z104275_0375 (
PULPROG zg30
TD 65536
SOLVENT DMSO
NS 16
DS 2
SWH 6009.615 Hz
FIDRES 0.183399 Hz
AQ 5.4525952 sec
RG 201.81
DW 83.200 usec
DE 6.50 usec
TE 294.3 K
D1 1.00000000 sec
TD0 1
SFO1 300.1318533 MHz
NUC1 1H
P1 14.00 usec
PLW1 6.69999981 W

F2 - Processing parameters
SI 65536
SF 300.1300029 MHz
WDW EM
SSB 0
LB 0.30 Hz
GB 0
PC 1.00

^{13}C NMR of 3-chloro-5-[(4-methylpyrimidin-2-yl)thio]-4*H*-1,2,6-thiadiazin-4-one (**11a**)

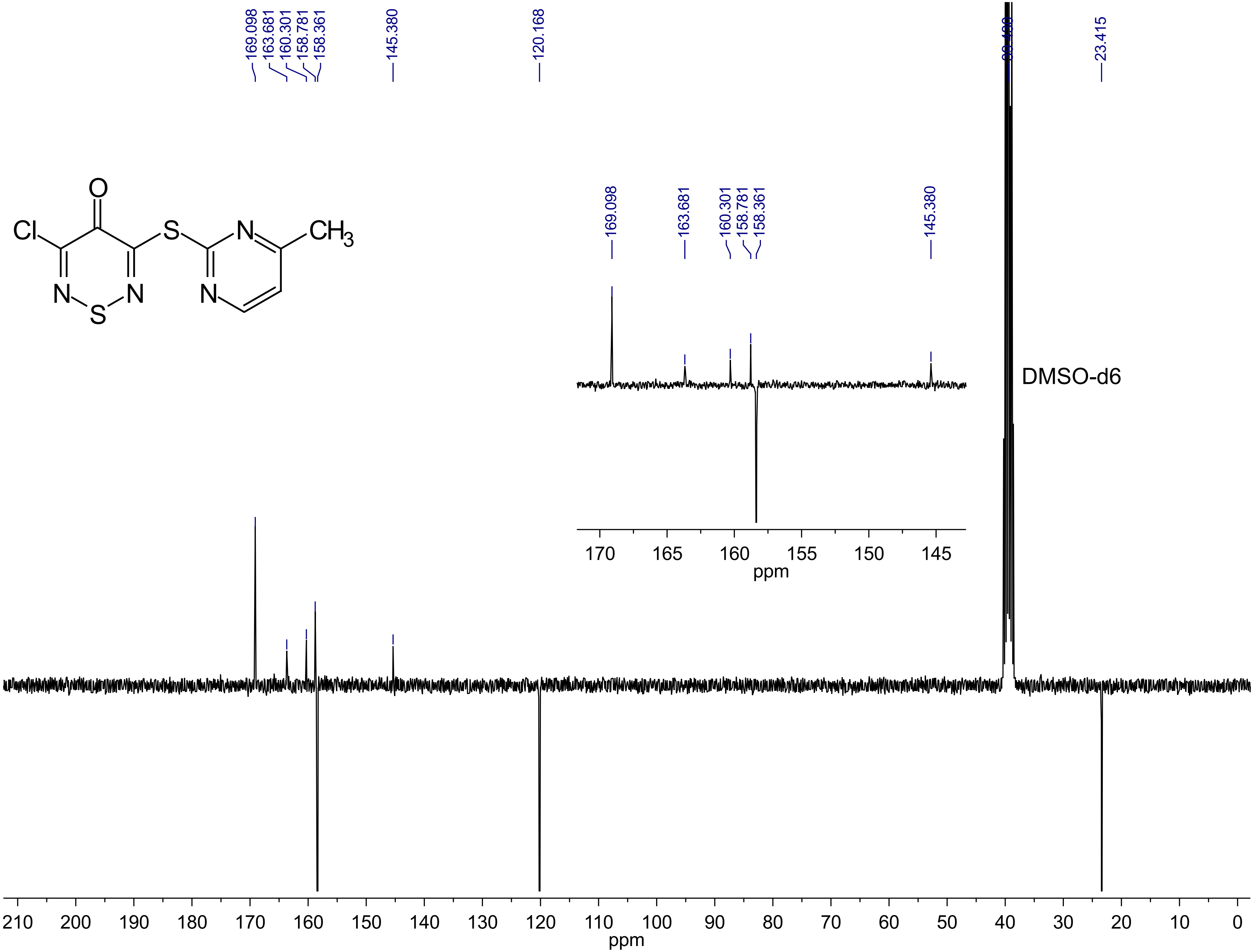

Current Data Parameters
NAME Andreas
EXPNO 206
PROCNO 1
F2 - Acquisition Parameters
Date_ 20191024
Time 10.14 h
INSTRUM spect
PROBHD Z104275_0375 (
PULPROG jmod
TD 65536
SOLVENT DMSO
NS 15000
DS 4
SWH 18115.941 Hz
FIDRES 0.552855 Hz
AQ 1.8087935 sec
RG 201.81
DW 27.600 usec
DE 6.50 usec
TE 294.6 K
CNST2 145.0000000
CNST11 1.0000000
D1 2.00000000 sec
D20 0.00689655 sec
TD0 1
SFO1 75.4752953 MHz
NUC1 13C
P1 10.00 usec
P2 20.00 usec
PLW1 41.00000000 W
SFO2 300.1312005 MHz
NUC2 1H
CPDPRG[2 waltz16
PCPD2 90.00 usec
PLW2 6.69999981 W
PLW12 0.16212000 W
F2 - Processing parameters
SI 32768
SF 75.4677885 MHz
WDW EM
SSB 0
LB 1.00 Hz
GB 0
PC 1.40

^{1}H NMR of 3-chloro-5-[(1-methyl-1*H*-tetrazol-5-yl)thio]-4*H*-1,2,6-thiadiazin-4-one (**11b**)

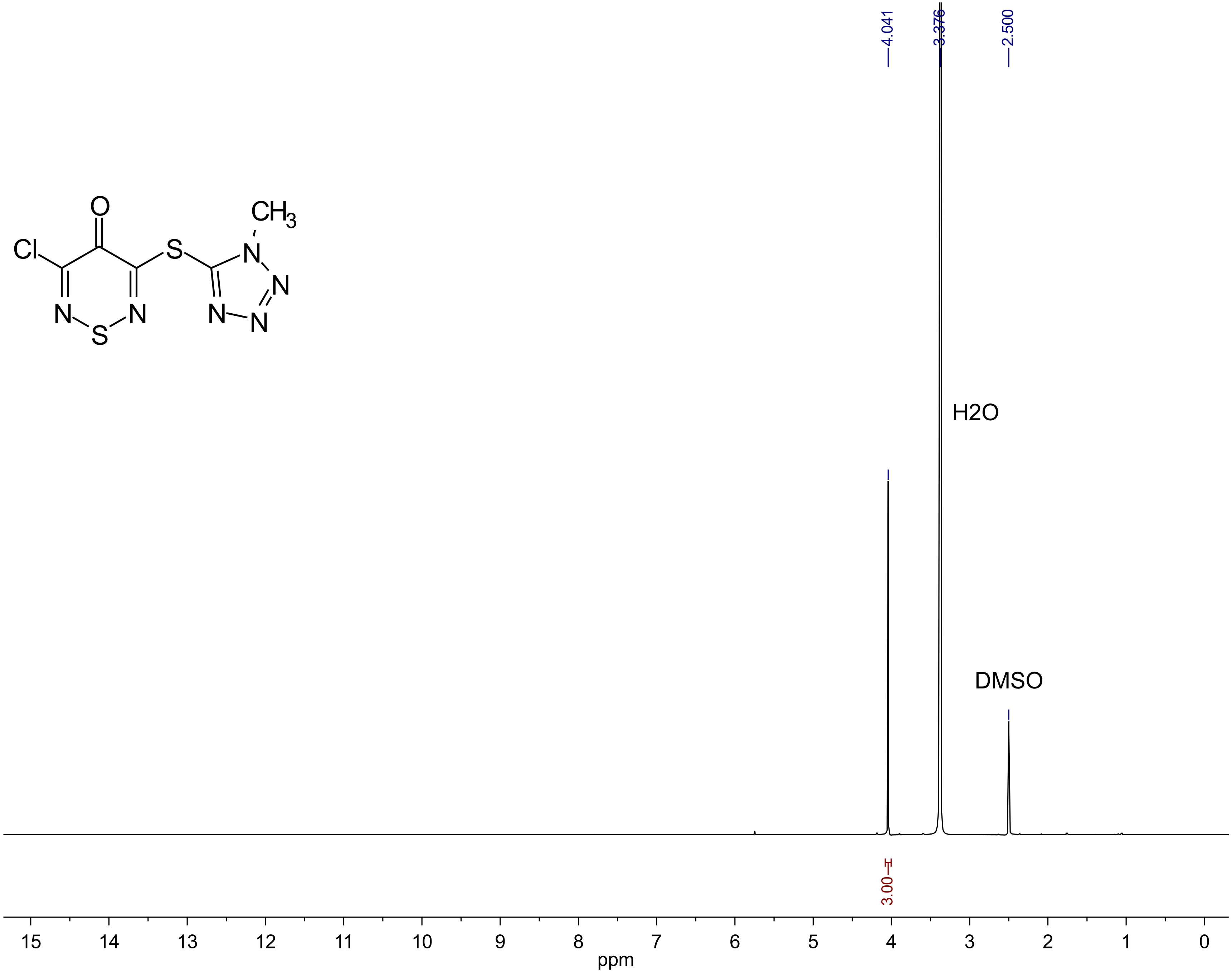

Current Data Parameters
NAME Kalogirou
EXPNO 717
PROCNO 1

F2 - Acquisition Parameters
Date_ 20191107
Time 10.13
INSTRUM spect
PROBHD 5 mm PABBO BB-
PULPROG zg30
TD 65536
SOLVENT DMSO
NS 16
DS 2
SWH 10000.000 Hz
FIDRES 0.152588 Hz
AQ 3.2767999 sec
RG 80.6
DW 50.000 usec
DE 6.50 usec
TE 297.4 K
D1 1.00000000 sec
TD0 1

======== CHANNEL f1 ========
SFO1 500.0361158 MHz
NUC1 1H
P1 12.00 usec
PLW1 14.50000000 W

F2 - Processing parameters
SI 65536
SF 500.0330326 MHz
WDW EM
SSB 0
LB 0.30 Hz
GB 0
PC 1.00

^{13}C NMR of 3-chloro-5-[(1-methyl-1*H*-tetrazol-5-yl)thio]-4*H*-1,2,6-thiadiazin-4-one (**11b**)

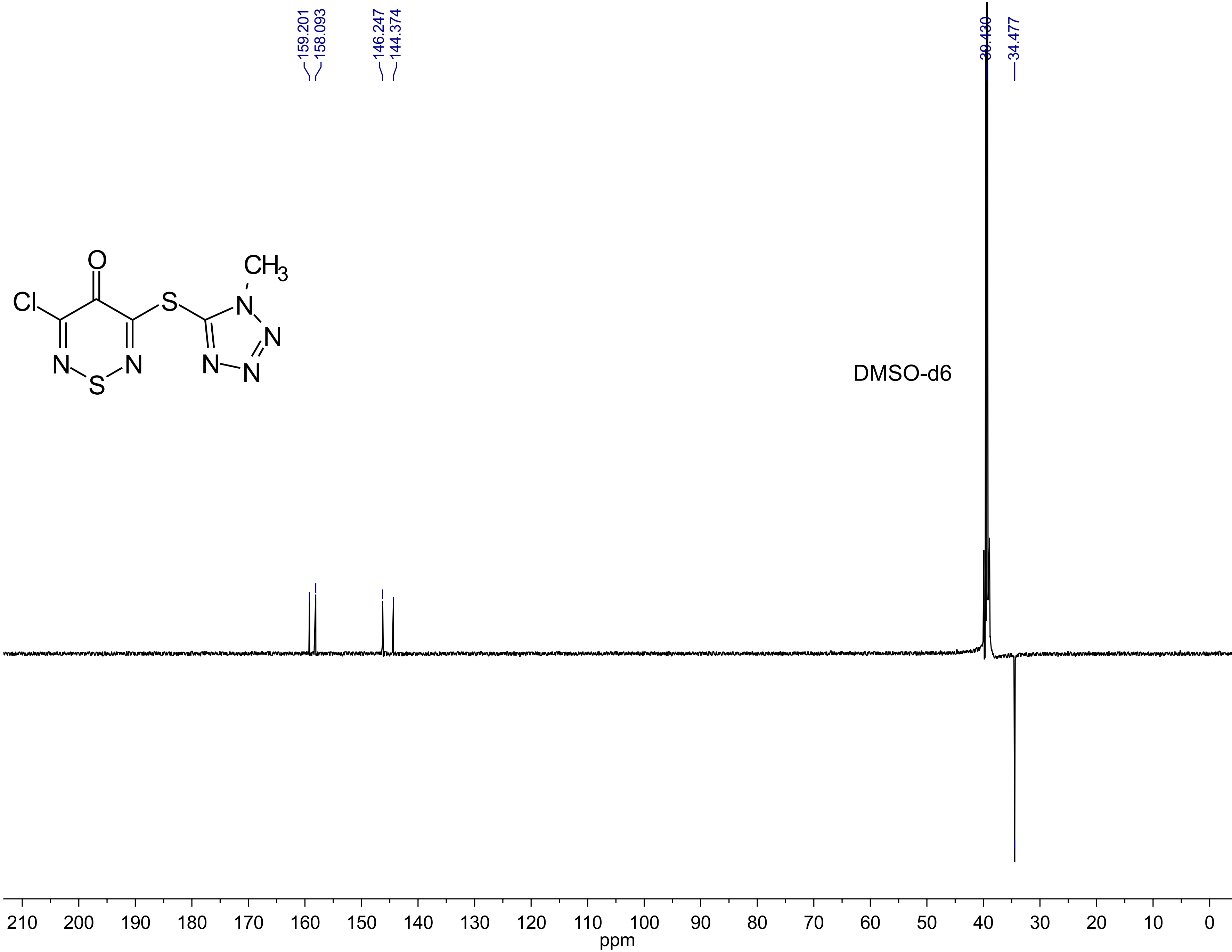

Current Data Parameters
NAME Kalogirou
EXPNO 715
PROCNO 1
F2 - Acquisition Parameters
Date_ 20191107
Time 9.20
INSTRUM spect
PROBHD 5 mm PABBO BB-
PULPROG jmod
TD 65536
SOLVENT DMSO
NS 15000
DS 4
SWH 29761.904 Hz
FIDRES 0.454131 Hz
AQ 1.1010048 sec
RG 2050
DW 16.800 usec
DE 6.50 usec
TE 299.0 K
CNST2 145.0000000
CNST11 1.0000000
D1 2.00000000 sec
D20 0.00689655 sec
TD0 1
======== CHANNEL f1 ========
SFO1 125.7459782 MHz
NUC1 13C
P1 9.20 usec
P2 18.40 usec
PLW1 140.00000000 W
======== CHANNEL f2 ========
SFO2 500.0350280 MHz
NUC2 1H
CPDPRG[2 waltz16
PCPD2 80.00 usec
PLW2 14.50000000 W
PLW12 0.32624999 W
F2 - Processing parameters
SI 32768
SF 125.7334711 MHz
WDW EM
SSB 0
LB 1.00 Hz
GB 0
PC 1.40

^{1}H NMR of 3-(benzo[*d*]oxazol-2-ylthio)-5-chloro-4*H*-1,2,6-thiadiazin-4-one (**11c**)

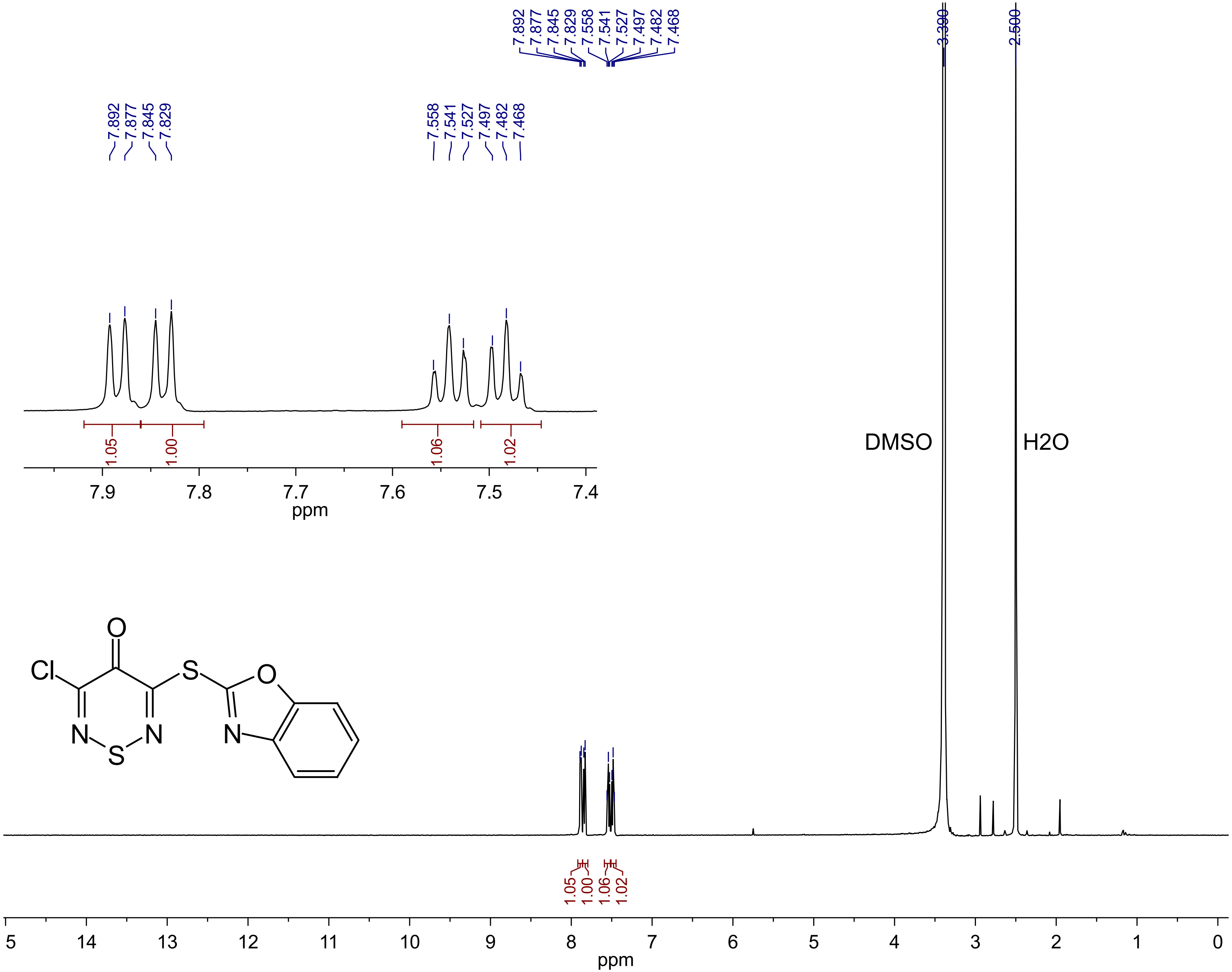

Current Data Parameters
NAME Kalogirou
EXPNO 736
PROCNO 1

F2 - Acquisition Parameters
Date_ 20191203
Time 14.12
INSTRUM spect
PROBHD 5 mm PABBO BB-
PULPROG zg30
TD 65536
SOLVENT DMSO
NS 16
DS 2
SWH 10000.000 Hz
FIDRES 0.152588 Hz
AQ 3.2767999 sec
RG 80.6
DW 50.000 usec
DE 6.50 usec
TE 294.8 K
D1 1.00000000 sec
TD0 1

======== CHANNEL f1 ========
SFO1 500.0361158 MHz
NUC1 1H
P1 12.00 usec
PLW1 14.50000000 W

F2 - Processing parameters
SI 65536
SF 500.0330321 MHz
WDW EM
SSB 0
LB 0.30 Hz
GB 0
PC 1.00

^{13}C NMR of 3-(benzo[*d*]oxazol-2-ylthio)-5-chloro-4*H*-1,2,6-thiadiazin-4-one (**11c**)

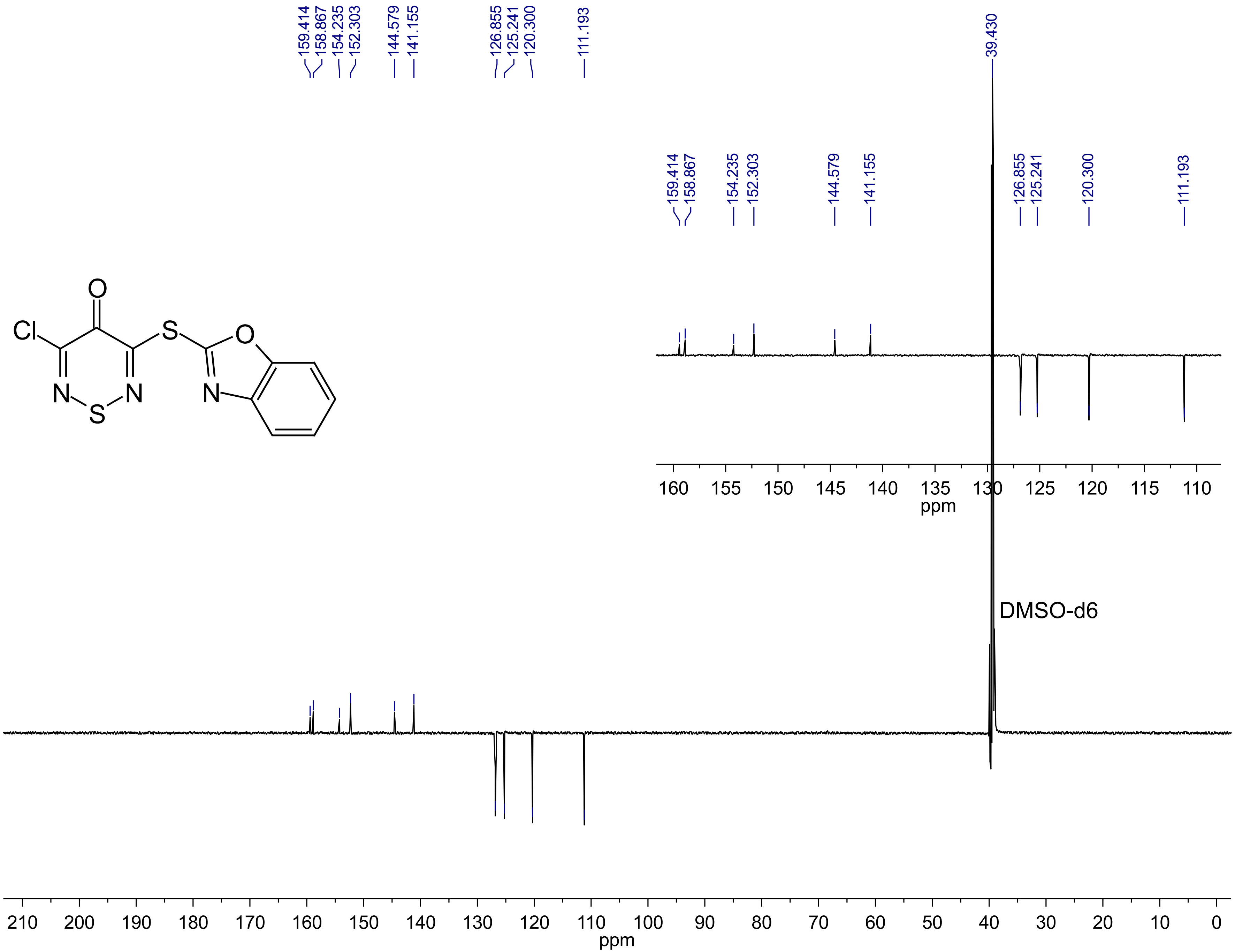

Current Data Parameters

NAME	Kalogirou
EXPNO	741
PROCNO	1

F2 - Acquisition Parameters

Date_	20191205
Time	9.06
INSTRUM	spect
PROBHD	5 mm PABBO BB-
PULPROG	jmod
TD	65536
SOLVENT	DMSO
NS	16000
DS	4
SWH	29761.904 Hz
FIDRES	0.454131 Hz
AQ	1.1010048 sec
RG	2050
DW	16.800 usec
DE	6.50 usec
TE	298.2 K
CNST2	145.0000000
CNST11	1.0000000
D1	2.00000000 sec
D20	0.00689655 sec
TD0	1

======== CHANNEL f1 ========

SFO1	125.7459782 MHz
NUC1	13C
P1	9.20 usec
P2	18.40 usec
PLW1	140.00000000 W

======== CHANNEL f2 ========

SFO2	500.0350280 MHz
NUC2	1H
CPDPRG[2	waltz16
PCPD2	80.00 usec
PLW2	14.50000000 W
PLW12	0.32624999 W

F2 - Processing parameters

SI	32768
SF	125.7334700 MHz
WDW	EM
SSB	0
LB	1.00 Hz
GB	0
PC	1.40

^{1}H NMR of *N*-{4-[(5-chloro-4-oxo-4*H*-1,2,6-thiadiazin-3-yl)thio]phenyl}acetamide (**11d**)

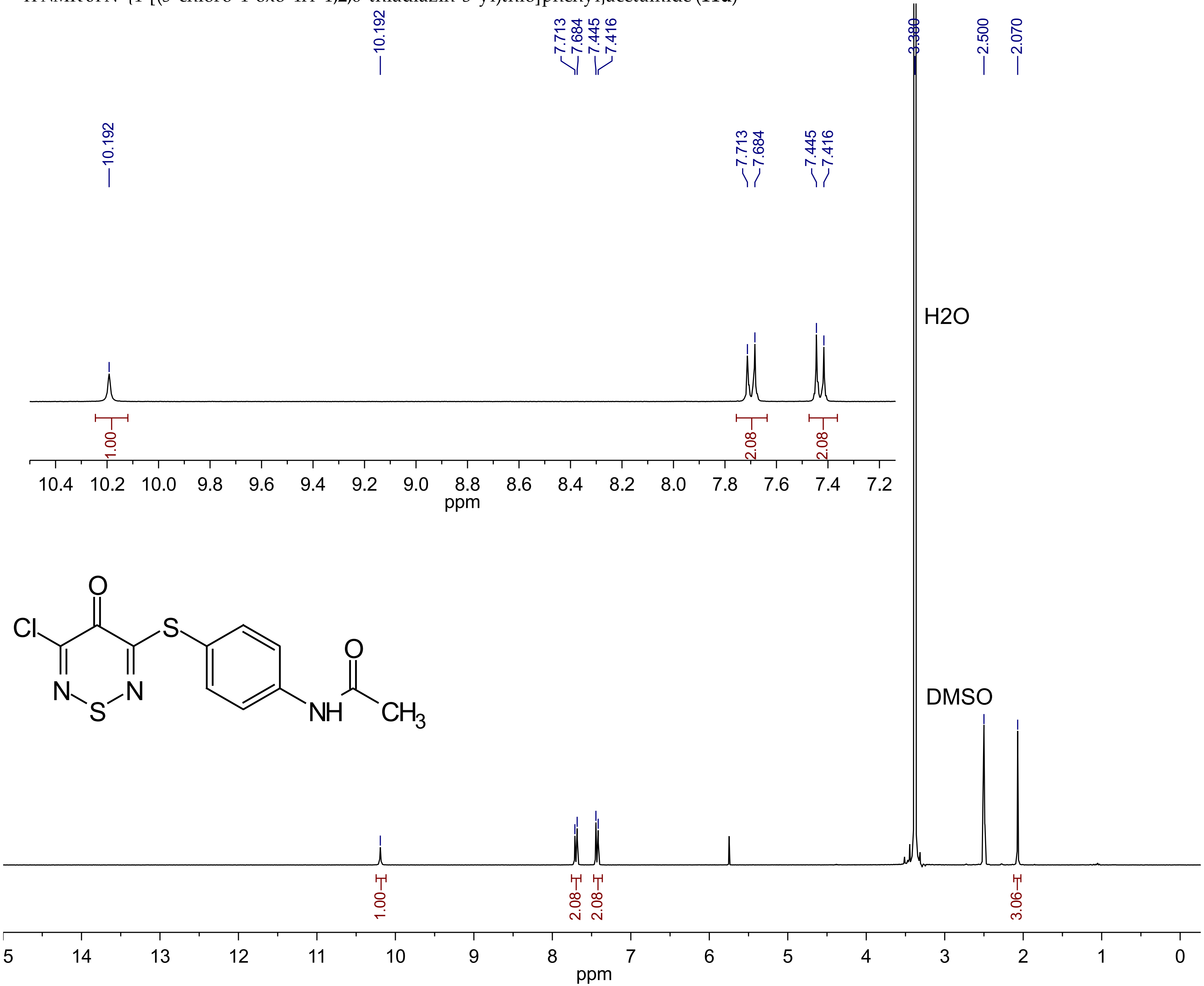

Current Data Parameters
NAME Andreas
EXPNO 201
PROCNO 1

F2 - Acquisition Parameters
Date_ 20191016
Time 18.20 h
INSTRUM spect
PROBHD Z104275_0375 (
PULPROG zg30
TD 65536
SOLVENT DMSO
NS 16
DS 2
SWH 6009.615 Hz
FIDRES 0.183399 Hz
AQ 5.4525952 sec
RG 201.81
DW 83.200 usec
DE 6.50 usec
TE 295.8 K
D1 1.00000000 sec
TD0 1
SFO1 300.1318533 MHz
NUC1 1H
P1 14.00 usec
PLW1 6.69999981 W

F2 - Processing parameters
SI 65536
SF 300.1300021 MHz
WDW EM
SSB 0
LB 0.30 Hz
GB 0
PC 1.00

^{13}C NMR of *N*-{4-[(5-chloro-4-oxo-4*H*-1,2,6-thiadiazin-3-yl)thio]phenyl}acetamide (**11d**)

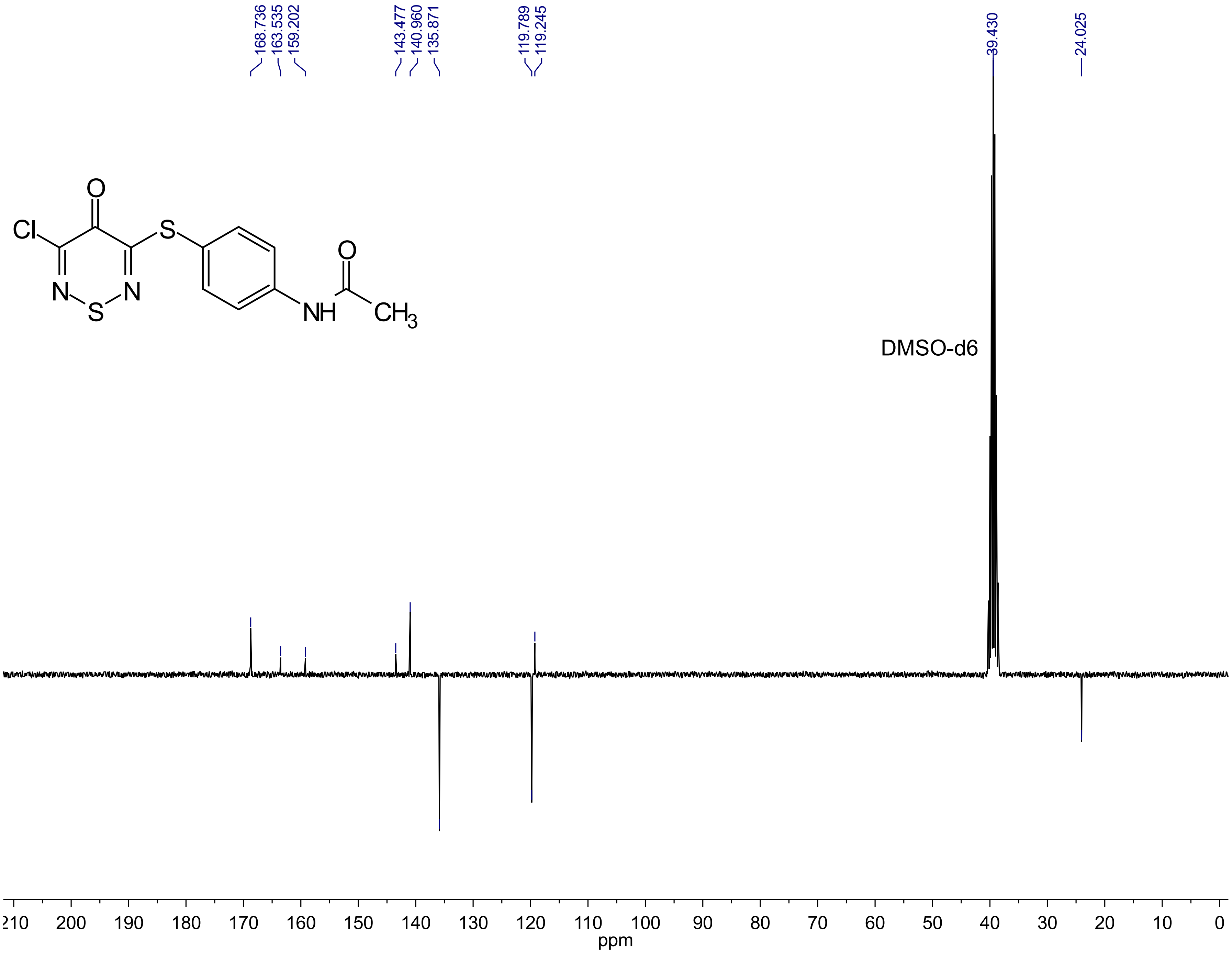

Current Data Parameters	
NAME	Andreas
EXPNO	202
PROCNO	1
F2 - Acquisition Parameters	
Date_	20191017
Time	9.26 h
INSTRUM	spect
PROBHD	Z104275_0375 (
PULPROG	jmod
TD	65536
SOLVENT	DMSO
NS	14000
DS	4
SWH	18115.941 Hz
FIDRES	0.552855 Hz
AQ	1.8087935 sec
RG	201.81
DW	27.600 usec
DE	6.50 usec
TE	296.0 K
CNST2	145.0000000
CNST11	1.0000000
D1	2.00000000 sec
D20	0.00689655 sec
TD0	1
SFO1	75.4752953 MHz
NUC1	13C
P1	10.00 usec
P2	20.00 usec
PLW1	41.00000000 W
SFO2	300.1312005 MHz
NUC2	1H
CPDPRG[2	waltz16
PCPD2	90.00 usec
PLW2	6.69999981 W
PLW12	0.16212000 W
F2 - Processing parameters	
SI	32768
SF	75.4677884 MHz
WDW	EM
SSB	0
LB	1.00 Hz
GB	0
PC	1.40

^{1}H NMR of 3-chloro-5-[(1-phenyl-1*H*-tetrazol-5-yl)thio]-4*H*-1,2,6-thiadiazin-4-one (**11e**)

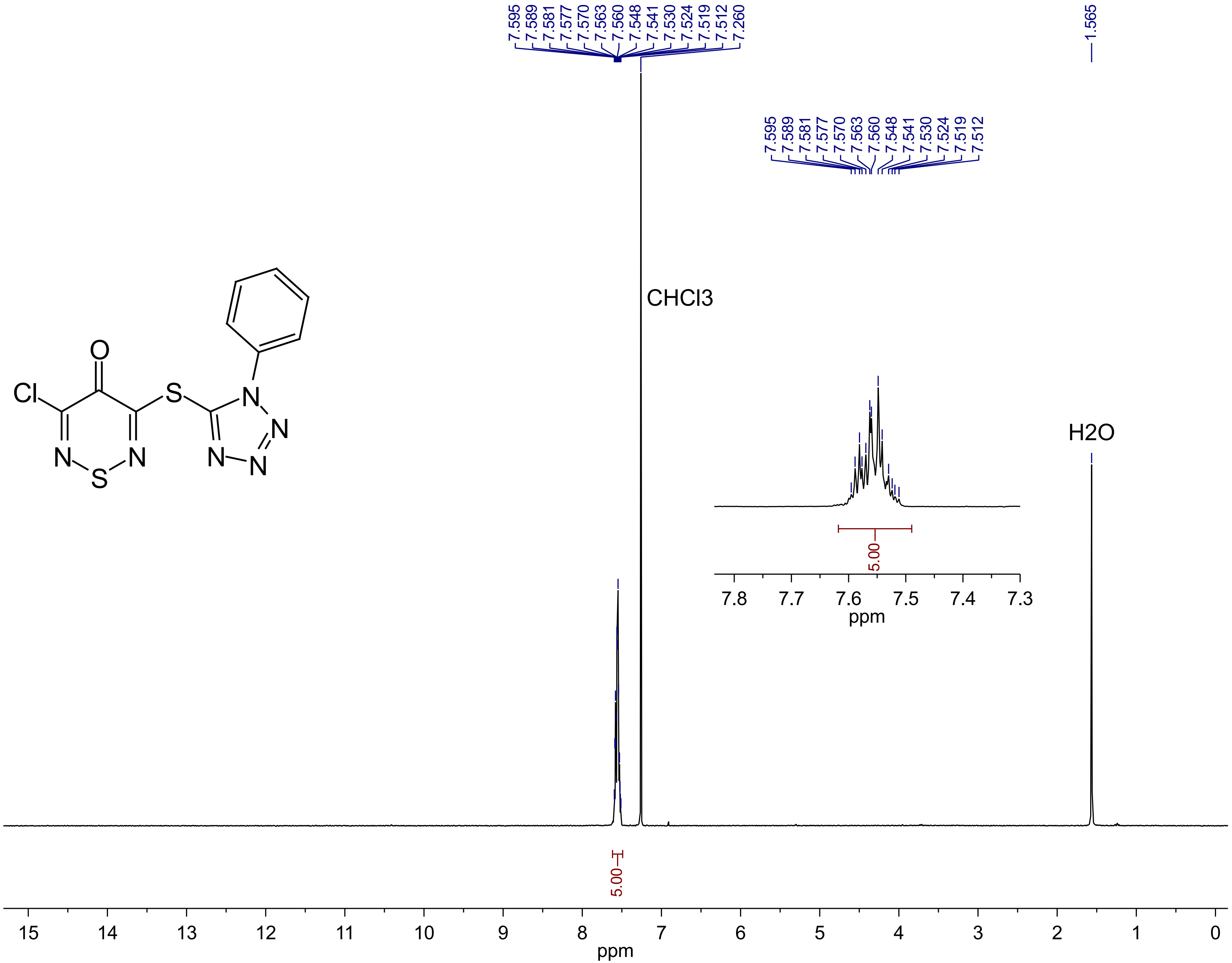

Current Data Parameters
NAME Andreas
EXPNO 215
PROCNO 1

F2 - Acquisition Parameters
Date_ 20200311
Time 19.08 h
INSTRUM spect
PROBHD Z104275_0375 (
PULPROG zg30
TD 65536
SOLVENT CDCl3
NS 16
DS 2
SWH 6009.615 Hz
FIDRES 0.183399 Hz
AQ 5.4525952 sec
RG 201.81
DW 83.200 usec
DE 6.50 usec
TE 294.1 K
D1 1.00000000 sec
TD0 1
SFO1 300.1318533 MHz
NUC1 1H
P1 14.00 usec
PLW1 8.19999981 W

F2 - Processing parameters
SI 65536
SF 300.1300074 MHz
WDW EM
SSB 0
LB 0.30 Hz
GB 0
PC 1.00

^{13}C NMR of 3-chloro-5-[(1-phenyl-1*H*-tetrazol-5-yl)thio]-4*H*-1,2,6-thiadiazin-4-one (**11e**)

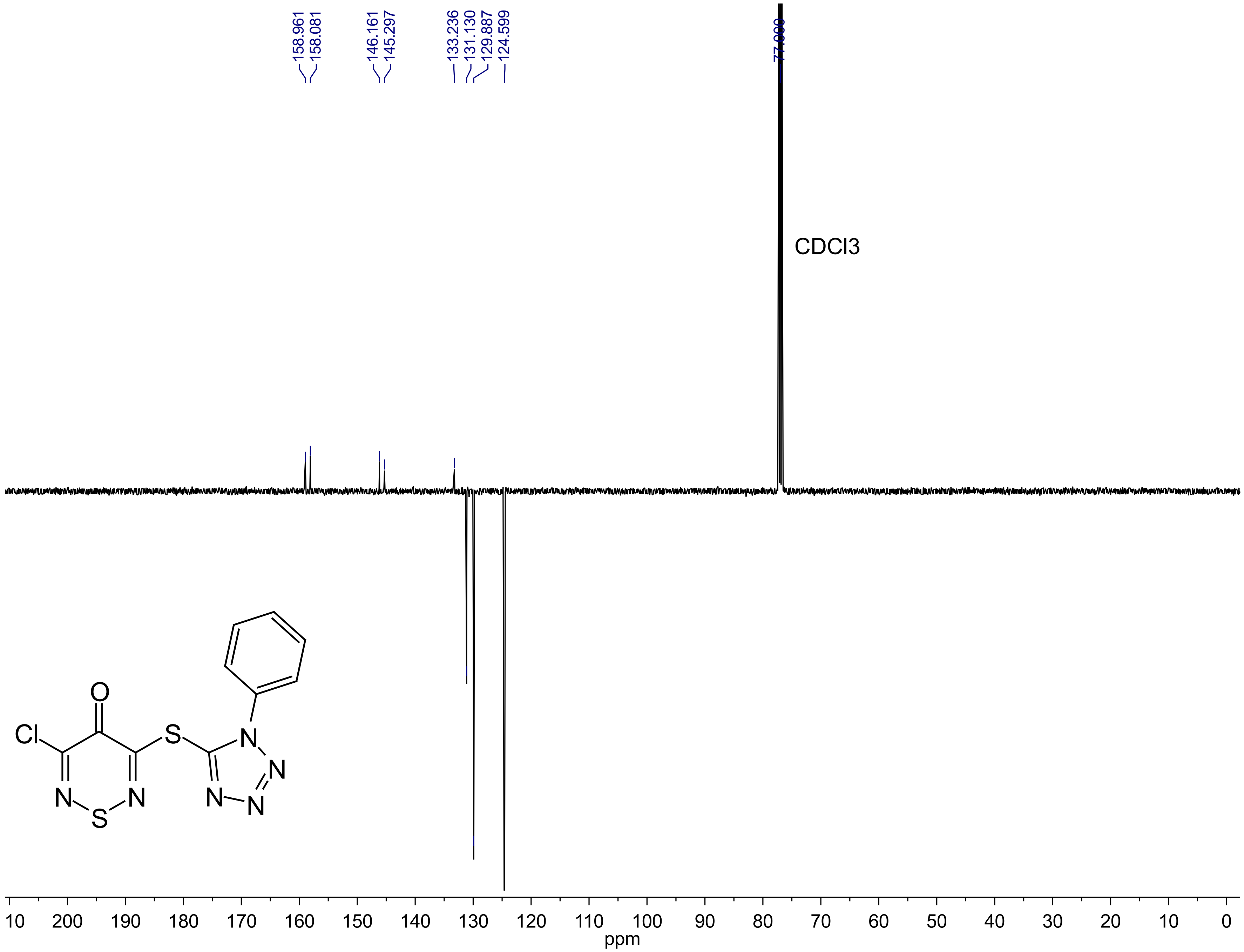

Current Data Parameters
NAME Kalogirou
EXPNO 788
PROCNO 1
F2 - Acquisition Parameters
Date_ 20200312
Time 9.03
INSTRUM spect
PROBHD 5 mm PABBO BB-
PULPROG jmod
TD 65536
SOLVENT CDCl3
NS 15000
DS 4
SWH 29761.904 Hz
FIDRES 0.454131 Hz
AQ 1.1010048 sec
RG 2050
DW 16.800 usec
DE 6.50 usec
TE 298.0 K
CNST2 145.0000000
CNST11 1.0000000
D1 2.00000000 sec
D20 0.00689655 sec
TD0 1
======== CHANNEL f1 ========
SFO1 125.7459782 MHz
NUC1 13C
P1 9.20 usec
P2 18.40 usec
PLW1 140.00000000 W
======== CHANNEL f2 ========
SFO2 500.0350280 MHz
NUC2 1H
CPDPRG[2 waltz16
PCPD2 80.00 usec
PLW2 14.50000000 W
PLW12 0.32624999 W
F2 - Processing parameters
SI 32768
SF 125.7334075 MHz
WDW EM
SSB 0
LB 1.00 Hz
GB 0
PC 1.40

^{1}H NMR of 3-chloro-5-[(4-methyl-4*H*-1,2,4-triazol-3-yl)thio]-4*H*-1,2,6-thiadiazin-4-one (**11f**)

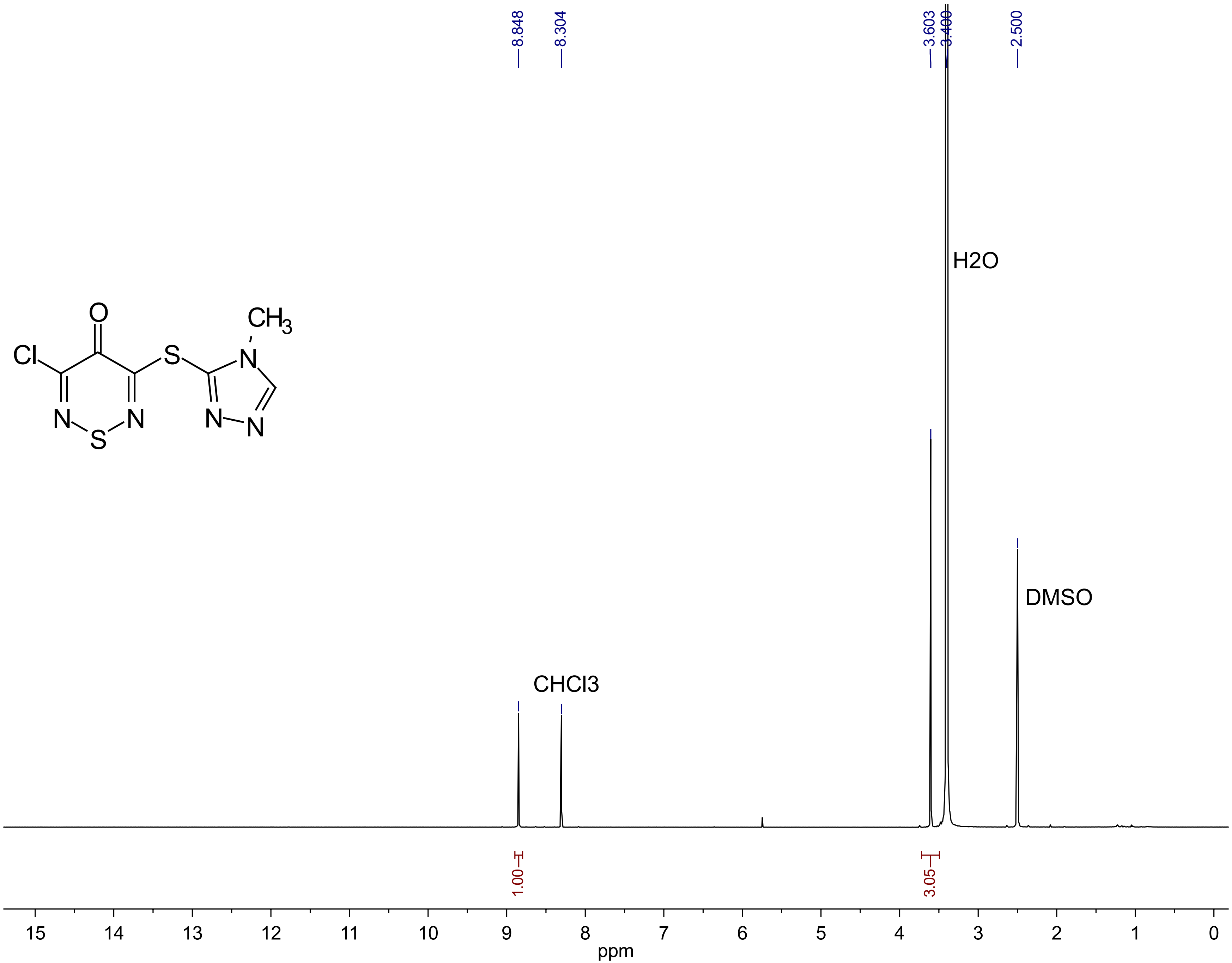

Current Data Parameters
NAME Kalogirou
EXPNO 792
PROCNO 1

F2 - Acquisition Parameters
Date_ 20200321
Time 20.04
INSTRUM spect
PROBHD 5 mm PABBO BB-
PULPROG zg30
TD 65536
SOLVENT DMSO
NS 16
DS 2
SWH 10000.000 Hz
FIDRES 0.152588 Hz
AQ 3.2767999 sec
RG 71.8
DW 50.000 usec
DE 6.50 usec
TE 294.6 K
D1 1.00000000 sec
TD0 1

======== CHANNEL f1 ========
SFO1 500.0361158 MHz
NUC1 1H
P1 12.00 usec
PLW1 14.50000000 W

F2 - Processing parameters
SI 65536
SF 500.0330320 MHz
WDW EM
SSB 0
LB 0.30 Hz
GB 0
PC 1.00

^{13}C NMR of 3-chloro-5-[(4-methyl-4*H*-1,2,4-triazol-3-yl)thio]-4*H*-1,2,6-thiadiazin-4-one (**11f**)

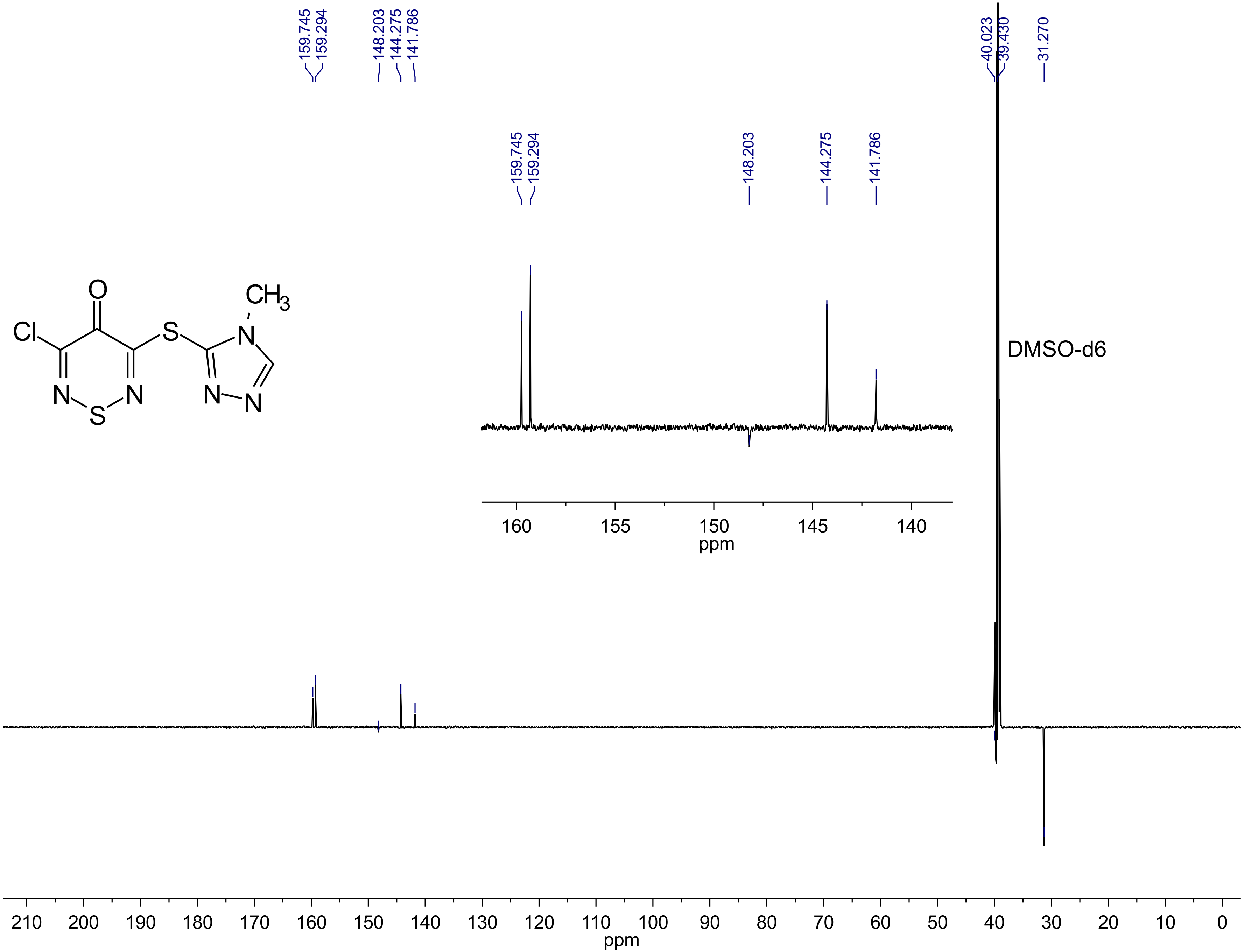

Current Data Parameters
NAME Kalogirou
EXPNO 793
PROCNO 1
F2 - Acquisition Parameters
Date_ 20200322
Time 15.26
INSTRUM spect
PROBHD 5 mm PABBO BB-
PULPROG jmod
TD 65536
SOLVENT DMSO
NS 22000
DS 4
SWH 29761.904 Hz
FIDRES 0.454131 Hz
AQ 1.1010048 sec
RG 2050
DW 16.800 usec
DE 6.50 usec
TE 296.1 K
CNST2 145.0000000
CNST11 1.0000000
D1 2.00000000 sec
D20 0.00689655 sec
TD0 1
======== CHANNEL f1 ========
SFO1 125.7459782 MHz
NUC1 13C
P1 9.20 usec
P2 18.40 usec
PLW1 140.00000000 W
======== CHANNEL f2 ========
SFO2 500.0350280 MHz
NUC2 1H
CPDPRG[2 waltz16
PCPD2 80.00 usec
PLW2 14.50000000 W
PLW12 0.32624999 W
F2 - Processing parameters
SI 32768
SF 125.7334682 MHz
WDW EM
SSB 0
LB 1.00 Hz
GB 0
PC 1.40

^{1}H NMR of 3-chloro-5-(thiazol-2-ylthio)-4*H*-1,2,6-thiadiazin-4-one (**11g**)

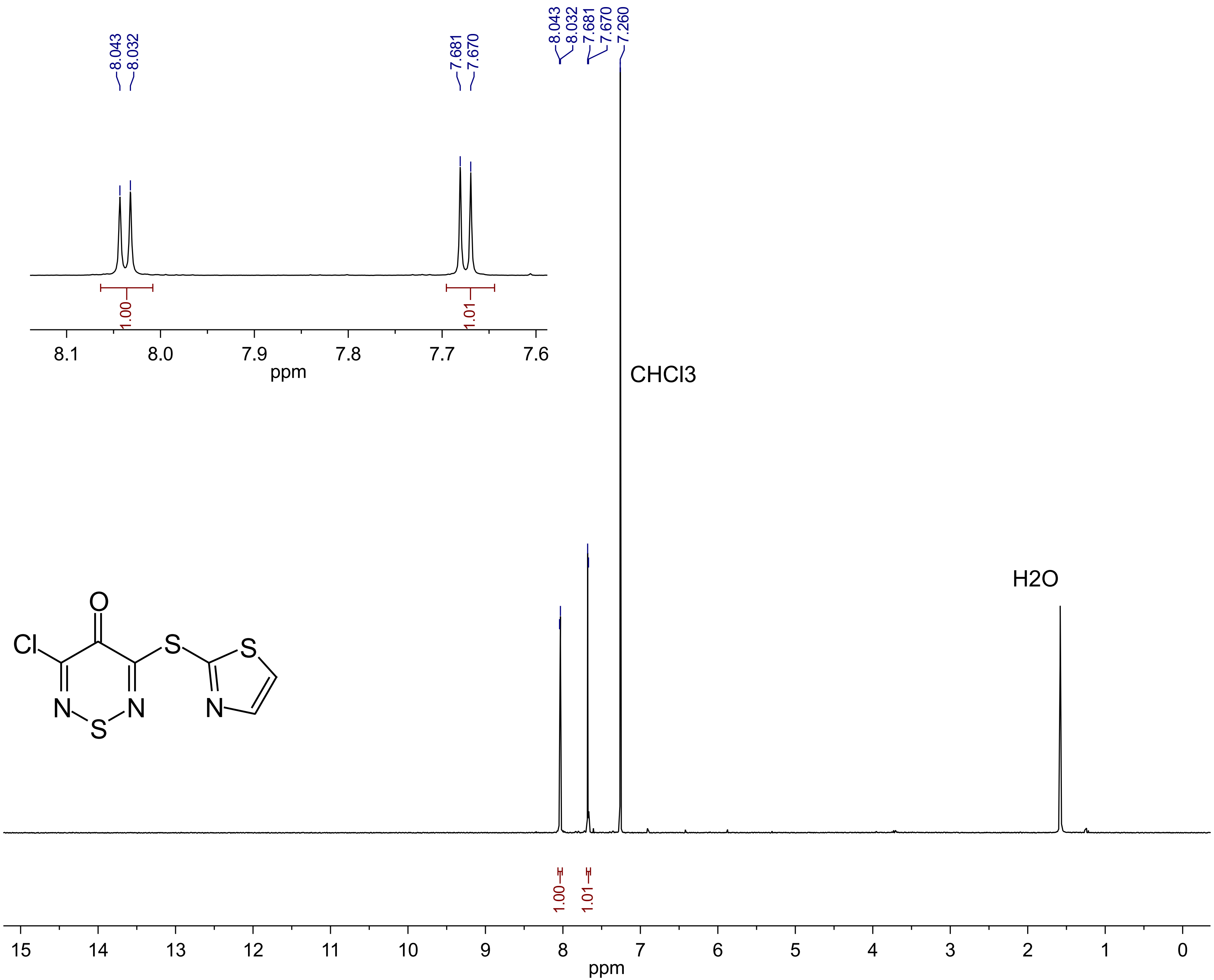

Current Data Parameters
NAME Andreas
EXPNO 216
PROCNO 1

F2 - Acquisition Parameters
Date_ 20200312
Time 18.46 h
INSTRUM spect
PROBHD Z104275_0375 (
PULPROG zg30
TD 65536
SOLVENT CDCl3
NS 16
DS 2
SWH 6009.615 Hz
FIDRES 0.183399 Hz
AQ 5.4525952 sec
RG 201.81
DW 83.200 usec
DE 6.50 usec
TE 294.2 K
D1 1.00000000 sec
TD0 1
SFO1 300.1318533 MHz
NUC1 1H
P1 14.00 usec
PLW1 8.19999981 W

F2 - Processing parameters
SI 65536
SF 300.1300074 MHz
WDW EM
SSB 0
LB 0.30 Hz
GB 0
PC 1.00

^{13}C NMR of 3-chloro-5-(thiazol-2-ylthio)-4*H*-1,2,6-thiadiazin-4-one (**11g**)

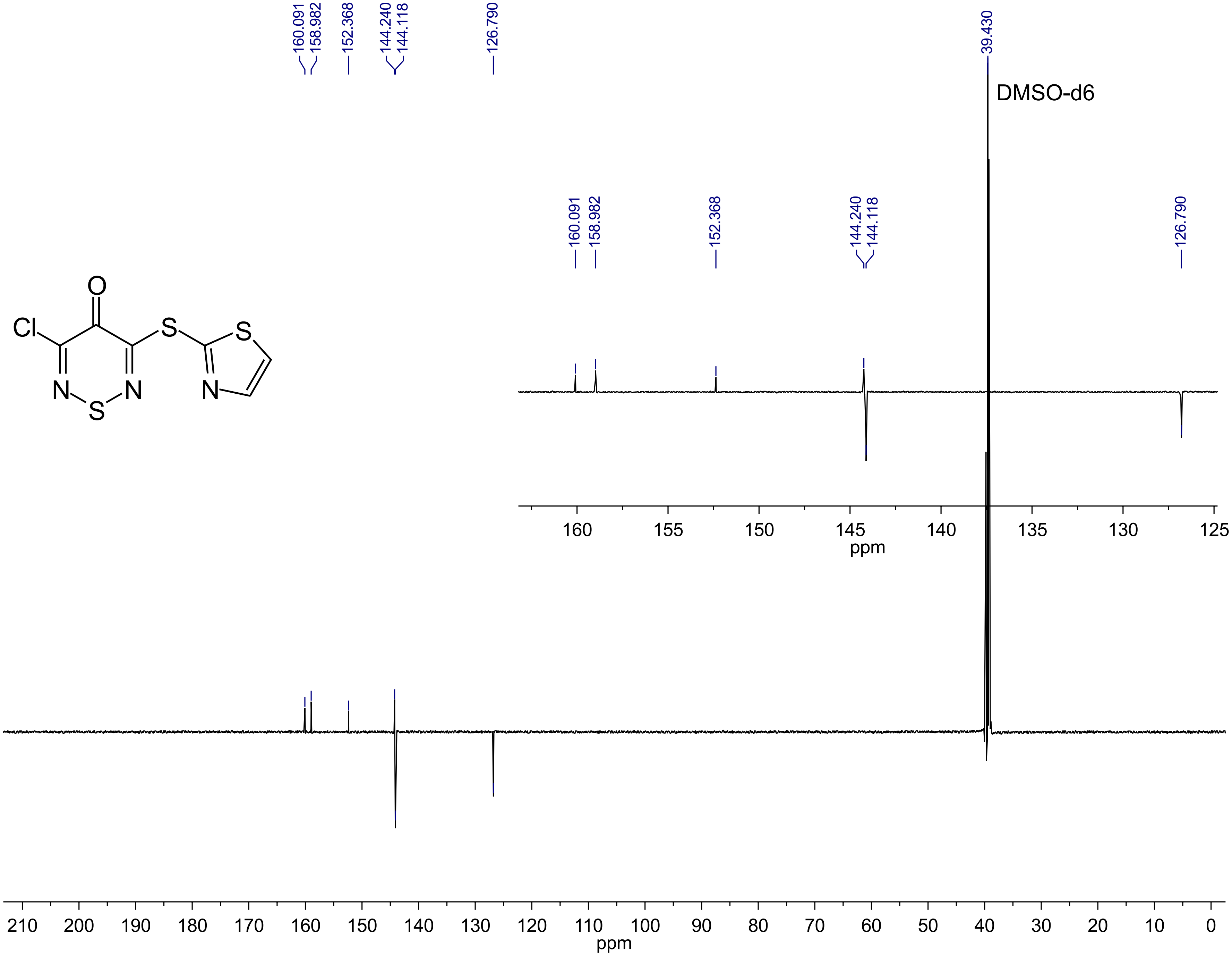

Current Data Parameters
NAME Kalogirou
EXPNO 789
PROCNO 1
F2 - Acquisition Parameters
Date_ 20200315
Time 9.10
INSTRUM spect
PROBHD 5 mm PABBO BB-
PULPROG jmod
TD 65536
SOLVENT DMSO
NS 15000
DS 4
SWH 29761.904 Hz
FIDRES 0.454131 Hz
AQ 1.1010048 sec
RG 2050
DW 16.800 usec
DE 6.50 usec
TE 298.0 K
CNST2 145.0000000
CNST11 1.0000000
D1 2.00000000 sec
D20 0.00689655 sec
TD0 1
======== CHANNEL f1 ========
SFO1 125.7459782 MHz
NUC1 13C
P1 9.20 usec
P2 18.40 usec
PLW1 140.00000000 W
======== CHANNEL f2 ========
SFO2 500.0350280 MHz
NUC2 1H
CPDPRG[2 waltz16
PCPD2 80.00 usec
PLW2 14.50000000 W
PLW12 0.32624999 W
F2 - Processing parameters
SI 32768
SF 125.7334692 MHz
WDW EM
SSB 0
LB 1.00 Hz
GB 0
PC 1.40

^{1}H NMR of 3-chloro-5-[(6-ethoxybenzo[*d*]thiazol-2-yl)thio]-4*H*-1,2,6-thiadiazin-4-one (**11i**)

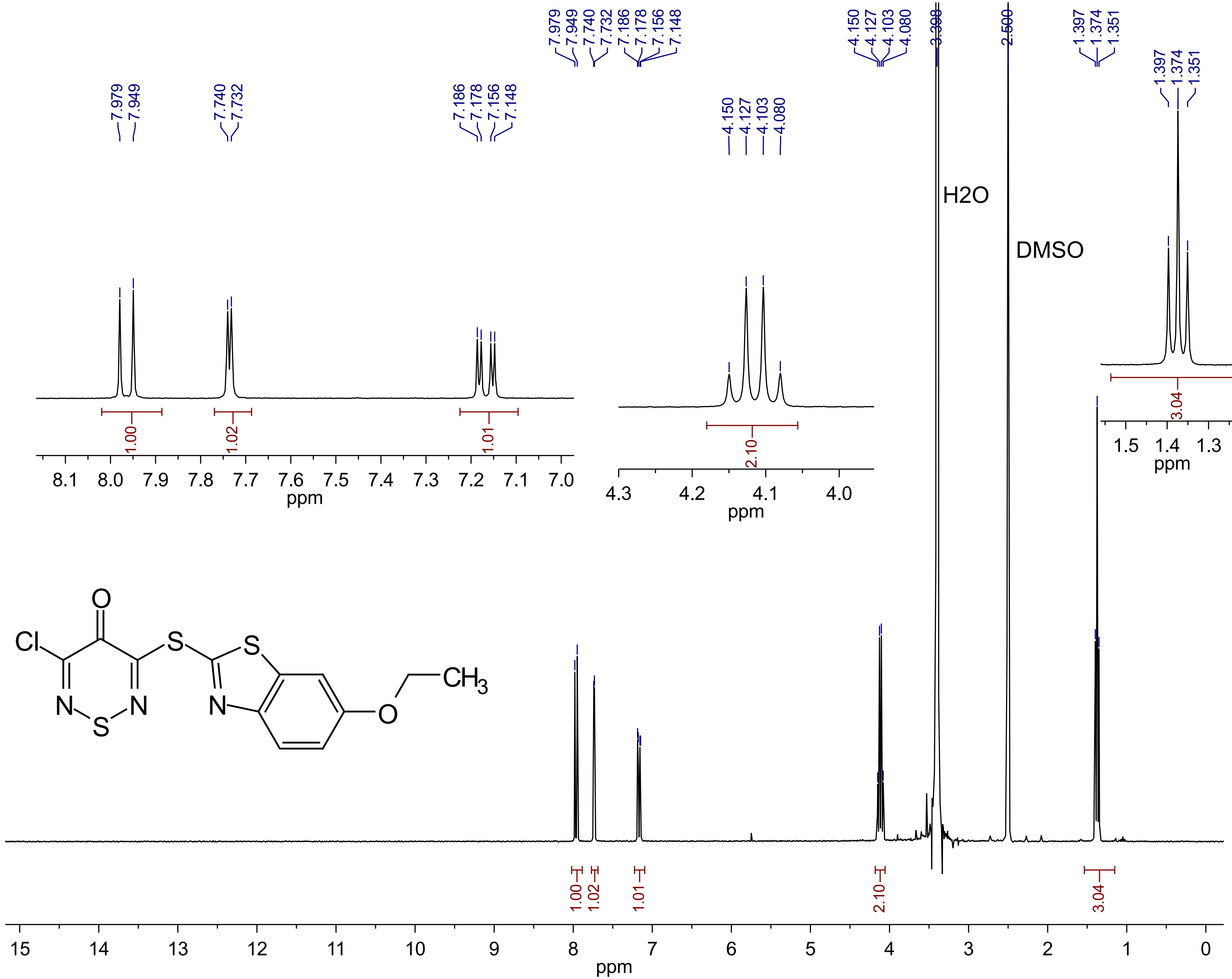

Current Data Parameters
NAME Andreas
EXPNO 218
PROCNO 1

F2 - Acquisition Parameters
Date_ 20200316
Time 19.13 h
INSTRUM spect
PROBHD Z104275_0375 (
PULPROG zg30
TD 65536
SOLVENT DMSO
NS 16
DS 2
SWH 6009.615 Hz
FIDRES 0.183399 Hz
AQ 5.4525952 sec
RG 201.81
DW 83.200 usec
DE 6.50 usec
TE 294.3 K
D1 1.00000000 sec
TD0 1
SFO1 300.1318533 MHz
NUC1 1H
P1 14.00 usec
PLW1 6.69999981 W

F2 - Processing parameters
SI 65536
SF 300.1300025 MHz
WDW EM
SSB 0
LB 0.30 Hz
GB 0
PC 1.00

^{13}C NMR of 3-chloro-5-[(6-ethoxybenzo[*d*]thiazol-2-yl)thio]-4*H*-1,2,6-thiadiazin-4-one (**11i**)

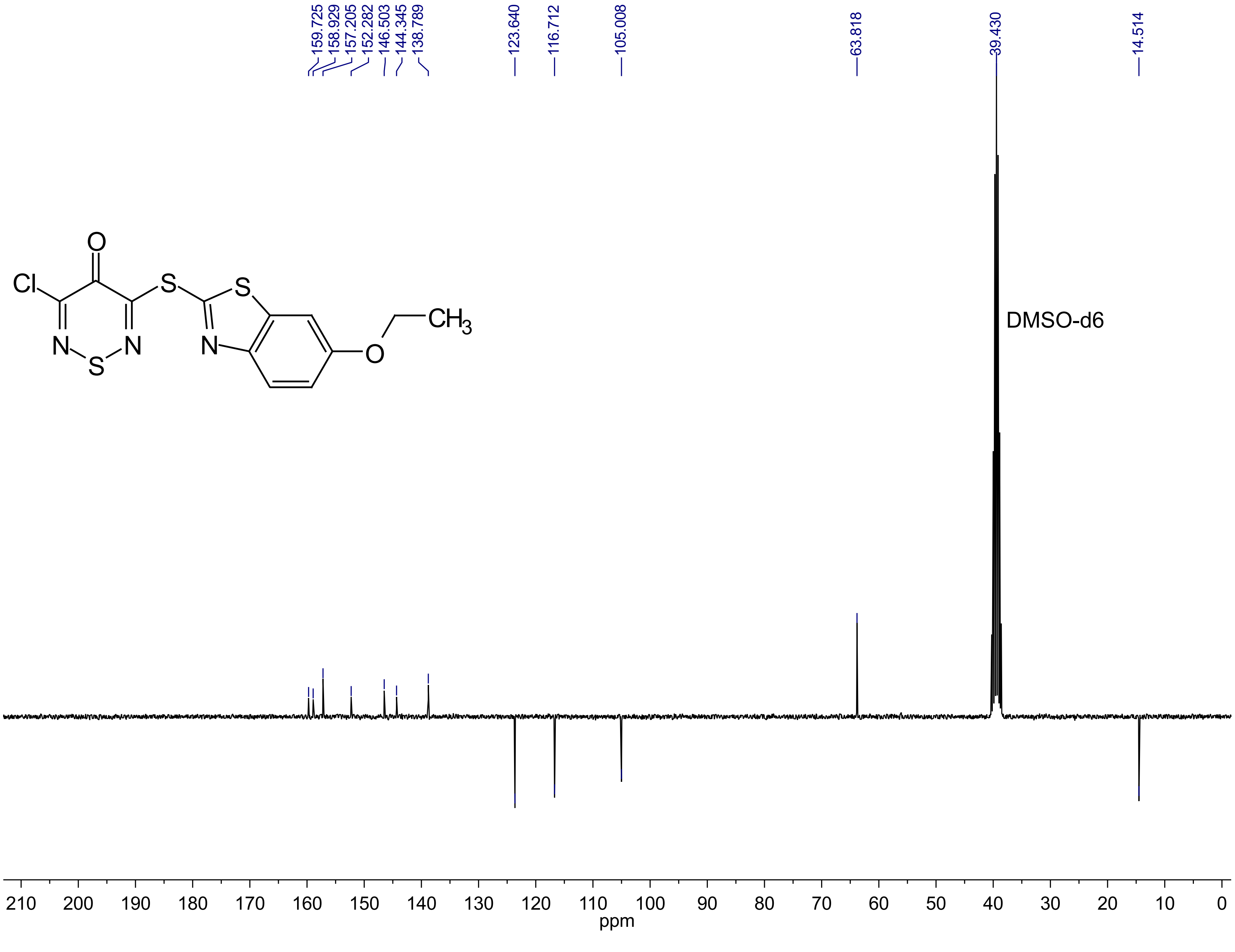

Current Data Parameters
NAME Andreas
EXPNO 219
PROCNO 1
F2 - Acquisition Parameters
Date_ 20200317
Time 9.19 h
INSTRUM spect
PROBHD Z104275_0375 (
PULPROG jmod
TD 65536
SOLVENT DMSO
NS 13000
DS 4
SWH 18115.941 Hz
FIDRES 0.552855 Hz
AQ 1.8087935 sec
RG 201.81
DW 27.600 usec
DE 6.50 usec
TE 294.8 K
CNST2 145.0000000
CNST11 1.0000000
D1 2.00000000 sec
D20 0.00689655 sec
TD0 1
SFO1 75.4752953 MHz
NUC1 13C
P1 10.00 usec
P2 20.00 usec
PLW1 41.00000000 W
SFO2 300.1312005 MHz
NUC2 1H
CPDPRG[2 waltz16
PCPD2 80.00 usec
PLW2 8.19999981 W
PLW12 0.25112000 W
F2 - Processing parameters
SI 32768
SF 75.4677873 MHz
WDW EM
SSB 0
LB 1.00 Hz
GB 0
PC 1.40

^{1}H NMR of 2-{3-chloro-5-[(4-methylpyrimidin-2-yl)thio]-4*H*-1,2,6-thiadiazin-4-ylidene}malononitrile (**12a**)

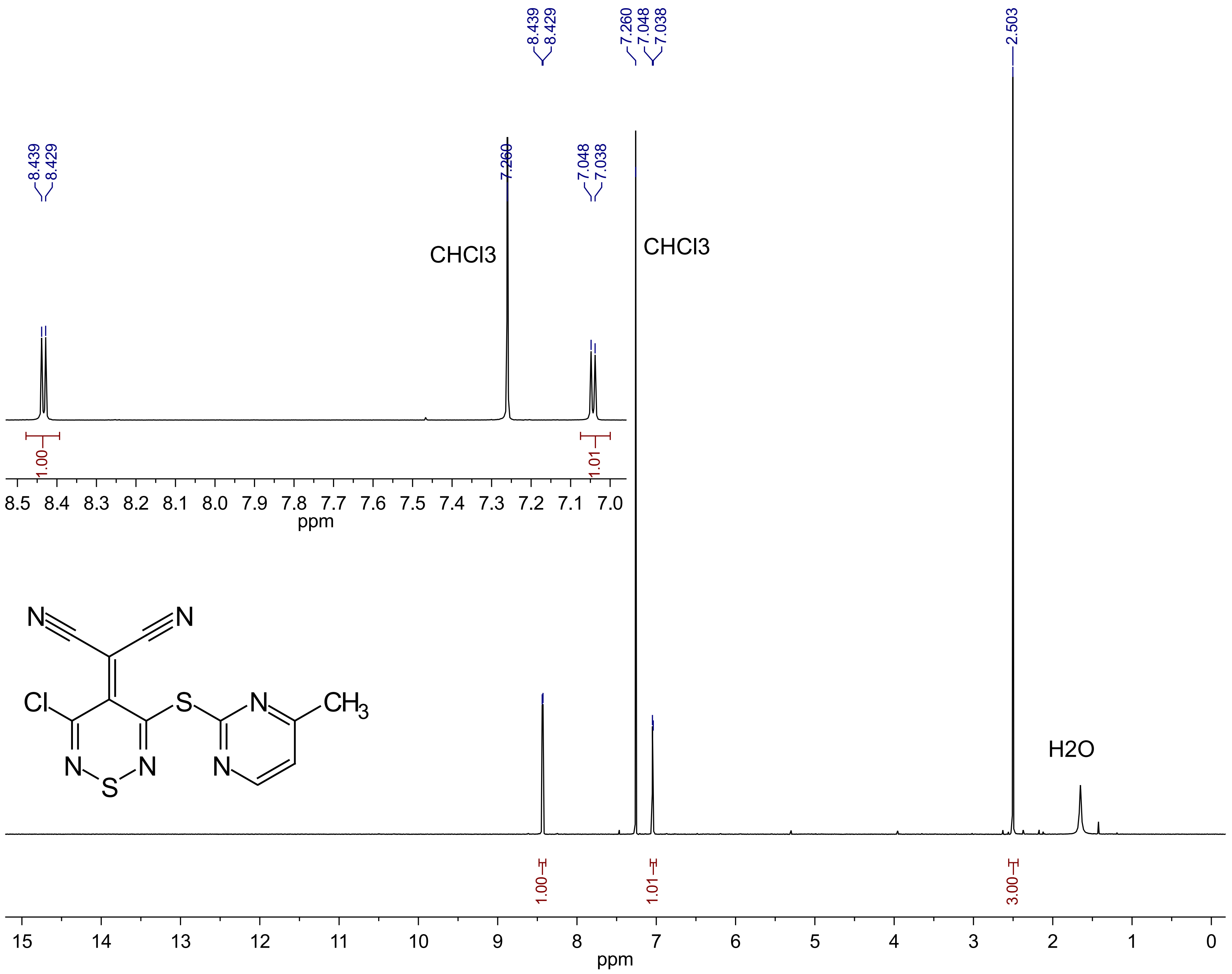

Current Data Parameters	
NAME	Kalogirou
EXPNO	723
PROCNO	1
F2 - Acquisition Parameters	
Date_	20191114
Time	13.42
INSTRUM	spect
PROBHD	5 mm PABBO BB-
PULPROG	zg30
TD	65536
SOLVENT	CDCl3
NS	16
DS	2
SWH	10000.000 Hz
FIDRES	0.152588 Hz
AQ	3.2767999 sec
RG	203
DW	50.000 usec
DE	6.50 usec
TE	296.8 K
D1	1.00000000 sec
TD0	1
======== CHANNEL f1 ========	
SFO1	500.0361158 MHz
NUC1	1H
P1	12.00 usec
PLW1	14.50000000 W
F2 - Processing parameters	
SI	65536
SF	500.0330406 MHz
WDW	EM
SSB	0
LB	0.30 Hz
GB	0
PC	1.00

^{13}C NMR of 2-{3-chloro-5-[(4-methylpyrimidin-2-yl)thio]-4*H*-1,2,6-thiadiazin-4-ylidene}malononitrile (**12a**)

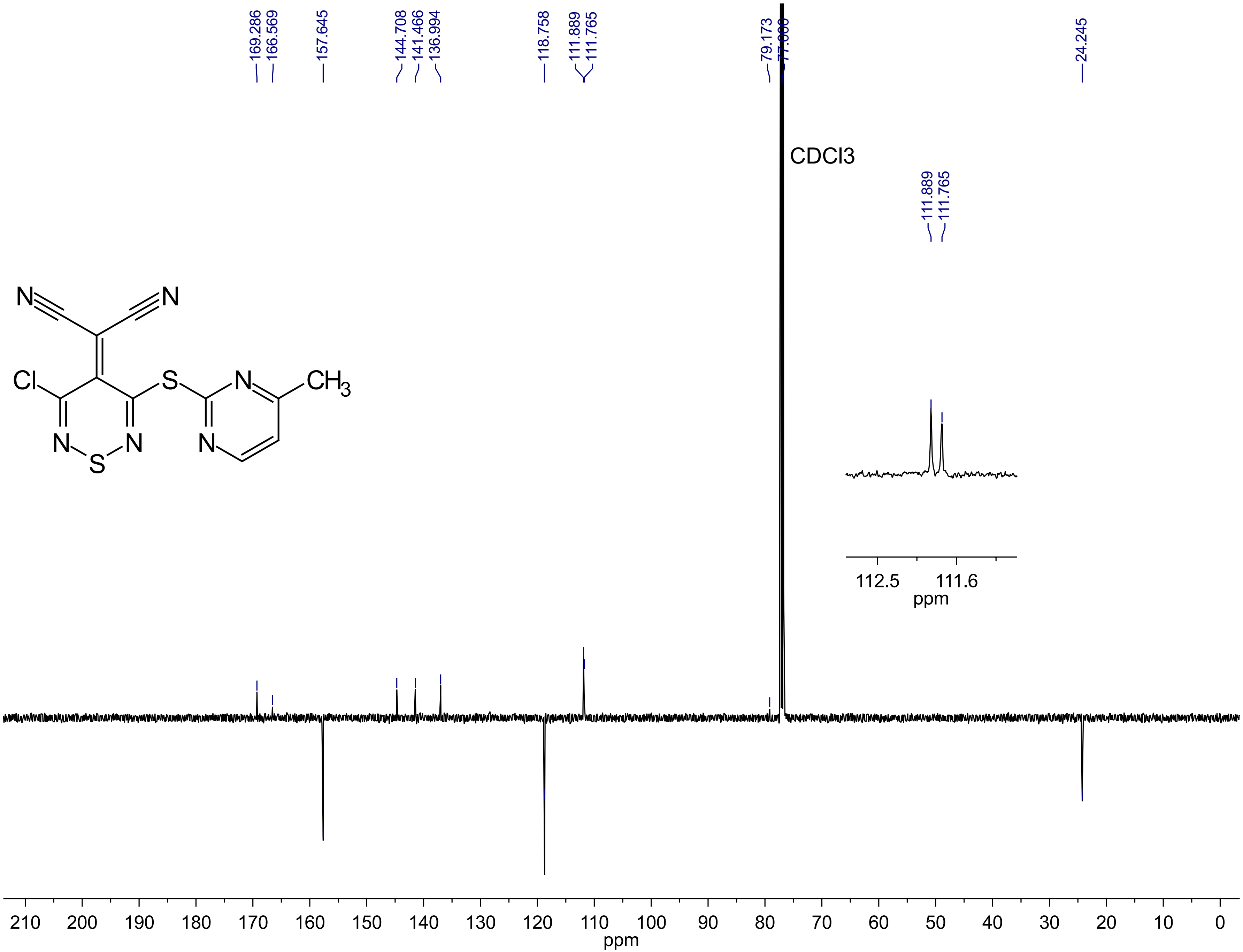

Current Data Parameters
NAME Kalogirou
EXPNO 722
PROCNO 1
F2 - Acquisition Parameters
Date_ 20191114
Time 9.18
INSTRUM spect
PROBHD 5 mm PABBO BB-
PULPROG jmod
TD 65536
SOLVENT CDCl3
NS 14500
DS 4
SWH 29761.904 Hz
FIDRES 0.454131 Hz
AQ 1.1010048 sec
RG 2050
DW 16.800 usec
DE 6.50 usec
TE 298.9 K
CNST2 145.0000000
CNST11 1.0000000
D1 2.00000000 sec
D20 0.00689655 sec
TD0 1
======== CHANNEL f1 ========
SFO1 125.7459782 MHz
NUC1 13C
P1 9.20 usec
P2 18.40 usec
PLW1 140.00000000 W
======== CHANNEL f2 ========
SFO2 500.0350280 MHz
NUC2 1H
CPDPRG[2 waltz16
PCPD2 80.00 usec
PLW2 14.50000000 W
PLW12 0.32624999 W
F2 - Processing parameters
SI 32768
SF 125.7334076 MHz
WDW EM
SSB 0
LB 1.00 Hz
GB 0
PC 1.40

^{1}H NMR of 2-{3-chloro-5-[(1-methyl-1*H*-tetrazol-5-yl)thio]-4*H*-1,2,6-thiadiazin-4-ylidene}malononitrile (**12b**)

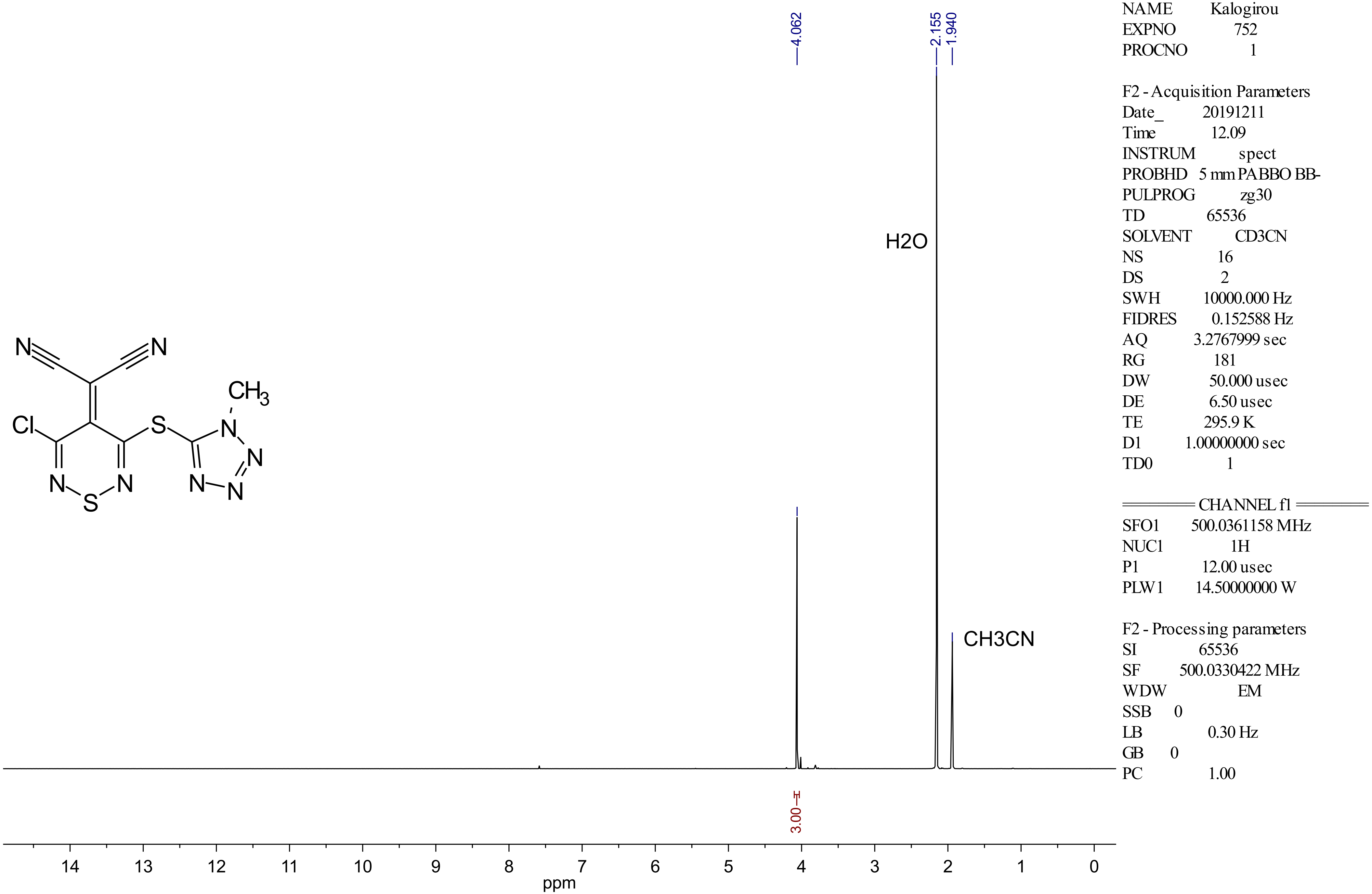

^{13}C NMR of 2-{3-chloro-5-[(1-methyl-1*H*-tetrazol-5-yl)thio]-4*H*-1,2,6-thiadiazin-4-ylidene}malononitrile (**12b**)

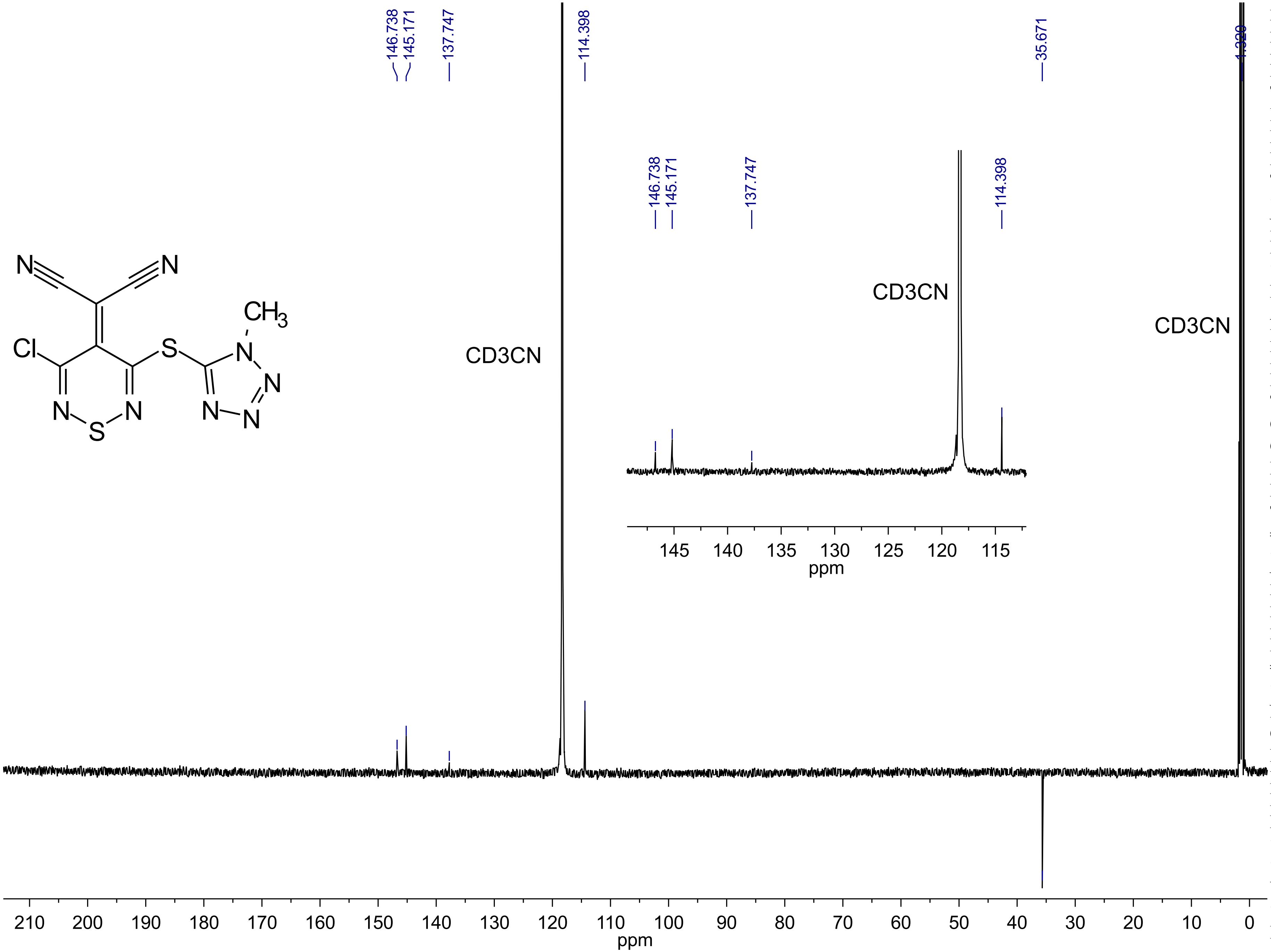

Current Data Parameters
NAME Kalogirou
EXPNO 751
PROCNO 1
F2 - Acquisition Parameters
Date_ 20191211
Time 9.10
INSTRUM spect
PROBHD 5 mm PABBO BB-
PULPROG jmod
TD 65536
SOLVENT CD3CN
NS 15000
DS 4
SWH 29761.904 Hz
FIDRES 0.454131 Hz
AQ 1.1010048 sec
RG 2050
DW 16.800 usec
DE 6.50 usec
TE 298.0 K
CNST2 145.0000000
CNST11 1.0000000
D1 2.00000000 sec
D20 0.00689655 sec
TD0 1
======== CHANNEL f1 ========
SFO1 125.7459782 MHz
NUC1 13C
P1 9.20 usec
P2 18.40 usec
PLW1 140.00000000 W
======== CHANNEL f2 ========
SFO2 500.0350280 MHz
NUC2 1H
CPDPRG[2 waltz16
PCPD2 80.00 usec
PLW2 14.50000000 W
PLW12 0.32624999 W
F2 - Processing parameters
SI 32768
SF 125.7332892 MHz
WDW EM
SSB 0
LB 1.00 Hz
GB 0
PC 1.40

^{1}H NMR of 2-[3-(benzo[*d*]oxazol-2-ylthio)-5-chloro-4*H*-1,2,6-thiadiazin-4-ylidene]malononitrile (**12c**)

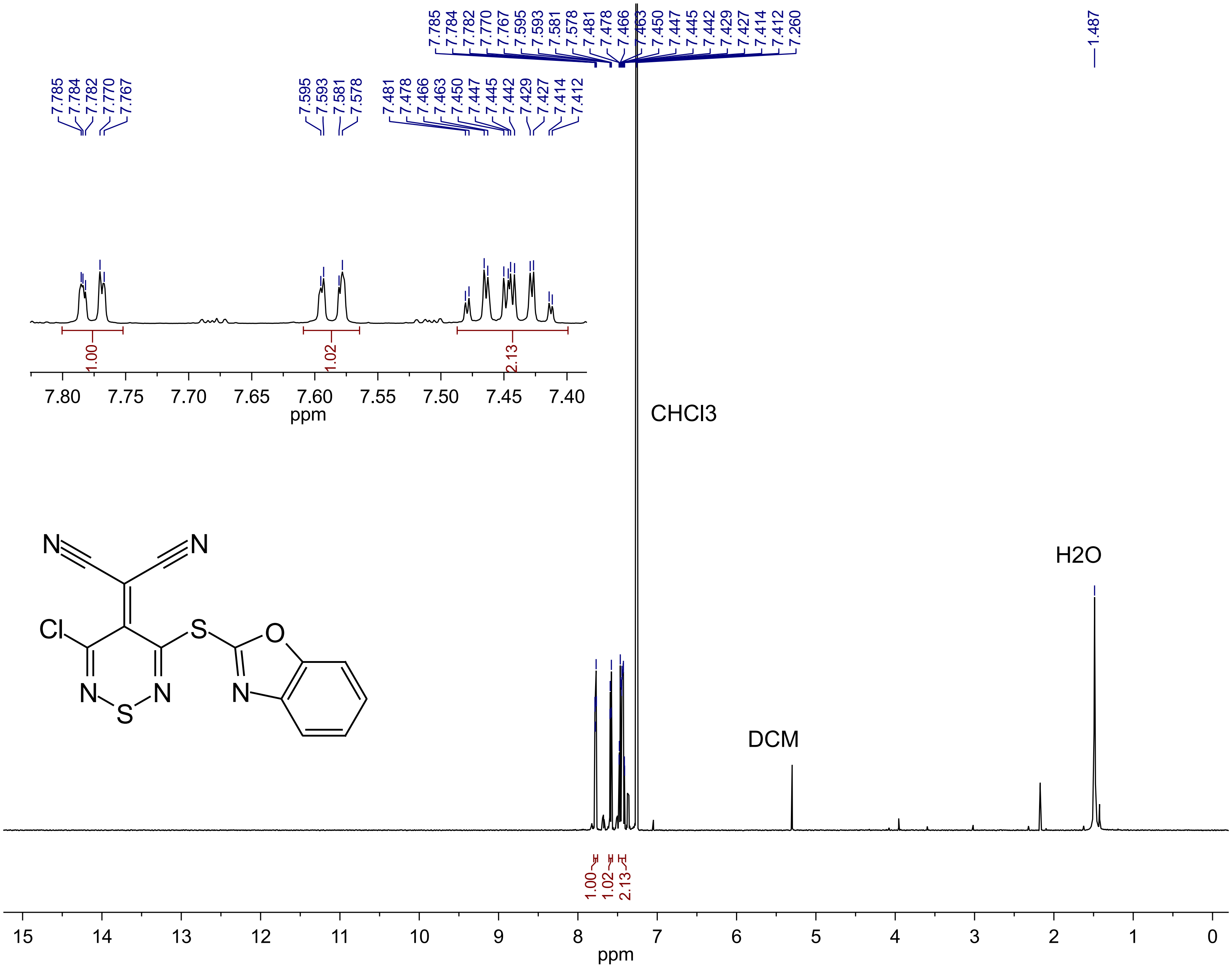

Current Data Parameters
NAME Kalogirou
EXPNO 746
PROCNO 1

F2 - Acquisition Parameters
Date_ 20191220
Time 9.40
INSTRUM spect
PROBHD 5 mm PABBO BB-
PULPROG zg30
TD 65536
SOLVENT CDCl3
NS 16
DS 2
SWH 10000.000 Hz
FIDRES 0.152588 Hz
AQ 3.2767999 sec
RG 203
DW 50.000 usec
DE 6.50 usec
TE 295.8 K
D1 1.00000000 sec
TD0 1

======== CHANNEL f1 ========
SFO1 500.0361158 MHz
NUC1 1H
P1 12.00 usec
PLW1 14.50000000 W

F2 - Processing parameters
SI 65536
SF 500.0330402 MHz
WDW EM
SSB 0
LB 0.30 Hz
GB 0
PC 1.00

^{13}C NMR of 2-[3-(benzo[*d*]oxazol-2-ylthio)-5-chloro-4*H*-1,2,6-thiadiazin-4-ylidene]malononitrile (**12c**)

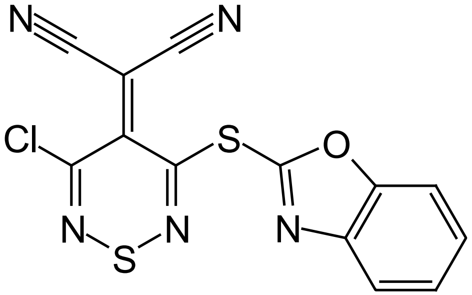

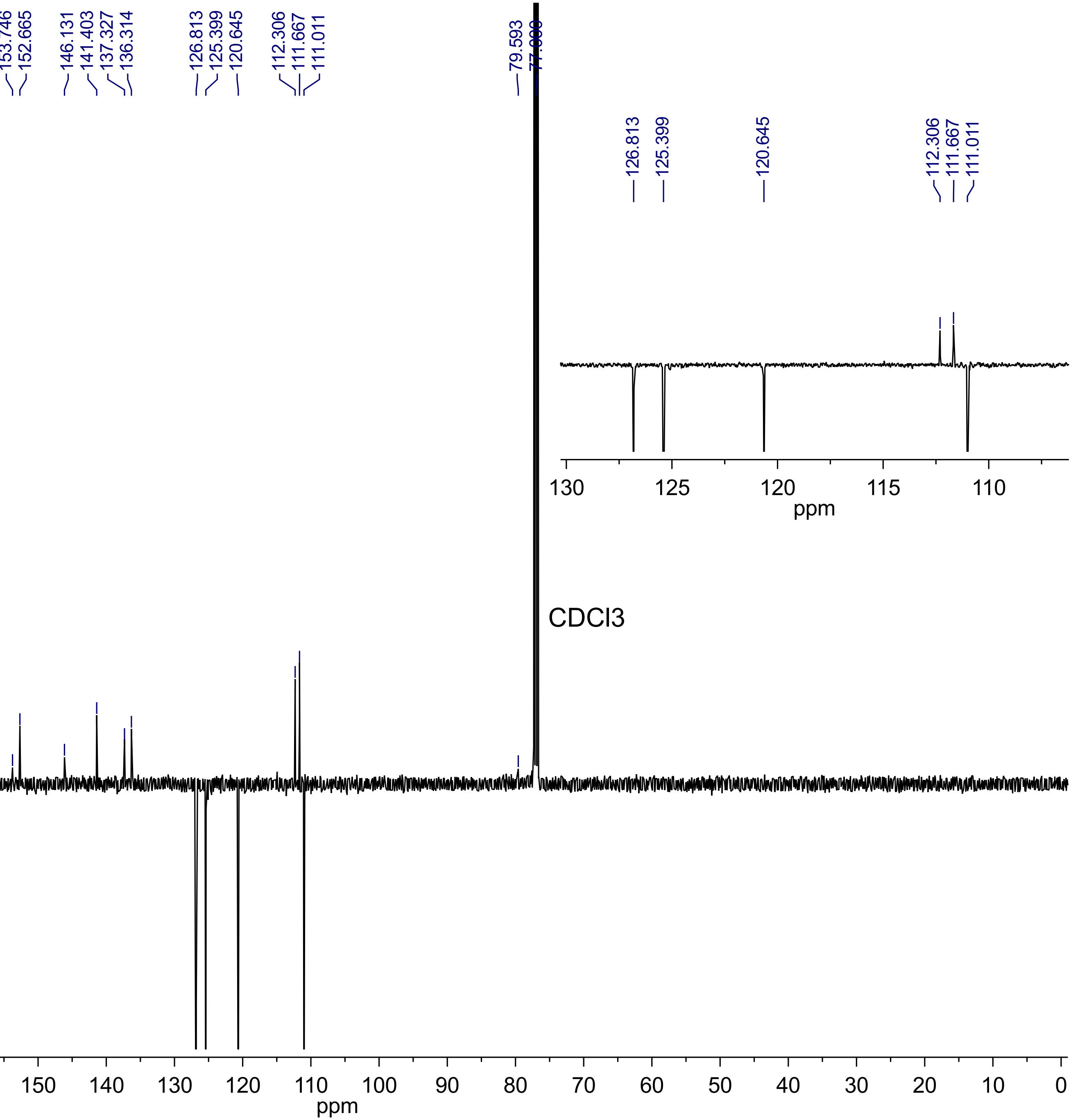

Current Data Parameters
NAME Kalogirou
EXPNO 760
PROCNO 1
F2 - Acquisition Parameters
Date_ 20191220
Time 9.09
INSTRUM spect
PROBHD 5 mm PABBO BB-
PULPROG jmod
TD 65536
SOLVENT CDCl3
NS 14800
DS 4
SWH 29761.904 Hz
FIDRES 0.454131 Hz
AQ 1.1010048 sec
RG 2050
DW 16.800 usec
DE 6.50 usec
TE 297.3 K
CNST2 145.0000000
CNST11 1.0000000
D1 2.00000000 sec
D20 0.00689655 sec
TD0 1
======== CHANNEL f1 ========
SFO1 125.7459782 MHz
NUC1 13C
P1 9.20 usec
P2 18.40 usec
PLW1 140.00000000 W
======== CHANNEL f2 ========
SFO2 500.0350280 MHz
NUC2 1H
CPDPRG[2 waltz16
PCPD2 80.00 usec
PLW2 14.50000000 W
PLW12 0.32624999 W
F2 - Processing parameters
SI 32768
SF 125.7334080 MHz
WDW EM
SSB 0
LB 1.00 Hz
GB 0
PC 1.40
